# Quantitative matching of crystal structures to experimental powder diffractograms†

**DOI:** 10.1039/d3sc00168g

**Published:** 2023-04-04

**Authors:** R. Alex Mayo, Katherine M. Marczenko, Erin R. Johnson

**Affiliations:** a Department of Chemistry, Dalhousie University 6274 Coburg Road Halifax NS B3H 4R2 Canada erin.johnson@dal.ca; b University of Guelph 50 Stone Rd. E Guelph ON N1G 2W1 Canada kmarczen@uoguelph.ca

## Abstract

The identification and classification of crystal structures is fundamental in materials science, as the crystal structure is an inherent factor of what gives solid materials their properties. Being able to identify the same crystallographic form from unique origins (*e.g.* different temperatures, pressures, or *in silico*-generated) is a complex challenge. While our previous work has focused on comparison of simulated powder diffractograms from known crystal structures, herein is presented the variable-cell experimental powder difference (VC-xPWDF) method to match collected powder diffractograms of unknown polymorphs to both experimental crystal structures from the Cambridge Structural Database and *in silico*-generated structures from the Control and Prediction of the Organic Solid State database. The VC-xPWDF method is shown to correctly identify the most similar crystal structure to both moderate and “low” quality experimental powder diffractograms for a set of 7 representative organic compounds. Features of the powder diffractograms that are more challenging for the VC-xPWDF method are discussed (*i.e.* preferred orientation), and comparison with the FIDEL method showcases the advantage of VC-xPWDF provided the experimental powder diffractogram can be indexed. The VC-xPWDF method should allow rapid identification of new polymorphs from solid-form screening studies, without requiring single-crystal analysis.

## Abstract 2

The variable-cell experimental powder difference (VC-xPWDF) method allows rapid screening of both experimental and *in silico* generated crystal structures to identify matches to experimental powder X-ray diffractograms of unknown polymorphs.

## Graphical abstract

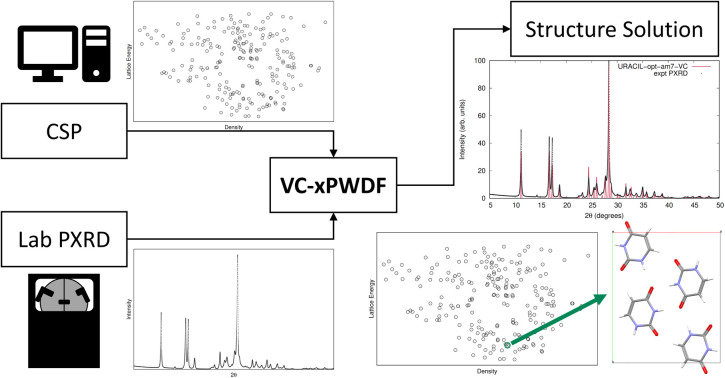

## 1 Introduction

Powder X-ray diffraction (PXRD) is a workhorse characterization technique in biology, chemistry, physics, and engineering. It has become an invaluable tool in industrial quality control, research and development, and academia for phase identification, quantification, and the characterization of polymorphs.^1^ While PXRD is the easiest and fastest method for obtaining fundamental information about the solid-state structure of a material, single-crystal X-ray diffraction (SC-XRD) remains the gold standard for comprehensive data on the molecular structure and periodic arrangement in three-dimensional space.

Structure determination from powder data (SDPD) is also an active and practiced method of crystal structure determination. However, high-quality powder X-ray diffraction data and access to an expert crystallographer are likely requirements, and the methods used often involve more time, constraints, and trial and error than SC-XRD before achieving success for molecular organic crystals.^2–4^ The statistical assessment of whether a proposed crystal structure can generate the powder diffractogram that is observed experimentally is done by Rietveld refinement. This non-linear least-squares refinement procedure modifies various parameters of the proposed crystal structure model and experimental conditions in order to maximize agreement between the simulated powder diffractogram and the experimental one. Rietveld refinement results in final (dis)agreement metrics, such as the weighted profile residuals (*R*_wp_) and chi-squared (*χ*^2^). While debate over how to interpret the refinement metrics is not new,^5^ recent publication of four unique crystal structure models that are able to yield a reasonable refined fit to a powder diffractogram obtained from synchrotron X-ray diffraction has highlighted the inherent ambiguity that may accompany a structure solution from powder diffraction data.^6^

Crystal structure prediction (CSP) uses theoretical and physical chemistry to deduce the crystal structure(s) of a given molecule or elemental composition.^7^ CSP has become notable in material science as an aid to the development of porous solids^8,9^ and organic semiconductors,^10^ among other materials with desirable properties.^11,12^ In particular, the pharmaceutical industry sees relatively common use of CSP for drug substance development and risk reduction.^12,13^ A CSP study on a new active pharmaceutical ingredient (API) can begin as soon as the discovery team identifies it as a viable candidate, either theoretically or experimentally,^14,15^ and can predict a late-appearing polymorph, aid in the determination of crystal structures, and assess the API's propensity to form solvates.^16,17^ Sometimes, CSP will predict a more stable crystal structure than those that have been observed experimentally for an API and additional screening experiments may be performed in order to identify the conditions that yield this crystal structure, if it can be formed at all.^18^

The solids generated by crystallization experiments during polymorph screening are primarily evaluated by PXRD as the initial characterization tool. If a new PXRD pattern is observed, further characterization will ensue and there may be a need for a full structure determination using SC-XRD.^19^ However, if a CSP study has already been performed, it is likely that any new polymorphs characterised by PXRD are already represented within the tens of thousands of hypothetical crystal structures generated. It would, therefore, be highly desirable to identify which of these candidates is a match to an experimental diffractogram of a new polymorph.

Crystal structures collected under the same conditions (*i.e.* temperature and pressure) are generally easily classified as matching or different structures by comparison of their powder diffractograms by examining the peak positions and intensities. However, once the experimental conditions differ, or one of the two crystal structures is generated/optimized computationally, the comparison becomes problematic due to the condition-induced deviation in the lattice parameters (pressure-induced contraction, thermal expansion, neglect of zero-point vibrations for “static lattice” structures optimized using computational methods, *etc*…). Even minor changes in the lattice dimensions result in notable shifts in the powder diffractogram. This is a common problem, as routine PXRD measurements occur at room temperature, whereas routine SC-XRD measurements are made at temperatures as low as 80 K.

Several corrections have been developed to account for the effect of lattice dimension deviations during quantitative crystal structure comparison based on simulated PXRD patterns. These include an isotropic volume correction,^20^ the variable-cell powder difference (VC-PWDF) method,^21^ and the FIt with DEviating Lattice (FIDEL) method.^22^ In this work, we will focus on the VC-PWDF method, which converts input crystal structures to their Niggli reduced cells, then screens possible unit cell bases that may be coincident with the given reference structure, and deforms each candidate unit cell basis to identify the matching cell, if one exists. It then yields the measure of dissimilarity of the best matching cell with the triangle-weighted cross-correlation function proposed by de Gelder *et al.*^23^ The VC-PWDF method has been shown to be as successful as the COMPACK^24^ method, which compares crystal structures based on atomic positions.^21,25^

Notably, the VC-PWDF method has shown excellent performance for comparison of simulated diffractrograms from *in silico* structures and those obtained from SC-XRD collected under different experimental conditions.^25^ Thus, it forms an ideal basis for a new, high-accuracy method for comparing the experimental powder diffractograms collected during a high-throughput screening to the crystal structures obtained from a CSP study. While the FIDEL method has shown some efficacy in this realm,^6,22^ the minimization protocol can be a lengthy procedure and is prone to errors due to local minima (*vide infra*). Therefore, we look to apply the VC-PWDF method to tackle this problem with improved accuracy and consistency of outcome.

**Fig. 1:**
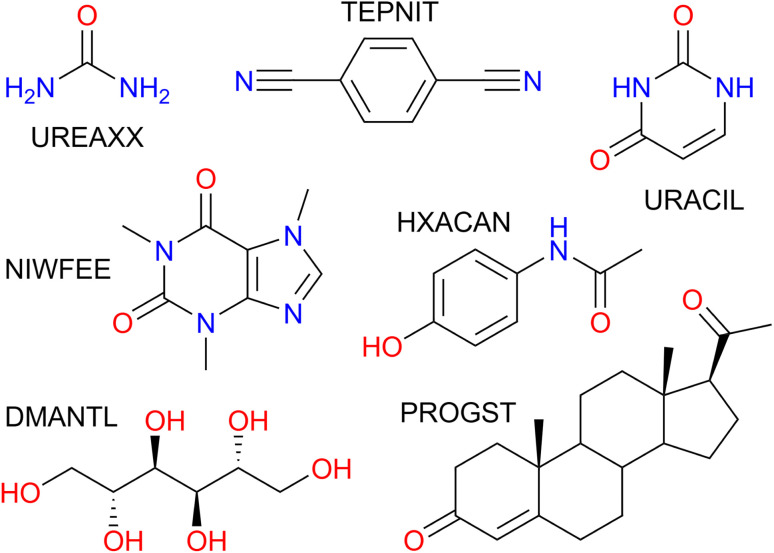
Compounds studied and accompanying CSD refcode family. UREAXX: urea; TEPNIT: 1,4-dicyanobenzene; URACIL: uracil; NIWFEE: caffeine; HXACAN: acetaminophen; DMANTL: d-mannitol; PROGST: (+)-progesterone.

Herein, we report the modification of our VC-PWDF method to enable direct comparison of ideal simulated powder diffractograms for known crystal structures with experimentally collected data for an unknown polymorph. The primary goal is to enable crystal structure identification from an experimental powder pattern, given a list of putative crystal structures generated computationally. The method was applied to seven example compounds (Fig. 1) for which PXRD patterns were collected on a standard laboratory instrument. The experimental results were compared with simulated powder diffractograms calculated from both known experimental crystal structures (Cambridge Structural Database, CSD^26^), and *in silico* generated crystal structures (Control and Prediction of the Organic Solid State, CPOSS, database^27^). Our method was found to consistently identify the correct crystallographic form from the relevant database(s) as the structure matching the experimental powder diffractogram based on minimum powder difference scores.

## 2 Results and discussion

The VC-PWDF method was modified in two distinct ways. First, the code was changed to accommodate experimental powder diffractogram data (intensity *vs.* 2*θ* in degrees as a .xy file) and unit-cell dimensions as one of the inputs used for comparison. The other input is a crystal structure file from which the ideal powder diffractogram is simulated for comparison. Next, the code was modified to perform a basic normalization of the experimental PXRD data in the .xy file by subtracting the lowest intensity value recorded in the experimental powder diffractogram from all data-points and scaling the highest intensity peak to a value of 100. No further processing of the diffractograms or consideration of sample, instrument, or diffraction conditions was performed. In order to distinguish the results that come from the comparison of two simulated powder patterns (VC-PWDF method/score) from those that compare a simulated powder pattern with an experimental one, we will use VC-xPWDF method/score for the latter. The method is available within the developers version of critic2.^28^ While a more complex baseline correction may be required to see similar performance to that observed herein if the PXRD were collected using a different experimental set-up (*e.g.* capillary in transmission mode), this processing could be done prior to analysis with the VC-xPWDF method.

We obtained the experimental powder diffractograms of seven chemicals that were readily available at the University of Guelph (urea, 1,4-dicyanobenzene, uracil, caffeine, acetaminophen, d-mannitol, (+)-progesterone). The collected powder diffractograms for each of the 7 compounds are shown in Fig. S1,† and we consider them to be of moderate quality. The 20 most intense peaks were picked and used as input for indexing with the CrysFire2020 suite,^29^ which facilitates running of multiple indexing algorithms (including TAUP,^30^ ITO,^31^ TREOR,^32^ KOHL,^33^ and DICVOL^34^). The powder diffractogram of urea contained only 13 well-defined peaks, so only these 13 peaks were used for indexing. The caffeine diffractogram contained many peaks, some of which became quite broad beyond 2*θ* = 30°, so the 24 most intense peaks observed before this angle were used for indexing. The cell dimensions with the highest De Wolff's^35^ figure of merit (summarized in Table S2†) were used as input to the VC-xPWDF method, accompanying the experimental powder diffractogram.

Lists of *in silico* generated structures for the compounds studied were obtained from the CPOSS database. These structure-energy landscapes were screened for duplicate crystallographic forms and structure(s) matching the known experimental structure(s) present in the CSD. Details regarding these data are provided in the ESI.† The landscape for progesterone includes mostly racemic crystal structures, with only 8 of the 149 crystal structures being enantiopure. The landscape for mannitol provides a more equal number of enantiopure structures of nearly 50% (250/546). All other molecules are achiral and so the presence or absence of reflection symmetry elements in the crystal lattices is not of concern in this study. In addition to the *in silico* generated structures, SC-XRD determined structures of one or more known polymorphs of the 7 compounds were obtained from the CSD, with data collected over a range of temperatures (see Table 1 for the refcodes). It should be noted that the VC-PWDF method is currently unable to work with disordered structures, so any such polymorphs were omitted for this work. In particular, the crystal structure NIWFEE03 (*Z*′ = 5 and *Z* = 20) is the only non-disordered structure of caffeine in the CSD. While the disordered *C*2/*c* structure for the β polymorph is the correct structure solution,^36^ its simulated powder patterns is nearly indistinguishable from that of the ordered Cc structure (NIWFEE03), which was used to represent the beta form of caffeine throughout this study.

**Table 1:** VC-xPWDF scores from comparison of the collected powder diffractograms with the CSD structures^a^

CSD refcode	Conditions	Form	VC-xPWDF
UREAXX07 (ref. 37)	123 K	I	0.0335
UREAXX11 (ref. 38)	60 K	I	0.0337
UREAXX12 (ref. 39)	12 K	I	0.0339
UREAXX23 (ref. 40)	Ambient	I	0.0364
UREAXX26 (ref. 41)	3.1 GPa	IV	0.2087
UREAXX33 (ref. 42)	1.0 GPa	III	0.2528
TEPNIT04 (ref. 43)	Ambient	*β*	0.0326
TEPNIT14 (ref. 44)	100 K	*β*	0.0330
TEPNIT06 (ref. 45)	Ambient	*α*	0.4339
URACIL (ref. 46)	Ambient	—	0.0290
NIWFEE03 (ref. 47)	Ambient	*	0.0114
HXACAN35 (ref. 48)	Ambient	I	0.0494
HXACAN04 (ref. 49)	150 K	I	0.0602
HXACAN15 (ref. 50)	80 K	I	0.0633
HXACAN13 (ref. 50)	20 K	I	0.0670
HXACAN09 (ref. 51)	1 GPa	I	0.0772
HXACAN47 (ref. 52)	200 K	VII	0.4099
HXACAN40 (ref. 53)	Ambient	III	0.5764
HXACAN33 (ref. 54)	Ambient	II	0.7293
DMANTL15 (ref. 55)	100 K	*β*	0.0962
DMANTL07 (ref. 56)	Ambient	*β*	0.0992
DMANTL08 (ref. 57)	100 K	*α*	0.3145
DMANTL14 (ref. 58)	Ambient	*δ*	0.4352
PROGST12 (ref. 59)	150 K	I	0.0416
PROGST10 (ref. 60)	Ambient	I	0.0428
PROGST13 (ref. 59)	150 K	II	0.3426

a (—) URACIL has only one known crystal structure and (*) NIWFEE03 is an ordered description of the β form of caffeine.

The CSD structure refcodes, experimental conditions under which the measurements were made, and resulting VC-xPWDF scores from comparison with the experimental PXRD patterns are summarized in Table 1. For all 7 compounds studied, the CSD structures corresponding to the same polymorph as the experimental PXRD pattern are found to give the smallest VC-xPWDF score, regardless of the conditions under which they were obtained. Comparison of the collected powder patterns with CSD structures of different polymorphs results in much higher VC-xPWDF scores (Table 1). Additionally, the VC-xPWDF method was able to identify the matching structure for urea and (+)-progesterone, even though the indexed cell parameters obtained from their experimental powder diffractograms are not the same as (or an obvious sub/super-cell of) the matching CSD structure (Table S2†). The VC-xPWDF method is perfectly suited to make use of a viable indexed unit cell from an experimental powder diffractogram, whether it is a supercell, subcell, or some non-standard description of the same lattice, due to its exploration of viable unit cells for each crystal structure. This should be useful for *in situ* PXRD, to determine if a phase transition occurs with changes in temperature and/or pressure.

**Fig. 2:**
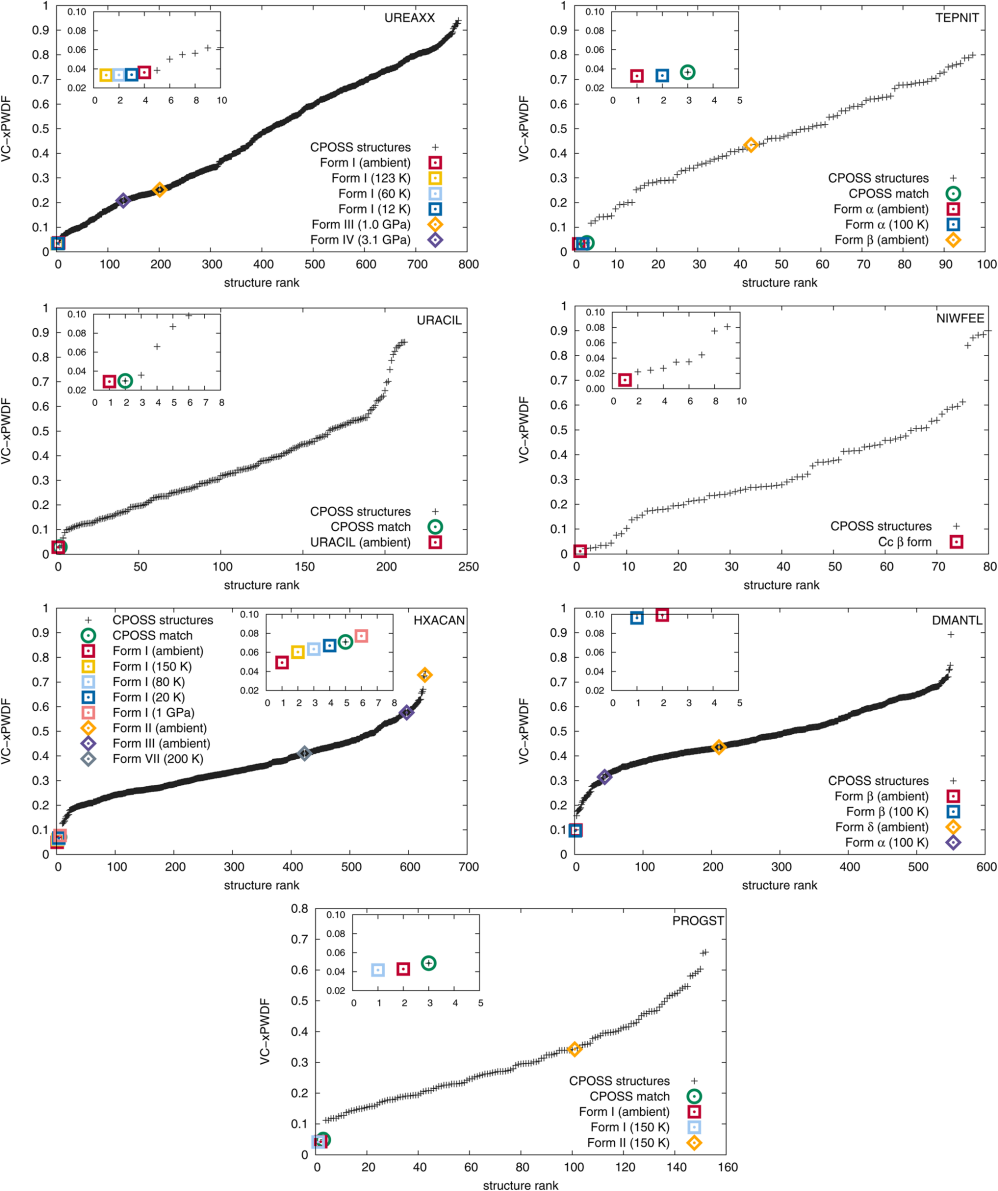
Plots showing the computed VC-xPWDF scores resulting from comparison of each input crystal structure to the experimental powder diffractogram collected for that compound. The structures are ranked by lowest VC-PxWDF score (most similar) and the insets provide views of the best matching structures with VC-xPWDF scores <0.1, up to a maximum of 10 for clarity. The point types indicate the source of each crystal structure: squares correspond to CSD structures of the same polymorph as the sample studied, diamonds are different polymorphs of that compound from the CSD, and +signs are *in silico* generated structures from the CPOSS database. The CPOSS structure that corresponds to the same polymorph as the experimental sample (if a matching structure was generated) is identified with a green circle around that data point.

The results of comparisons between the experimentally obtained diffractograms and those simulated from the crystal structures in both the CPOSS database and CSD are shown in Fig. 2. These results clearly demonstrate the ability of the VC-xPWDF method to identify the correct polymorph from the known forms of these compounds in all cases. Further, the results in Fig. 2 also show that, if a matching CPOSS structure exists, this structure is consistently ranked just after/amongst the experimental structure(s) for that polymorph. These rankings showcase the ability of the VC-xPWDF method to identify the most similar *in silico* generated structure according to the powder difference score as well.

For 1,4-dicyanobenzene, acetaminophen, d-mannitol, and (+)-progesterone, the plots in Fig. 2 show good separation between the matching and non-matching structures. In these cases, there is clarity in which of the structure(s) match(es) the experimental powder diffractogram, and which structures do not. Additionally, all matching structures from both the CSD and CPOSS database yield VC-xPWDF scores of less than 0.1 when compared to the experimental powder diffractogram. However, the plots for urea, uracil, and caffeine show multiple structures with VC-xPWDF scores less than 0.1. Based on the results for our small data set, we propose that a structure with a VC-xPWDF score below 0.1 is grounds to consider it a potential match, but does not guarantee it. This will of course vary with the quality of the powder diffractogram as well (*vide infra*).

For the case of urea, the reason that such a large number (42) of CPOSS structures have VC-xPWDF scores <0.1 can be explained by the fact that the powder diffractogram is dominated by a single, high-intensity peak. Thus, if a candidate structure also has a peak at this position, much of the diffractogram intensity is already overlaid, resulting in a low powder difference score. For this compound, a quick glance at the diffractogram overlays quickly eliminates the non-matching structures and makes it evident that the CPOSS landscape does not include the experimental polymorph (Fig. S4†). The visual comparisons with the caffeine overlays (Fig. S5†) tell a similar story; there are a couple positions of high intensity peaks in the diffractogram, and low powder difference scores can still be obtained from cases where the remaining small intensity peaks do not overlap well.

The three *in silico* generated crystal structures of uracil with VC-xPWDF scores between 0.06–0.1 can also be reasonably discounted as matches with a visual assessment; however, structure ID am82 (VC-xPWDF score of 0.0358) cannot (Fig. S6†). Even agreement values from Rietveld refinement are insufficient to exclude the possibility of am82 being a match to the experimental powder diffractogram (Table S4†). Comparing the two *in silico* generated crystal structures am7 (matching crystal form) and am82 to one another yields a VC-PWDF score of 0.0238 and the distorted structures obtained after processing with the VC-xPWDF method to match the experimental powder diffractogram yield a 20/20 match with RMSD(20) = 0.247 Å with COMPACK (default tolerances). The similarity of the packing of the uracil molecule in these two (modified) crystal structures is shown in Fig. S7,† and we would expect them to converge to the same structure after geometry optimization with density-functional theory.

**Fig. 3:**
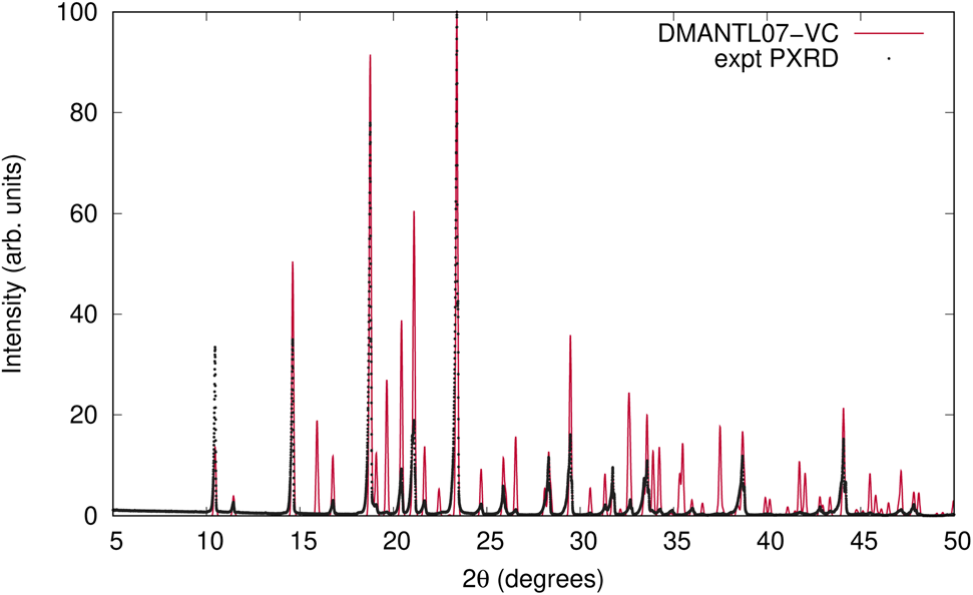
Overlay of the experimentally collected powder diffractogram and the simulated powder diffractogram for DMANTL07 after the VC-xPWDF protocol.

The VC-xPWDF scores obtained from comparison of the matching crystal structures to the collected powder diffractogram of d-mannitol are considerably higher than for the other compounds investigated. The overlay of the simulated PXRD pattern for DMANTL07 with the collected experimental diffractogram is shown in Fig. 3. Based on this overlay, the reason for the higher scores can be clearly attributed to preferred orientation (the biased orientation of one or more crystallographic planes in the experimental sample) leading to a change in relative intensities of the peaks. Because the POWDIFF score considers differences in the peak intensities in addition to their position, a significant deviation from the ideal diffractogram will yield a higher score.

As is to be expected, the quality of the powder diffractogram has an effect on its measured similarity to an ideal simulated powder diffractogram. It is interesting that peak shapes, a flat baseline, and other features commonly associated with “high quality” PXRD data are rather easily obtained in adequate quality, and/or have a relatively minimal effect on the resultant similarity scores measured within this dataset (see Fig. S2† for all diffraction patterns) when compared to the considerable effect from preferred orientation.

To test the degree to which the results would change with “lower quality” data, quick 2 minutes scans of the prepared samples were collected. The results are nearly indistinguishable from those obtained with the “higher quality” data from the 3 hours scans. The analogous plots to Fig. 2 using the screening-scan diffractograms are shown in Fig. S8.† Provided the diffractogram yielded by a 2 minutes screening scan can provide a valid indexed unit cell, this data is perfectly acceptable for comparison with the VC-xPWDF method. Clearly, this is ideal as a complement to high-throughput polymorph screening in order to identify the crystal structures of the various forms analyzed by short screening PXRD data collection, provided one has access to a CSP landscape.

Rietveld refinement is a common final step when assessing whether a proposed crystal structure model matches an experimentally collected powder diffractogram. Accordingly, we have performed Rietveld refinement on an assortment of CPOSS and CSD structures that yielded low VC-xPWDF scores using the automated BGMN protocol^61^ implemented in the Profex software.^62^ The outcomes are tabulated in Table S3.† When refining an experimental crystal structure from the CSD to the experimental powder diffractogram, the best *R*_wp_ values were obtained by starting from the CSD structures solved from data collected under ambient conditions. Refinement attempts starting with CSD structures of the matching form collected at high pressure or low temperature almost always yielded overlays where peaks were misaligned. The application of the VC-xPWDF method to the CSD structures determined under ambient conditions improved the agreement factors considerably (Section S4.1†), highlighting the utility of the VC-xPWDF method in providing the best starting point for a Rietveld refinement.

It would be ideal if Rietveld refinement could be used to confirm or rule out structures with low VC-xPWDF scores as a match to the experimental polymorph. However, within this dataset, the absolute *R*_wp_ and *χ*^2^ values provide little additional evidence in deciding whether or not a crystal structure matches the experimental powder diffractogram. Many refinements give poor peak overlays, but still yield *R*_wp_ values in the 20–30% range, while many successful refinements with good peak overlays yield *R*_wp_ values that are <40%. It may be the case that a more tailored approach is required to fully and carefully refine these data, or that the PXRD data collected are simply not of high enough quality for conclusive refinement. Data collection at a synchroton source may eliminate the issues outlined in the latter case. This again highlights the advantage of the VC-xPWDF method as it appears to provide information equivalent to Rietveld refinement without requiring specialized expertise, or very high quality PXRD patterns as input.

A current drawback of the VC-xPWDF method is its requirement of input unit-cell dimensions for the reference structure. Thus, for experimental powder diffractograms, indexing is a must. Conversely, a major advantage of the FIDEL method is that it can run successfully without knowledge of unit-cell dimensions. With the use of the autoFIDEL code,^63^ we applied the FIDEL method to our dataset (Fig. S9, S10 and Tables S7–S13†). FIDEL is able to identify the matching polymorph from the CSD (determined under ambient conditions) for all cases except acetaminophen (HXACAN) using the default run parameters. Because the minimization protocol of FIDEL is a more computationally expensive approach to aligning the diffractogram peak positions, the program sets a minimum initial agreement that must be met in order for the protocol to run, the default is a POWDIFF score <0.7. The initial agreement between the simulated powder pattern of HXACAN35 and our collected powder diffractogram is a POWDIFF score of 0.7325, and so the POWDIFF score of 0.1383 post-minimization is only obtained if the default parameters are modified to allow the optimization. With this adjustment in the run parameters, HXACAN35 is correctly identified as the best matching crystal structure with autoFIDEL.

The default run parameters are the reason the rank-plots of the FIDEL results (Fig. S9†) only include a fraction of the total number of structures ranked by our VC-xPWDF method, as only the structures that undergo the minimization protocol are included with their accompanying post-minimized POWDIFF score. Even with the reduction in the number of structures run by autoFIDEL, some notable differences are identified for the optimization of the *in silico* generated matching crystal structures of acetaminophen and 1,4-dicyanobenzene. The most extreme example is the latter.

**Fig. 4:**
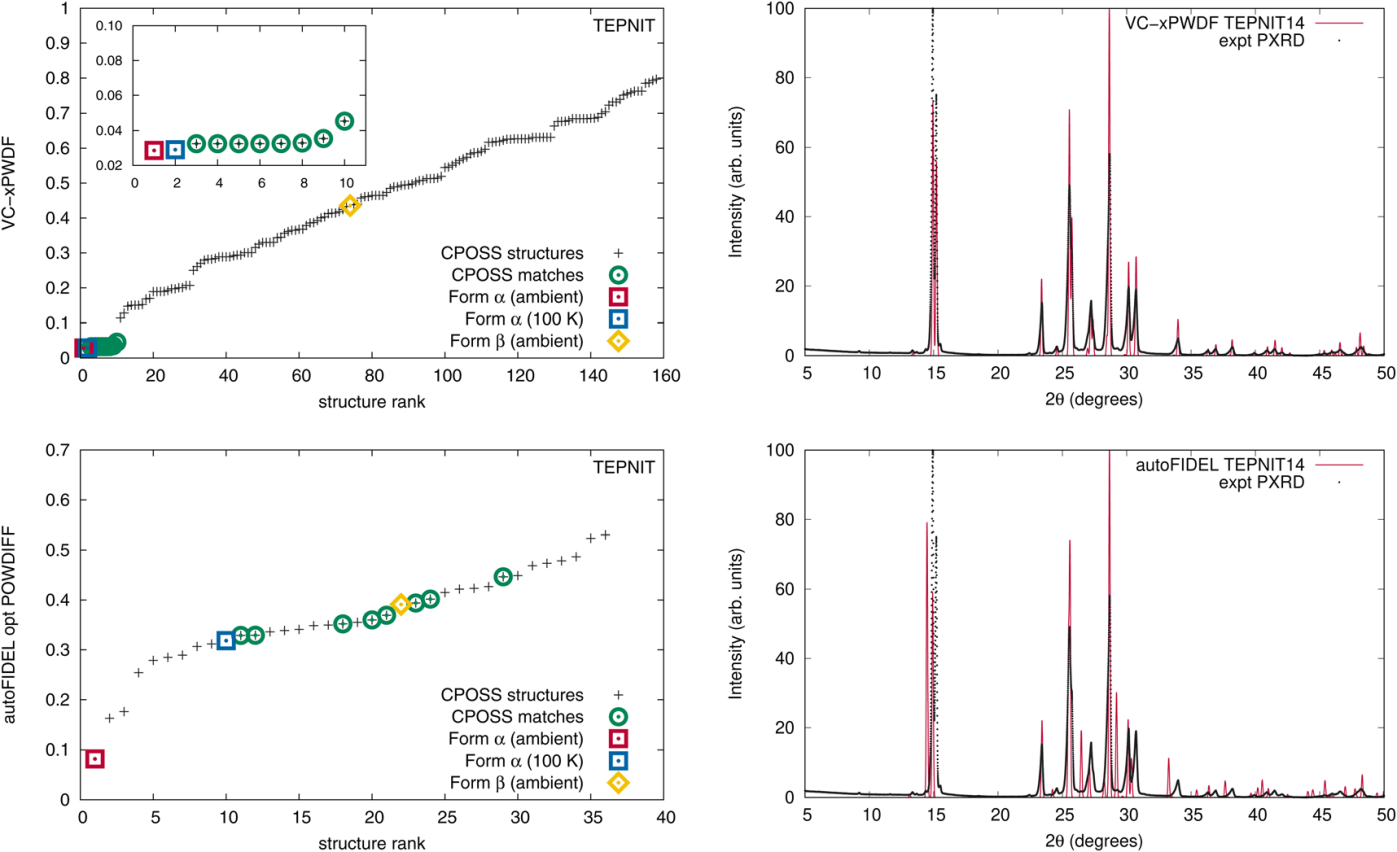
Left: (VC-x)PWDF scores resulting from comparison of CSD and CPOSS input structures with the experimental powder diffractogram for 1,4-dicyanobenzene computed using VC-xPWDF (top), and autoFIDEL (bottom). Right: overlays of the experimental and simulated TEPNIT14 powder diffractogram after correction with VC-xPWDF (top), showing perfect alignment of the peak positions, and after minimization with autoFIDEL (bottom), which leaves many peaks poorly positioned.

When the original CPOSS structure list for 1,4-dicyanobenzene (containing duplicates) is screened against the experimental powder diffractogram by the VC-xPWDF method, the several equivalent matching structures are identified and grouped together at the lowest powder difference score (Fig. 4, top-left). Conversely, the same screening using autoFIDEL ranks the matching structures haphazardly at various powder difference scores (Fig. 4, bottom-left). Even the experimental structure that is determined at 100 K (TEPNIT14) is not minimized to a low POWDIFF score with autoFIDEL. Thus, if no ambient temperature crystal structure solution was available for comparison, FIDEL would fail to identify the matching polymorph, despite multiple descriptions of it being present in the list of structures being screened.

While cases analogous to 1,4-dicyanobenzene and acetaminophen may be relatively few, we showcase here an example of the minimization protocol used in autoFIDEL getting caught in a local minimum. The result is misaligned peaks and the inability to identify the matching crystal structure. For comparison, the overlays of the experimental powder diffractogram and simulated diffractogram of the TEPNIT14 structure after modification by both the VC-xPWDF method and autoFIDEL are also presented in Fig. 4. The advantage of the VC-xPWDF method, provided the experimental diffractogram can be indexed, is that it will correctly align the peak positions of the simulated powder diffractogram of the matching structure directly.

## 3 Conclusion

In this work, we illustrated the ability of the VC-xPWDF method to clearly identify the most similar crystal structure to both moderate and “low” quality experimental powder diffractogram for a set of 7 representative organic compounds. In all cases, matching SC-XRD structures obtained from the CSD were identified by having the lowest VC-xPWDF scores of any crystal structures searched. As competing polymorphs consistently yielded much higher VC-xPWDF scores, the method is able to rapidly identify which of several literature polymorphs matches an experimental sample, even if the structure was solved for very different temperature and pressure conditions.

The modification of the VC-PWDF method to allow an experimental PXRD pattern as input has converted it from being a tool exclusively used for the comparison of solved/complete crystal structures to one of a select few methods that is able to quantitatively assign a crystal structure to an experimentally collected powder diffractogram. The various other PXRD-based methods for the comparison of crystal structures show poor performance in general because of thermal expansion/pressure induced contraction, and thus are generally ineffective in the assignment of the matching *in silico*-generated structure to a powder diffractogram that is collected under screening-like conditions (*e.g.* 2 minutes scan at room temperature). The VC-xPWDF method directly address this research problem.

The principle limitation of the VC-xPWDF method is that it must be provided with valid indexed unit-cell parameters to accompany the experimental powder diffractogram. Therefore, the method cannot be applied if the experimental diffractogram cannot be successfully indexed. This stands in contrast to the FIDEL method, which does not require indexing. However, we have provided an example here of the risks involved with the FIDEL approach, and the advantages of using the VC-xPWDF method when the indexed unit cell parameters can be determined. Future development of the VC-PWDF method will seek to eliminate the requirement of the indexed unit-cell parameters.

The broader utility of the VC-xPWDF method would be to identify a previously uncharacterized crystal structure from a list of candidates generated during first-principle crystal structure prediction. This would be of particular value to the pharmaceutical industry for polymorph screening, as well as in the development of porous solids and organic electronics, and for other materials research where design using CSP might be applied. Here, for all 4 cases where a list of *in silico* generated structures contained a match to the experimental polymorph, the VC-xPWDF method successfully identified the matching structure(s) as having the lowest powder difference score of the candidates. However, for an unknown compound, there is no guarantee that a CSP landscape will contain the experimental polymorph, so there remains the issue of confidence that the structure with the lowest VC-xPWDF score is the actual matching structure. Similar to Rietveld refinement, a small powder difference score (<0.1) does not always provide conclusive evidence that the proposed crystal structure matches the experimental powder diffractogram. However, a visual assessment of the diffractogram overlay, which is also a recommendation following Rietveld refinement, can provide increased confidence in the result.

In practice, CSP studies typically use force-field methods for structure generation. However, since the relative energies from force-field methods are often poor, a re-ranking of up to several hundred low-energy structures may be performed using dispersion-corrected density-functional theory (DFT) methods to provide a more accurate energy landscape. Thus, additional confidence in deciding which, if any, of several candidate structures with low VC-xPWDF scores is the experimental match could be gained by also considering the relative DFT energies. Structures with both low energy and low VC-xPWDF scores are more likely matches, while candidates with a low VC-xPWDF score but high relative energy would be less likely to correspond to the experimental polymorph. In future work, we will consider combining the VC-xPWDF method with such CSP information to solve unknown crystal structures from powder data.

## Data availability

The data that support this study are provided in the ESI† or are available from the authors upon reasonable request.

## Author contributions

RAM performed the VC-xPWDF calculations, KMM and RAM performed the experimental PXRD measurements and analysis. All authors were involved in conceptualization of the study and writing of the manuscript.

## Conflicts of interest

There are no conflicts to declare.

## Supplementary Material

SC-014-D3SC00168G-s001

SC-014-D3SC00168G-s002
