# Supplementary material for: Quantitative matching of crystal structures to experimental powder diffractograms
Source: Chem Sci. 2023 Apr 4;14(18):4777–85. doi: 10.1039/d3sc00168g (PMC10171065; doi:10.1039/d3sc00168g)
Supplement: SC-014-D3SC00168G-s002 [file SC-014-D3SC00168G-s002.pdf]

Electronic Supplementary Material (ESI) for Chemical Science.
This journal is © The Royal Society of Chemistry 2023

Supplementary information for: Quantitative matching of crystal structures to experimental powder diffractograms

R. Alex Mayo, Katherine M. Marczenko, and Erin R. Johnson

S1 Experimental

S1.1 Powder X-ray diffraction

Acetaminophen (Sigma Aldrich), caffeine (Alfa Aesar), 1,4-dicyanobenzene (Sigma Aldrich), D-mannitol (VWR), (+)-progesterone (Sigma Aldrich), uracil (Sigma Aldrich), and urea (Sigma Aldrich) were used as received. If the solid powder appeared to have a notably large crystallite size or shape then crushing with a spatula and/or grinding with mortar and pestle was done prior to analysis. PXRD measurements were performed using a PANalytical Empyrean diffractometer in reflection (Bragg-Brentano) geometry with a Cu $K\alpha$ radiation source ($\lambda = 1.54184$ A), Ni $K\beta$ filter, and PIXcel1D linear detector. A powdered sample was back-loaded into a sample holder with a 16mm insert, which was mounted on a spinning stage at room temperature.

Powder diffractograms were recorded in the 5 - 50 degrees 2θ range with either a step size of 0.00328 degrees and exposure time of 180 seconds per step ("moderate quality" 3 hour scan) or a step size of 0.01313 degrees and exposure time of 10 seconds per step ("lower quality" 2 minute scan). Data collection was controlled with the Data Collector software.[1]

S2 Data

S2.1 Target structures from the CSD

Target crystal structures were identified in the Cambridge Structural Database (CSD) by refcode. Where more than one entry for a given polymorph was available, the one collected at ambient conditions with the lowest R-value was chosen as the primary target used in comparisons. Structures from low temperature or high pressure data collections were used for comparisons where indicated. As disordered structures are not currently compatible with the VC-PWDF method, any such structures were not included in comparisons or analyses (eg. polymorphs of NIWFEE).

S2.2 CSP landscapes

Lists of *in silico* generated structures hosted in the CPOSS database were obtained from Dr. Louise Price. Some landscapes have been published (progesterone,[2] uracil,[3] 1,4-dicyanobenzene,[4] caffeine,[5]), while others have thus far remained unpublished (mannitol, urea, acetaminophen). While some differences may exist and reference to the publications (where available) is recommended for these fine details, the general approach followed for the generation of the *in silico* structures obtained from the CPOSS database are:

1. Generation and geometry optimization of various molecular conformers using GAUSSIAN.[6]

2. Calculation of atomic multipoles using distributed multipole analysis of the charge density calculated with GAUSSIAN for each conformer.[7]

3. Hypothetical crystal structure generation, restricted to a chosen set of space groups, Z, Z', and conformers. Possible programs used for this task include MOLPACK,[8] and CrystalPredictor[9].

4. Geometry minimization and energy evaluation of the generated crystal structure using the atomic multipoles for evaluation of the electrostatic interactions, and the Buckingham potential for dispersion interactions with Williams[10], FIT[11] atom-atom parameters. Possible programs used for this task include DMAREL,[12] or DMACRYS[13] (rigid molecule during minimization), and CrystalOptimizer[14] (flexible molecule during minimization).

The structure-energy landscapes were screened for duplicates with an in-house script that utilizes relative energy, VC-PWDF, and COMPACK (tolerance of ±30% and ±30° on distances and angles, respectively) to identify structures with $\Delta E < 2$ kJ/mol, VC-PWDF < 0.07, and 20/20 matches by COMPACK as duplicates.

Table S1: Summary of the result of duplicate screening on the structure-energy landscapes obtained from the CPOSS database. The number of candidates is the total number of structures received from the CPOSS database, and the unique number is the number of structures remaining after the duplicate screening.

Compound	CSD ID	Candidates	Unique
Urea	UREAXX	793	777
1,4-Dicyanobenzene	TEPNIT	144	94
Uracil	URACIL	217	211
Acetaminophen	HXACAN	640	618
Caffeine	NIWFEE	84	79
Mannitol	DMANTL	619	546
Progesterone	PROGST	149	149

S3 Computational methods

S3.1 VC-(x)PWDF

Our VC-PWDF method[15], which is implemented within the critic2 program[16], was modified in order to allow target unit cell dimensions (a, b, c, α, β, γ) and experimental powder diffractograms as a txt file (angle 2θ in degrees, and intensity) to be input for comparison against a simulated powder diffractogram generated by a crystal structure file (cif, res, etc...). We differentiate the results from the comparison of experimental and simulated diffractograms by including “x” before the PWDF portion of the method abbreviation, as in VC-xPWDF. Only diffractograms collected with Cu $K\alpha$ X-rays are compatible at this time. The VC-PWDF method uses the unit cell dimensions of the target structure (here, the indexed cell dimensions from the experimental powder diffractogram) and searches over all possible unit cell descriptions of the candidate structure for that which best matches the target diffractogram after replacing the unit cell parameters of the candidate unit cell with those of the target structure. The Figure of Merit (FoM) used is the dissimilarity value (POWDIFF) yielded by the triangle-weighted cross-correlation function described by de Gelder *et al.*.[17]

S3.2 autoFIDEL

A Python script written by Jonas Nyman to perform a variation of the FIDEL (Fit with DEviating Lattice parameters) protocol [18] was used. The FIDEL method uses a hill climber's (steepest descent) algorithm to minimize the difference between two powder diffractograms by variation of the unit cell parameters and atomic positions of the crystal structure that is used to generate the simulated powder diffractogram. The script used in this work only modifies the lattice parameters, no changes to atomic positions are affected during the optimization. The FoM output from autoFIDEL is the similarity value yielded by the triangle-weighted cross-correlation function described by de Gelder *et al.*.[17] In order to ease the comparison between FoM values, we have converted the similarity value to the dissimilarity (POWDIFF) by subtraction from one, since this is our preferred metric. Unless otherwise noted, the default parameters of autoFIDEL were used.

S4 Results

Table S2: Summary of indexed unit cell dimensions from the collected powder diffractograms and comparison to the unit cell dimensions of the matching polymorph in the CSD after conversion to their Niggli reduced unit cell. No space group determination was done for the experimental powder data.

Compound	Space group	a (Å)	b (Å)	c (Å)	α (°)	β (°)	γ (°)	V (Å^3)
UREAXX23(RT)	$P\bar{4}2_1m$	4.7042	5.6577	5.6577	90	90	90	150.579
Urea(PXRD)	-	7.987	7.987	9.4042	90	90	90	599.913
TEPNIT04(RT)	$P\bar{1}$	3.847	6.585	7.322	114.5	93.6	96.9	166.254
1,4-Dicyanobenzene(PXRD)	-	3.8514	6.5958	7.328	114.567	93.593	96.934	166.745
URACIL(RT)	$P2_1/b$	3.6552	10.3113	12.376	90	90	96.570	463.386
Uracil(PXRD)	-	3.6691	10.3146	12.3958	90	90	96.637	465.972
HXACAN35(RT)	$P2_1/n$	7.0661	9.3366	11.6508	90	97.410	90	762.223
Acetaminophen(PXRD)	-	7.1078	9.3986	11.7462	90	97.541	90	777.901
NIWFEE03(RT)	Cc	6.9531	15.0676	22.800	109.295	98.516	90	2226.607
Caffeine(PXRD)	-	6.9572	15.0839	22.8451	109.277	98.758	90	2233.362
DMANTL07(RT)	$P2_12_12_1$	5.549	8.694	16.902	90	90	90	815.403
D-Mannitol(PXRD)	-	5.5642	8.6848	16.9008	90	90	90	816.711
PROGST10(RT)	$P2_12_12_1$	10.340	12.559	13.798	90	90	90	1791.81
(+)-Progesterone(PXRD)	-	10.3741	12.6059	13.8464	90	90.268	90	1810.74

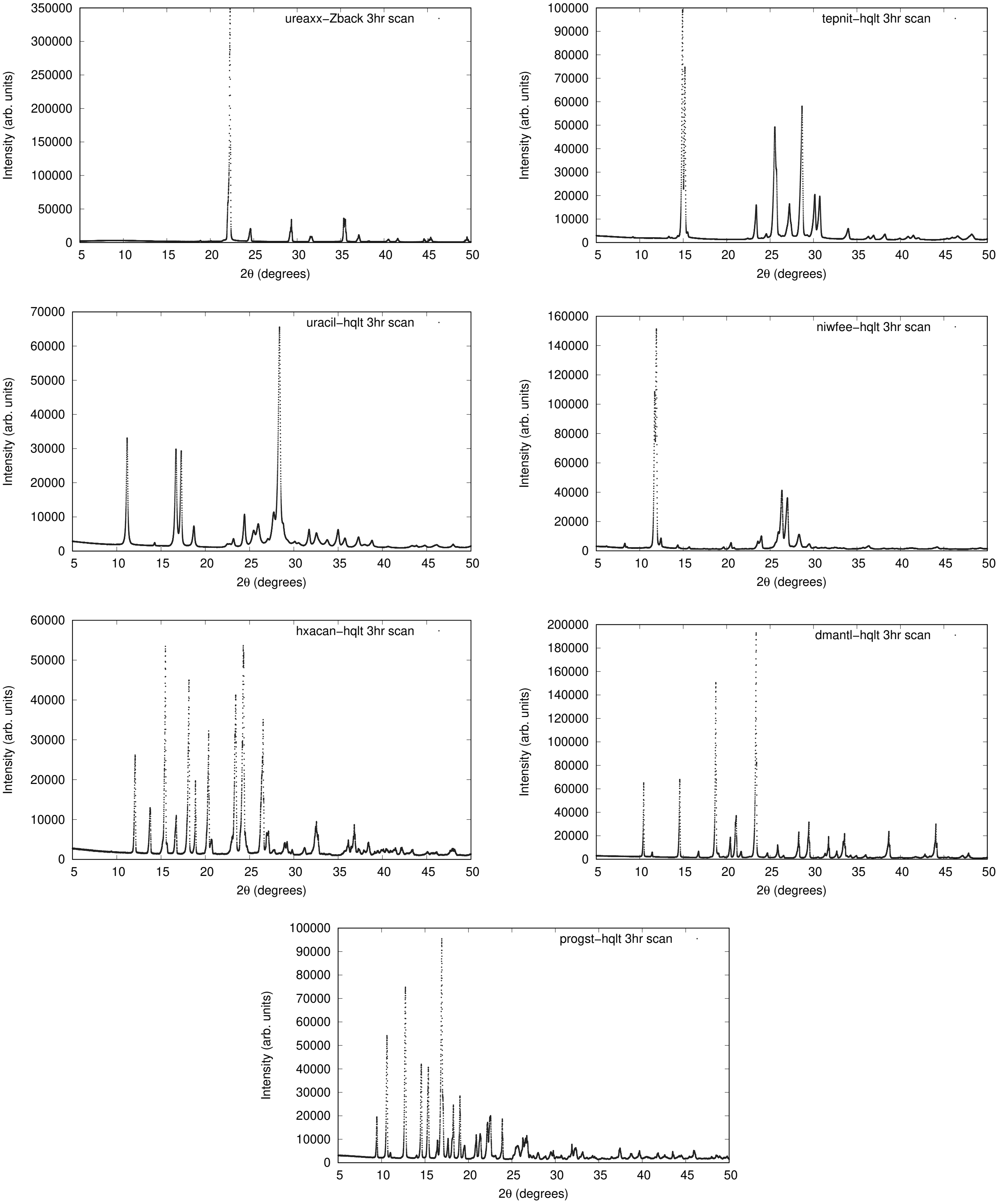

Figure S1: Experimental powder diffractograms collecting with the 3hr scan conditions.

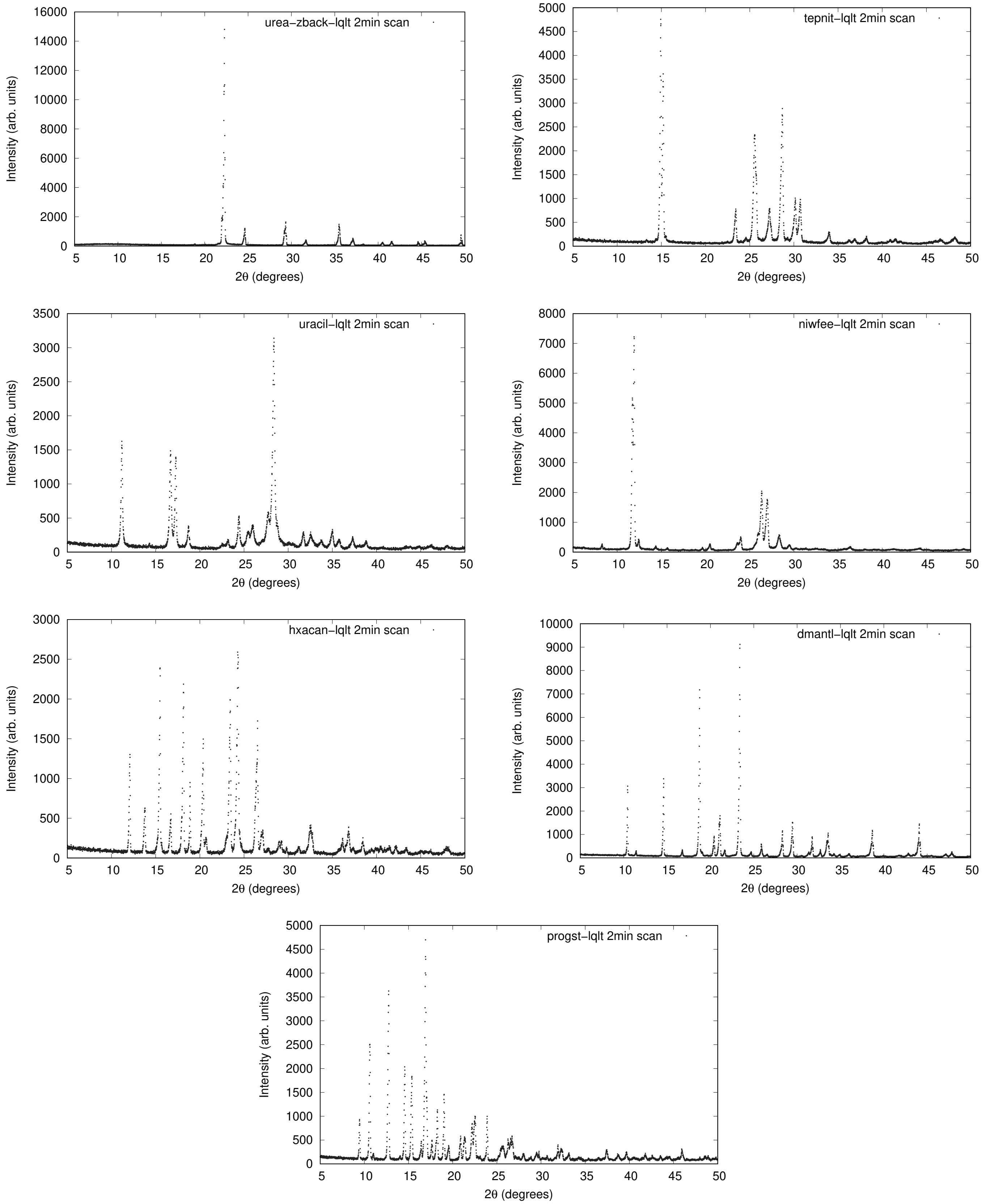

Figure S2: Experimental powder diffractograms collecting with the 2min scan conditions.

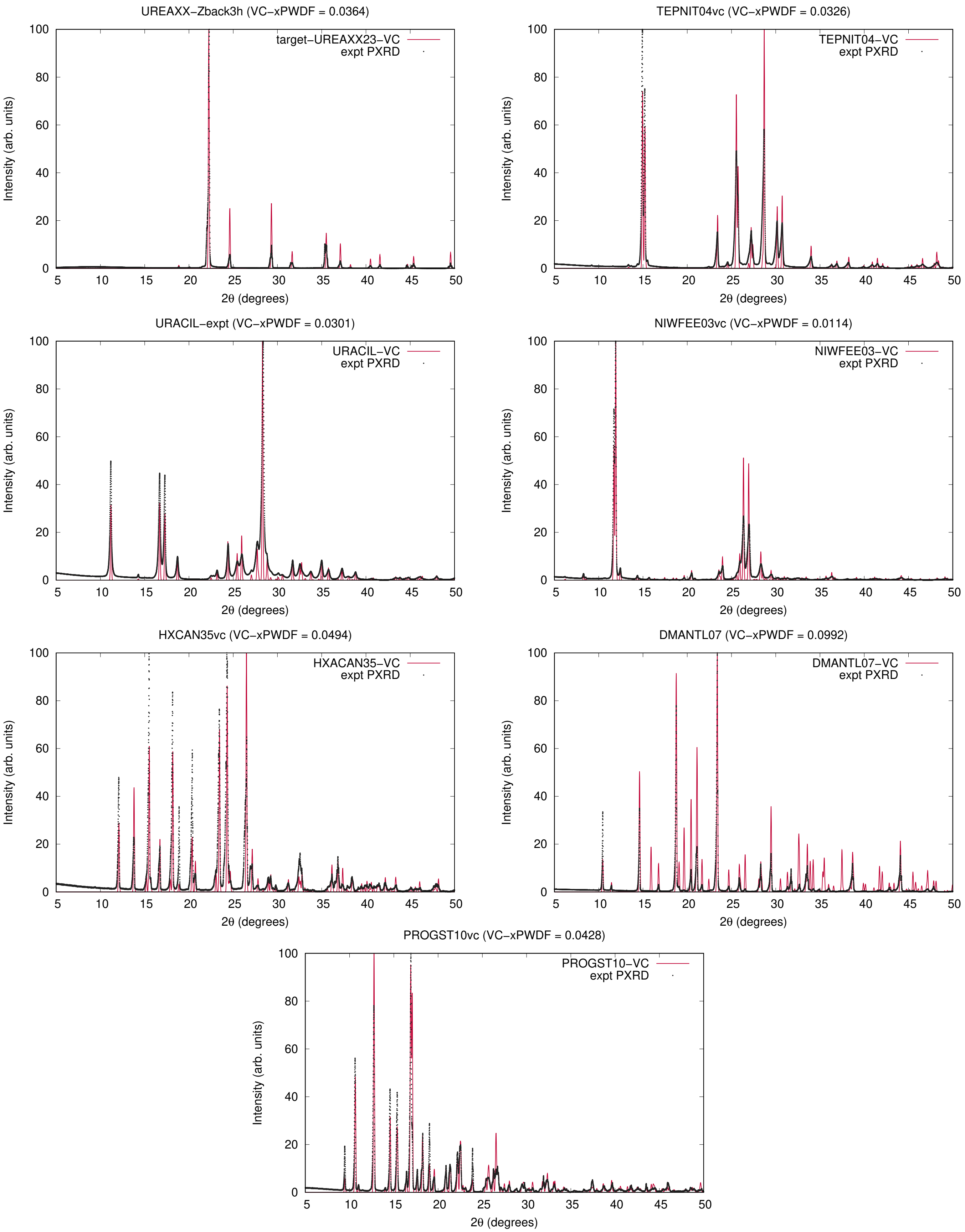

Figure S3: Overlays of the experimentally collected powder diffractograms with the simulated powder diffractogram of the matching polymorph from the CSD after running the VC-xPWDF protocol.

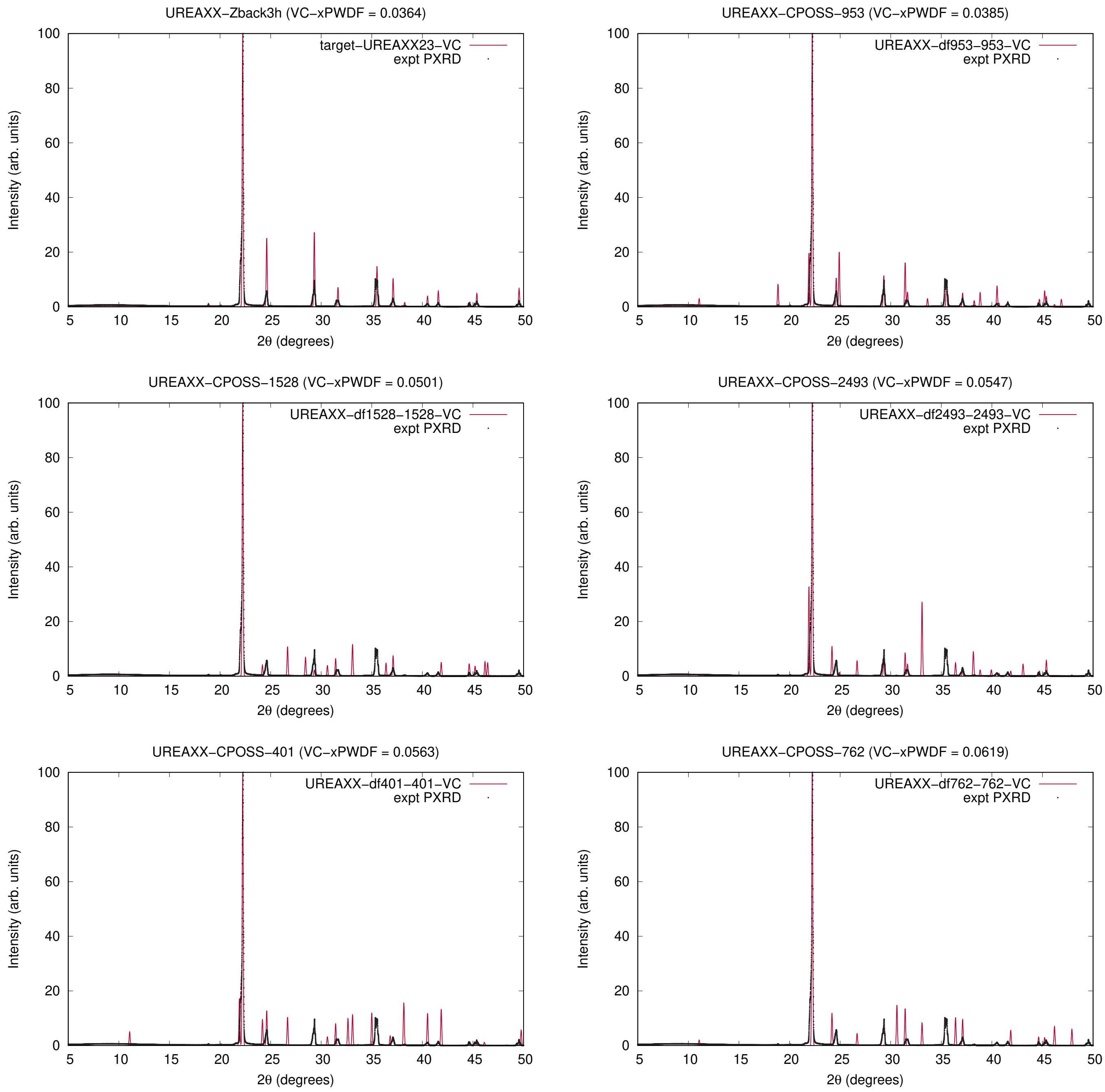

Figure S4: Overlay of simulated powder diffractograms of urea crystal structures after the VC-xPWDF protocol with the experimental powder diffractogram.

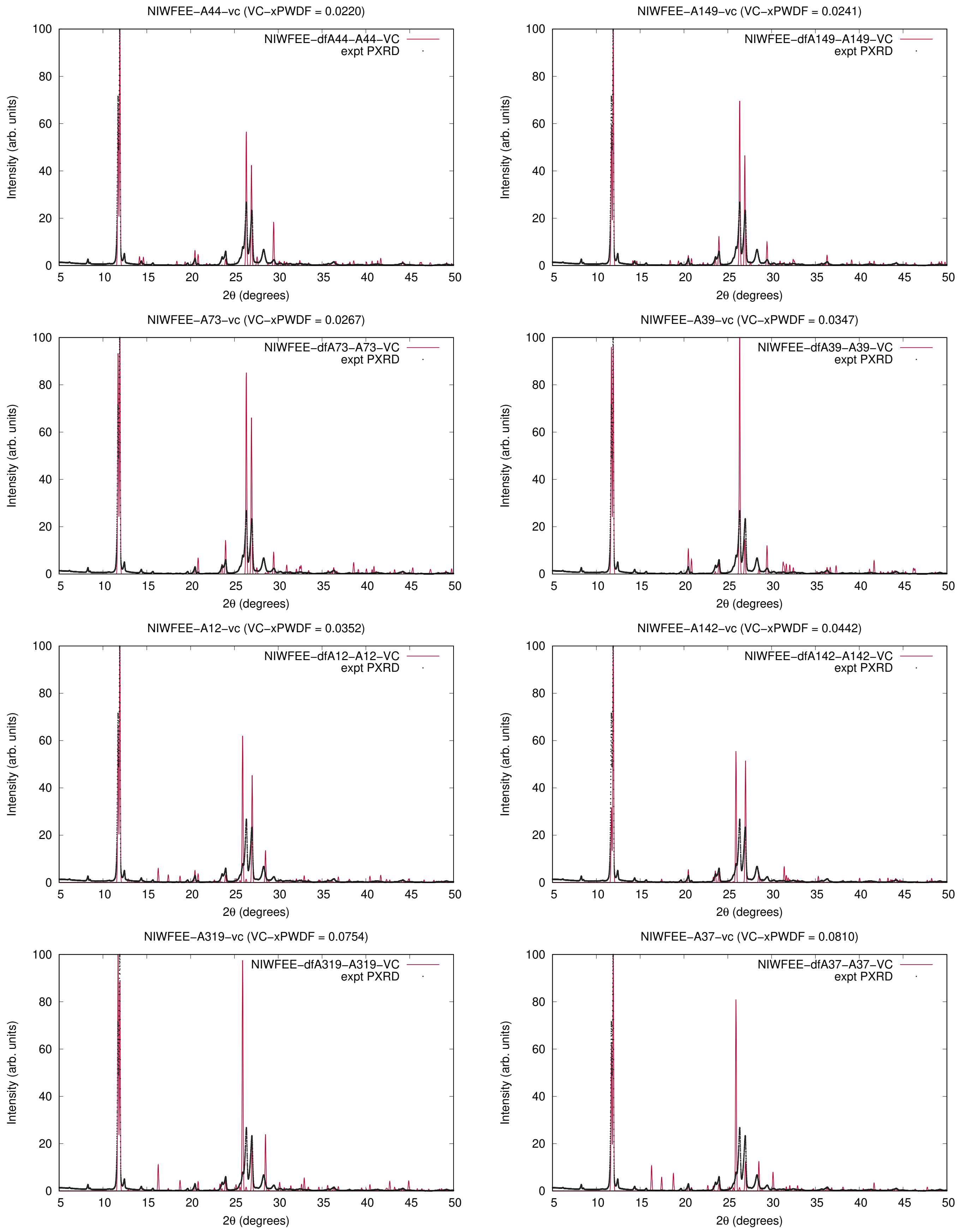

Figure S5: Overlay of simulated powder diffractograms of caffeine crystal structures after the VC-xPWDF protocol with the experimental powder diffractogram.

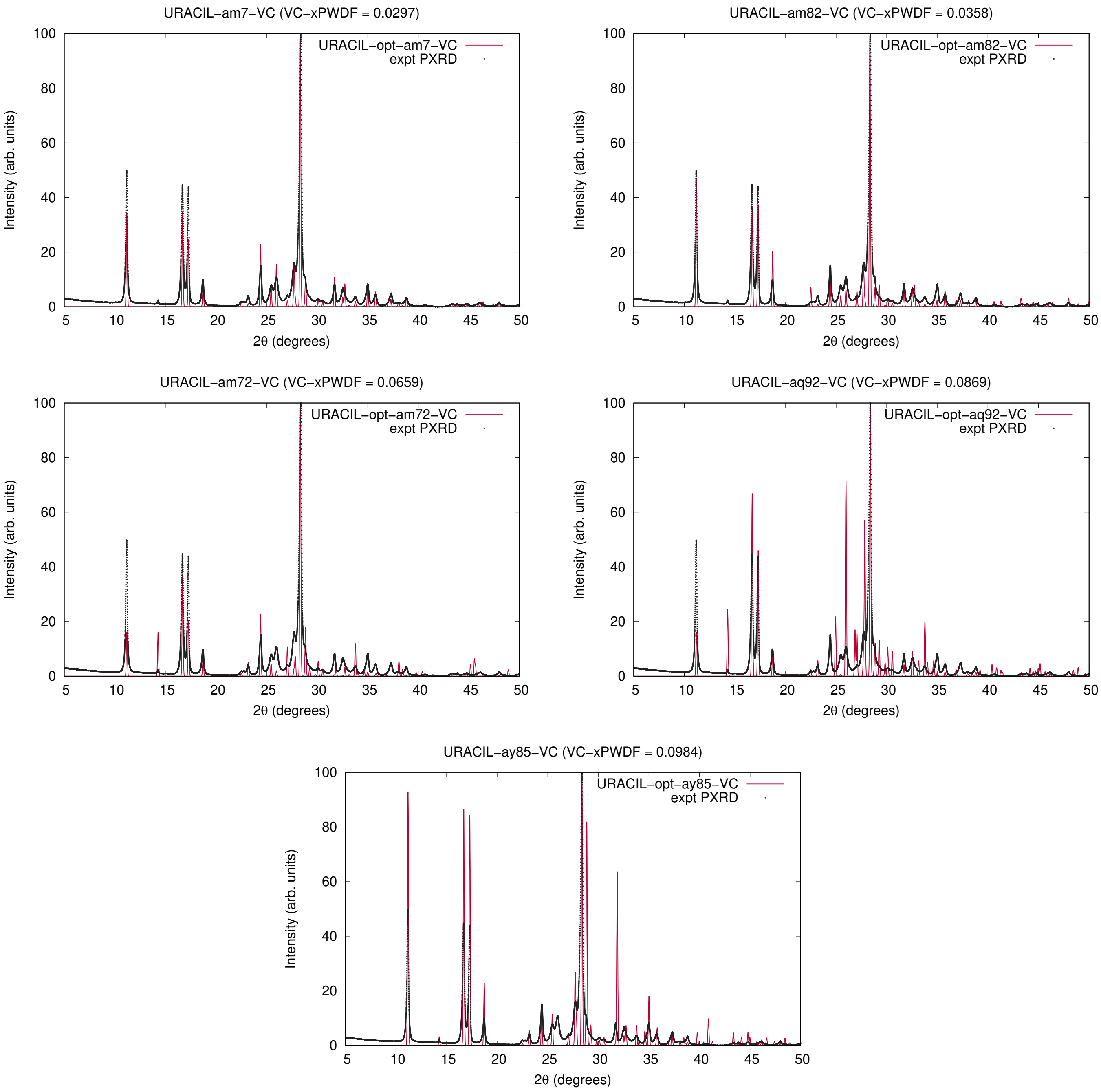

Figure S6: Overlay of simulated powder diffractograms of uracil crystal structures after the VC-xPWDF protocol with the experimental powder diffractogram.

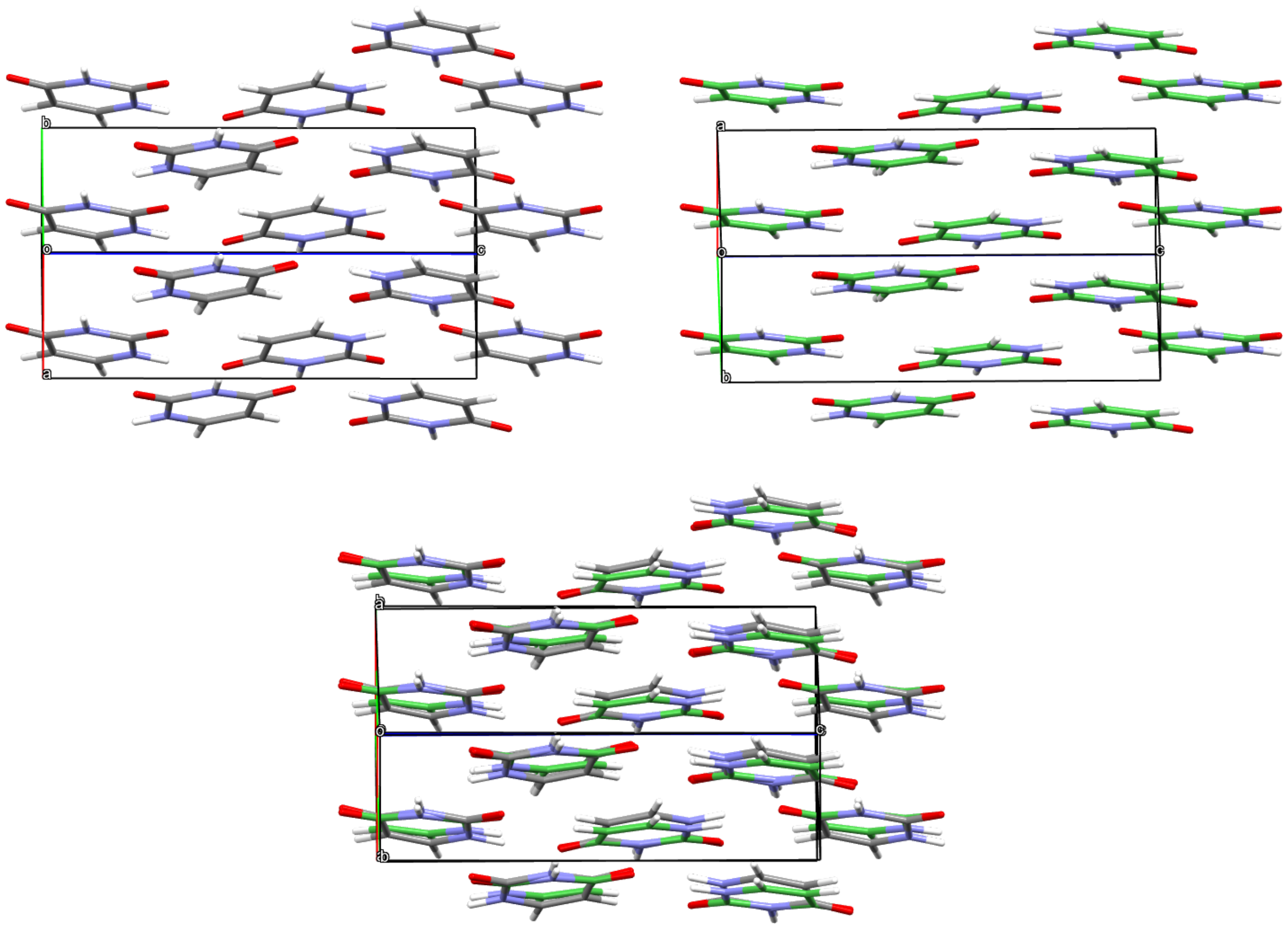

Figure S7: Images of the packing of two *in silico* generated crystal structures of uracil, am7 (top-left), and am82 (top-right), and an overlay of the two structures generated with the Crystal Packing Similarity tool's implementation of COMPACK[19] in the CCDC Mercury[20] software (V2022.2.0). The crystal structures used in these images had been modified with the VC-xPWDF protocol to match the experimental powder diffractogram.

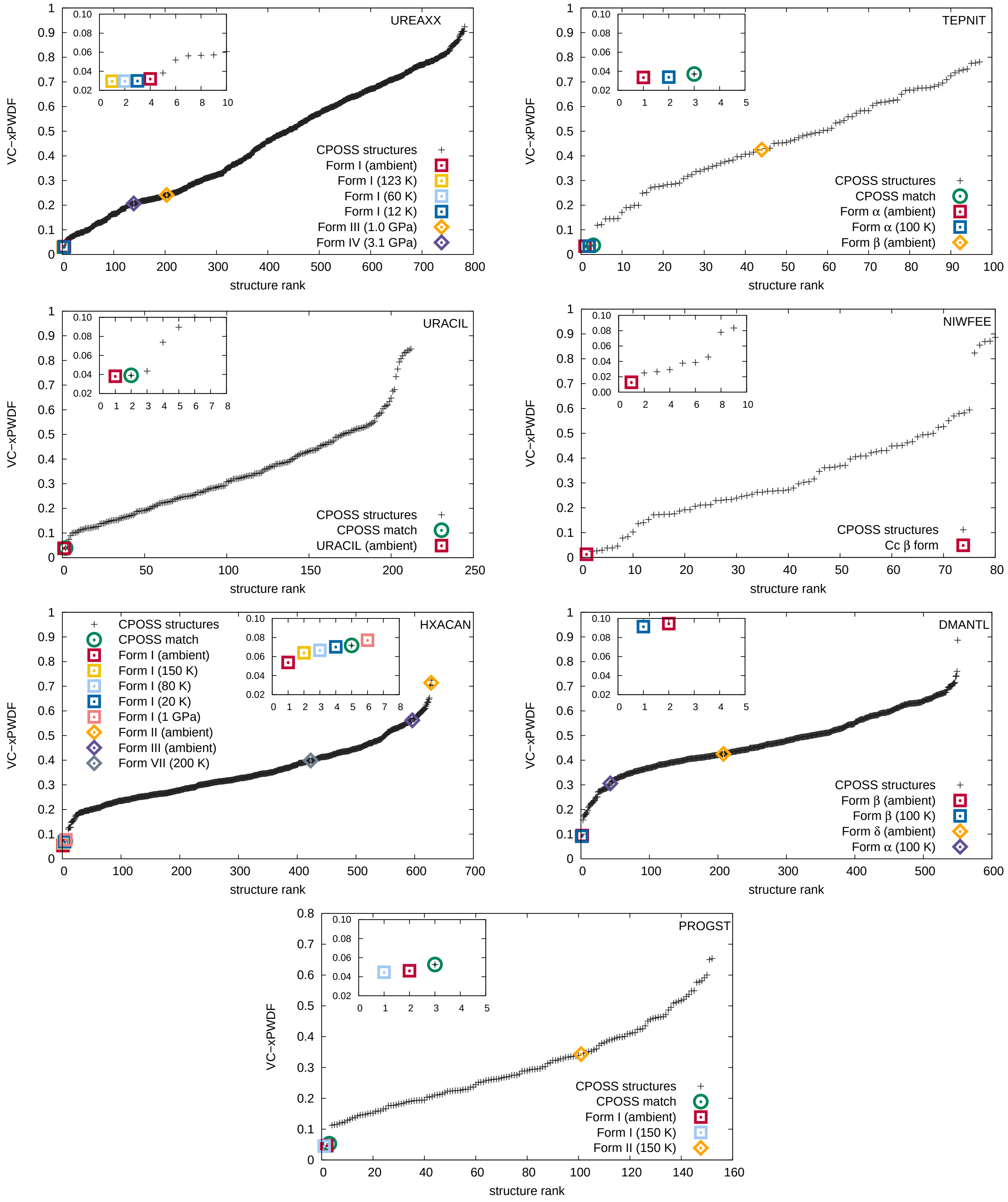

Figure S8: Plots showing the VC-xPWDF value from comparison of each input crystal structure to the experimental powder diffractogram collected from a 2 minute scan for that compound. The structures are ranked by lowest VC-xPWDF (most similar) and the insets provide views of the best matching structures (VC-xPWDF < 0.1). The point types indicate the source of each crystal structure: squares correspond to CSD structures of the matching polymorph, diamonds are CSD structures of different polymorphs, and + signs are CPOSS structures. If a matching structure is present in the CPOSS data, it is identified with a green circle.

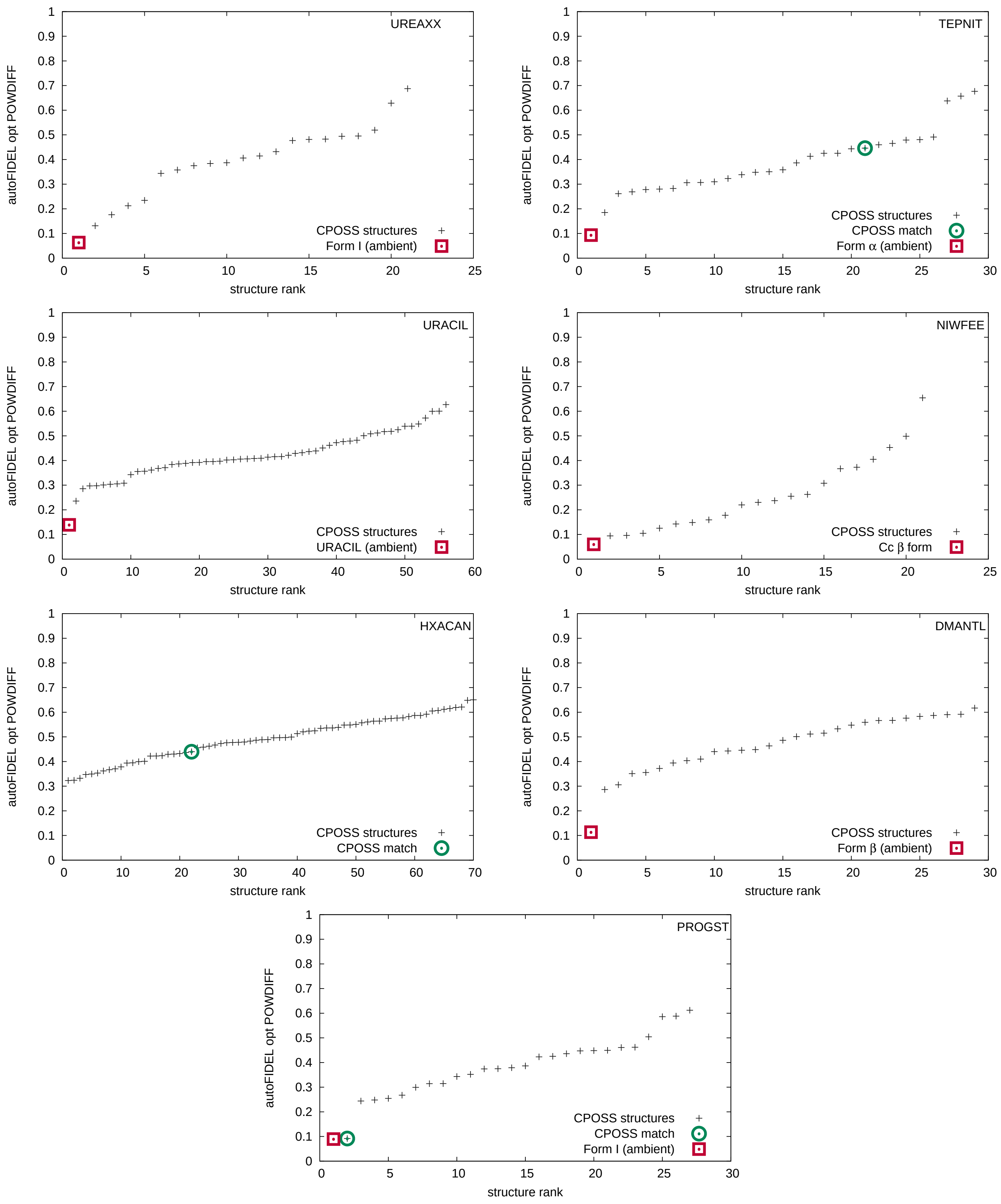

Figure S9: Plots showing the POWDIFF value after optimization with the autoFIDEL code between the crystal structure and the experimental powder diffractogram collected for that compound. The structures are ranked by smallest POWDIFF (most similar). The point types indicate the source of each crystal structure: squares correspond to CSD structures of the matching polymorph, diamonds are CSD structures of different polymorphs, and + signs are CPOSS structures. If a matching structure is present in the CPOSS data, it is identified with a green circle.

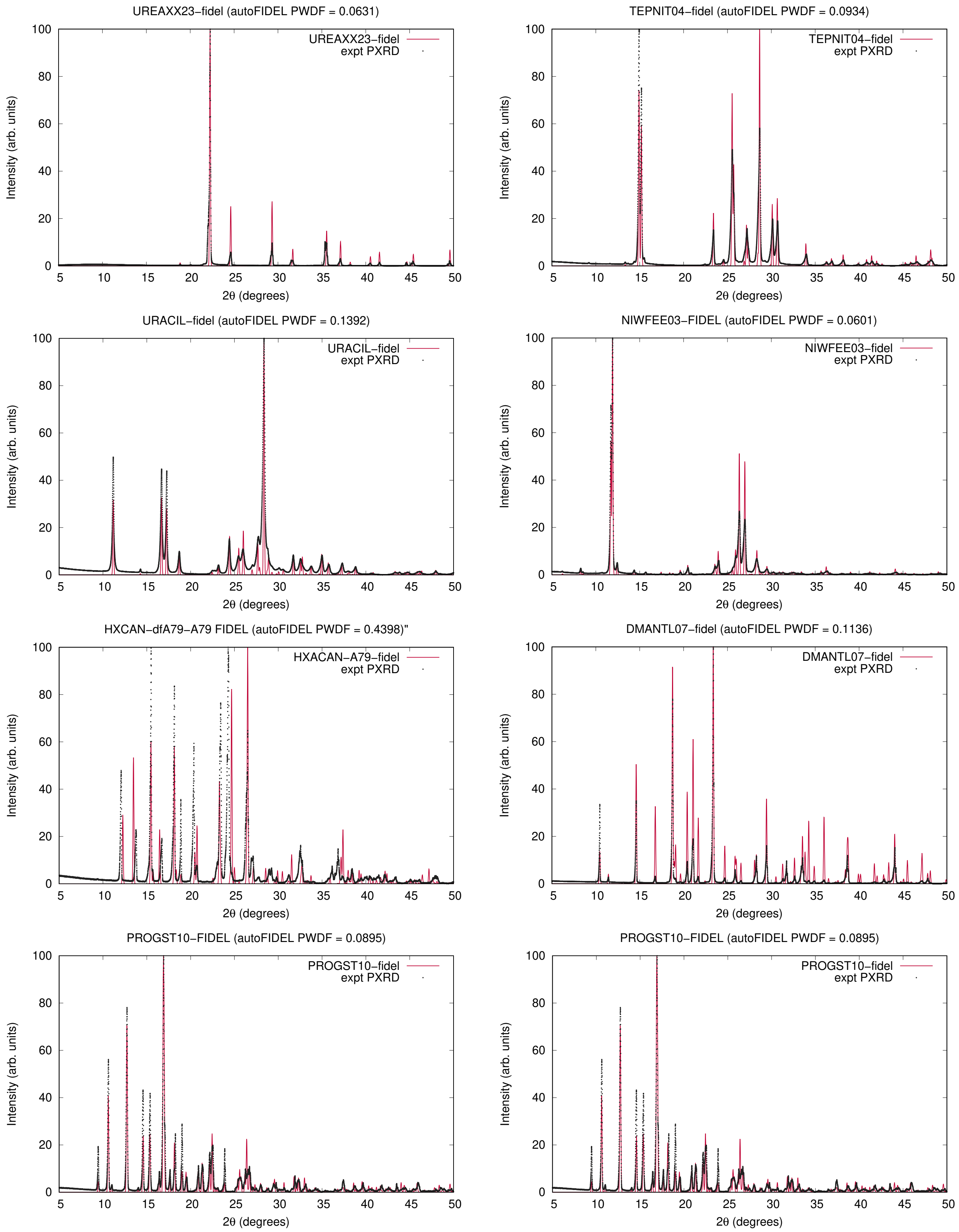

Figure S10: Overlay of simulated powder diffractograms from crystal structures after optimization with autoFIDEL with the experimental powder diffractogram.

S4.1 Rietveld refinement

S4.1.1 CSD structures

Rietveld refinement was performed on the matching CSD structures without modification and the results shown in Table S3. Clear correct alignment of the peaks was observed for all structures collected under ambient conditions, though the refinement values range from 20.10% < Rwp < 34.54% and 85.80 < χ^2 < 307.41.

Table S3: Summary of Rwp and χ^2 values from Rietveld refinement of the same polymorph CSD structures with the collected powder diffractograms. A success indicates that the peak positions were properly aligned between the two patterns, even if the intensities did not perfectly overlay. A poor refinement is indicated for cases where peak positions remained unaligned at the completion of the refinement.

CSD refcode	conditions	VC-xPWDF	Rwp (%)	χ^2	refinement
UREAXX23	RT	0.0364	30.06	178.49	success
UREAXX07	123 K	0.0335	41.69	343.32	poor
UREAXX11	60 K	0.0337	38.83	297.83	poor
UREAXX12	12 K	0.0339	39.95	315.26	poor
TEPNIT04	RT	0.0326	22.14	112.22	success
TEPNIT14	100 K	0.0330	26.65	162.59	poor
URACIL	RT	0.0290	20.10	85.80	success
NIWFEE03	RT	0.0114	26.08	154.23	success
HXACAN35	RT	0.0494	24.04	109.25	success
HXACAN04	150 K	0.0602	31.22	184.25	success
HXACAN15	80 K	0.0633	42.25	337.44	poor
HXACAN13	20 K	0.0670	32.73	202.51	poor
NIWFEE03	RT	0.0114	26.08	154.23	success
DMANTL07	RT	0.0992	33.90	197.86	success
DMANTL15	100 K	0.0962	36.61	256.66	success
PROGST10	RT	0.0428	34.54	307.41	success
PROGST12	150 K	0.0416	35.43	323.45	success

S4.1.2 CPOSS and VC-corrected structures

Table S4: Summary of Rwp and χ^2 values from Rietveld refinement of uracil structures with the collected powder diffractograms. A success indicates that the peak positions were properly aligned between the two patterns, even if the intensities did not perfectly overlay. A poor refinement is indicated for cases where peak positions remained unaligned at the completion of the refinement.

structure	rank	VC-xPWDF	ΔH (kJ/mol)	Rwp (%)	χ^2	refinement
URACIL-vc	1	0.0290	-	15.40	50.36	success
URACIL_opt_am7-vc	2	0.0297	0.0	17.43	64.52	success
URACIL_opt_am82-vc	3	0.0358	0.5	19.73	82.67	success
URACIL_opt_am72-vc	4	0.0659	2.0	38.62	316.74	poor
URACIL_opt_aq92-vc	5	0.0869	4.3	43.56	402.95	poor
URACIL_opt_ay85-vc	6	0.0984	7.6	21.78	100.74	poor

Table S5: Summary of Rwp and χ^2 values from Rietveld refinement of caffeine structures with the collected powder diffractograms. A success indicates that the peak positions were properly aligned between the two patterns, even if the intensities did not perfectly overlay. A poor refinement is indicated for cases where peak positions remained unaligned at the completion of the refinement.

structure	rank	VC-xPWDF	ΔH (kJ/mol)	Rwp (%)	χ^2	refinement
NIWFEE03-vc	1	0.0114	-	21.28	102.68	success
NIWFEE_dfA44_A44-vc	2	0.0220	7.0	32.45	238.78	poor
NIWFEE_dfA149_A149-vc	3	0.0241	6.2	33.88	260.28	poor
NIWFEE_dfA73_A73-vc	4	0.0267	0.0	32.46	238.92	poor
NIWFEE_dfA39_A39-vc	5	0.0347	10.7	25.77	150.59	poor
NIWFEE_dfA12_A12-vc	6	0.0352	8.1	32.18	234.82	poor
NIWFEE_dfA142_A142-vc	7	0.0442	3.1	25.76	150.47	poor
NIWFEE_dfA319_A319-vc	8	0.0754	10.8	33.60	256.00	poor
NIWFEE_dfA37_A37-vc	9	0.0810	10.1	37.27	314.98	poor

Table S6: Summary of Rwp and χ^2 values from Rietveld refinement of urea structures with the collected powder diffractograms. A success indicates that the peak positions were properly aligned between the two patterns, even if the intensities did not perfectly overlay. A poor refinement is indicated for cases where peak positions remained unaligned at the completion of the refinement.

structure	rank	VC-xPWDF	ΔH (kJ/mol)	Rwp (%)	χ^2	refinement
UREAXX23-vc	4	0.0364	-	20.26	81.08	success
UREAXX_df953_953	5	0.0385	14.9	40.07	317.16	poor
UREAXX_df1528_1528	6	0.0501	17.7	61.33	742.99	very poor
UREAXX_df2493_2493	7	0.0547	19.7	61.53	747.84	very poor
UREAXX_df401_401	8	0.0563	11.8	60.52	723.49	very poor
UREAXX_df762_762	9	0.0619	15.9	60.51	723.25	very poor

S4.2 Data tables

Table S7: Summary of data for urea. Relative enthalpy is given in kJ/mol, ranks are according to the next-right column, autoFIDEL comparisons were made with the default program parameters and the experimental powder diffractograms collected over 3 hrs.

structure	duplicates	ΔH	rank	VC-xPWDF (3h)	rank	VC-xPWDF (2m)	rank	autoFIDEL
UREAXX07	-	-	1	0.0335	1	0.0296	-	-
UREAXX11	-	-	2	0.0337	2	0.0297	-	-
UREAXX12	-	-	3	0.0339	3	0.0300	-	-
UREAXX23	-	-	4	0.0364	4	0.0320	1	0.0631
df953	0	19.9	5	0.0385	5	0.0382	-	-
df1528	0	22.8	6	0.0501	6	0.0519	-	-
df2493	0	24.7	7	0.0547	7	0.0562	-	-
df401	0	16.9	8	0.0563	8	0.0566	-	-
df762	0	21	9	0.0619	13	0.0631	-	-
df1994	0	28.3	10	0.0621	9	0.0572	-	-
df2382	0	15.9	11	0.0626	10	0.0607	-	-
df2004	0	26.4	12	0.0626	11	0.0619	-	-
df458	0	17.1	13	0.0640	12	0.0626	-	-
df626	0	19.1	14	0.0681	14	0.0677	-	-
df2152	0	27.7	15	0.0683	19	0.0728	-	-
df1551	0	20.1	16	0.0697	15	0.0678	-	-
df418	0	17.4	17	0.0741	20	0.0734	-	-
df937	0	18	18	0.0755	17	0.0701	-	-
df406	0	18.7	19	0.0758	21	0.0744	-	-
df99	0	15.3	20	0.0764	16	0.0689	-	-
df506	0	18.4	21	0.0765	18	0.0716	-	-
df340	0	17.2	22	0.0787	25	0.0804	-	-
df2065	0	25.2	23	0.0790	32	0.0847	-	-
df268	0	16.5	24	0.0822	23	0.0761	-	-
df859	0	21.1	25	0.0828	24	0.0768	-	-
df894	0	16.9	26	0.0829	22	0.0761	-	-
df2016	0	24.3	27	0.0855	26	0.0809	-	-
df938	0	21.4	28	0.0858	35	0.0873	-	-
df1817	0	14.9	29	0.0876	27	0.0821	-	-
df2494	0	23.1	30	0.0882	31	0.0847	-	-
df685	0	17.6	31	0.0890	33	0.0853	-	-
df139	0	16.5	32	0.0898	39	0.0899	-	-
df1127	0	16.5	33	0.0901	28	0.0831	-	-
df900	0	13.8	34	0.0915	34	0.0868	-	-
df1816	2	5	35	0.0926	30	0.0847	-	-
df1577	0	14.2	36	0.0927	38	0.0898	-	-
df1051	0	15.7	37	0.0934	29	0.0846	-	-
df945	0	19.3	38	0.0954	45	0.0975	-	-
df1394	0	14.2	39	0.0955	37	0.0897	-	-

continued...

structure	duplicates	ΔH	rank	VC-xPWDF (3h)	rank	VC-xPWDF (2m)	rank	autoFIDEL
df186	0	18	40	0.0955	49	0.0983	-	-
df1020	0	15	41	0.0959	48	0.0982	-	-
df2264	0	14.1	42	0.0960	43	0.0945	-	-
df154	0	16.7	43	0.0972	42	0.0922	-	-
df790	0	19.7	44	0.0977	41	0.0908	-	-
df1036	0	17.7	45	0.0981	40	0.0899	-	-
df2246	0	31.5	46	0.1000	36	0.0892	-	-
df1422	0	22.3	47	0.1026	52	0.1011	-	-
df1503	0	23.8	48	0.1035	47	0.0977	-	-
df1095	0	19.4	49	0.1037	46	0.0975	-	-
df1541	0	9.3	50	0.1058	50	0.0997	-	-
df1576	0	20.1	51	0.1073	55	0.1061	4	0.2124
df1864	0	27.9	52	0.1078	51	0.1006	-	-
df649	0	20.5	53	0.1089	53	0.1020	-	-
df351	0	14.2	54	0.1092	57	0.1123	-	-
df2168	0	15.2	55	0.1094	54	0.1042	-	-
df628	0	9.5	56	0.1111	56	0.1071	-	-
df552	0	19.6	57	0.1117	58	0.1131	-	-
df895	0	17.9	58	0.1122	59	0.1131	-	-
df1453	0	21.8	59	0.1124	44	0.0948	-	-
df639	0	17.1	60	0.1152	61	0.1159	-	-
df744	0	17.7	61	0.1203	62	0.1170	-	-
df1116	0	14	62	0.1220	60	0.1151	-	-
df1564	0	17.4	63	0.1258	67	0.1251	-	-
df319	0	14	64	0.1258	65	0.1235	-	-
df449	0	7.7	65	0.1279	68	0.1265	-	-
df2053	0	15.6	66	0.1284	63	0.1199	-	-
df526	0	7.5	67	0.1295	70	0.1279	-	-
df532	0	7.5	68	0.1297	72	0.1281	-	-
df622	0	18.7	69	0.1309	73	0.1287	-	-
df2273	0	24.2	70	0.1317	71	0.1280	-	-
df1466	0	15.8	71	0.1318	66	0.1236	-	-
df2079	0	17	72	0.1328	77	0.1327	-	-
df1216	0	17.6	73	0.1334	64	0.1214	-	-
df1360	0	19.5	74	0.1338	80	0.1358	-	-
df843	0	19.1	75	0.1361	74	0.1298	-	-
df2096	0	22.7	76	0.1384	75	0.1300	-	-
df1840	0	5.3	77	0.1397	69	0.1277	-	-
df745	0	17.9	78	0.1411	82	0.1379	-	-
df2068	0	23.9	79	0.1419	79	0.1344	-	-
df617	0	17.2	80	0.1457	87	0.1424	5	0.2346
df1188	0	17.7	81	0.1469	83	0.1392	-	-
df621	0	19.1	82	0.1475	76	0.1312	-	-
df1805	0	25.6	83	0.1478	81	0.1376	-	-

continued…

structure	duplicates	ΔH	rank	VC-xPWDF (3h)	rank	VC-xPWDF (2m)	rank	autoFIDEL
df1823	0	19.2	84	0.1516	86	0.1408	-	-
df2147	0	1.2	85	0.1524	84	0.1396	-	-
df1081	0	16.9	86	0.1527	85	0.1407	-	-
df1790	0	23.3	87	0.1528	88	0.1466	-	-
df1023	1	8.5	88	0.1537	78	0.1333	-	-
df502	0	12.8	89	0.1616	89	0.1530	-	-
df2399	0	13.8	90	0.1619	93	0.1575	-	-
df2092	0	17.4	91	0.1624	90	0.1542	-	-
df2034	0	24.3	92	0.1630	94	0.1580	-	-
df1850	0	19.7	93	0.1644	102	0.1652	-	-
df671	0	15.7	94	0.1646	105	0.1660	-	-
df1867	0	10.3	95	0.1648	98	0.1633	-	-
df1343	0	20.3	96	0.1653	91	0.1544	-	-
df2002	0	24.7	97	0.1673	95	0.1608	-	-
df2122	0	23.2	98	0.1677	92	0.1546	-	-
df1932	0	22.8	99	0.1697	106	0.1662	-	-
df689	0	17.2	100	0.1712	96	0.1629	-	-
df1776	0	17.1	101	0.1728	110	0.1734	-	-
df990	0	17.4	102	0.1731	107	0.1686	-	-
df1549	1	10.4	103	0.1734	104	0.1657	-	-
df1858	0	18.1	104	0.1743	103	0.1654	-	-
df932	0	25	105	0.1772	99	0.1640	-	-
df501	0	12	106	0.1776	109	0.1723	-	-
df1363	0	18.4	107	0.1792	108	0.1721	-	-
df1009	0	20.3	108	0.1796	97	0.1633	-	-
df1287	0	18.4	109	0.1810	111	0.1740	-	-
df1426	0	19.8	110	0.1824	100	0.1645	-	-
df2027	0	14	111	0.1837	117	0.1847	-	-
df667	0	17.5	112	0.1856	116	0.1844	-	-
df943	0	14.4	113	0.1858	118	0.1848	-	-
df240	0	14.6	114	0.1868	101	0.1647	-	-
df447	0	12.9	115	0.1877	115	0.1839	-	-
df2452	0	14.1	116	0.1878	112	0.1785	-	-
df887	0	14.6	117	0.1902	122	0.1876	-	-
df2597	0	18.7	118	0.1910	121	0.1870	-	-
df676	0	15.4	119	0.1967	114	0.1807	-	-
df485	0	12.6	120	0.1990	120	0.1863	-	-
df420	0	13.9	121	0.2003	131	0.2003	-	-
df341	0	10.4	122	0.2007	119	0.1853	-	-
df207	0	5.4	123	0.2020	113	0.1803	-	-
df2321	1	10.3	124	0.2033	123	0.1881	-	-
df204	0	14.5	125	0.2044	133	0.2008	-	-
df136	0	11.9	126	0.2051	136	0.2042	-	-
df1152	0	21.9	127	0.2051	124	0.1913	-	-

continued…

structure	duplicates	ΔH	rank	VC-xPWDF (3h)	rank	VC-xPWDF (2m)	rank	autoFIDEL
df172	0	8.9	128	0.2064	140	0.2073	-	-
df302	0	9.4	129	0.2071	138	0.2066	-	-
UREAXX26	-	-	130	0.2087	139	0.2070	-	-
df143	0	16.1	131	0.2099	128	0.1998	-	-
df390	0	13.5	132	0.2103	132	0.2006	-	-
df2105	0	17.2	133	0.2113	135	0.2037	-	-
df630	0	18.4	134	0.2115	158	0.2179	-	-
df217	0	13.6	135	0.2116	144	0.2087	20	0.6286
df1113	0	22	136	0.2125	137	0.2062	-	-
df1329	0	21.6	137	0.2126	150	0.2132	-	-
df318	1	13.2	138	0.2130	146	0.2103	-	-
df1073	0	13.3	139	0.2134	126	0.1970	-	-
df917	0	21.1	140	0.2138	125	0.1962	-	-
df682	2	0.9	141	0.2148	129	0.2000	-	-
df2012	0	11	142	0.2152	127	0.1995	-	-
df2156	0	18.5	143	0.2155	143	0.2084	-	-
df1512	0	16.5	144	0.2159	130	0.2000	-	-
df81	0	10.6	145	0.2176	142	0.2081	-	-
df315	0	10	146	0.2189	147	0.2106	-	-
df2374	0	24.4	147	0.2190	179	0.2259	-	-
df732	0	15.5	148	0.2196	153	0.2156	-	-
df1291	0	20.5	149	0.2200	134	0.2010	-	-
df647	0	18.5	150	0.2208	170	0.2225	-	-
df781	0	18.1	151	0.2210	152	0.2140	-	-
df2141	0	18.5	152	0.2211	169	0.2224	-	-
df795	0	19.4	153	0.2213	155	0.2175	-	-
df832	0	14.9	154	0.2235	168	0.2220	-	-
df1996	0	11.5	155	0.2238	148	0.2113	-	-
df1813	0	19.1	156	0.2243	154	0.2162	-	-
df1256	0	15.7	157	0.2248	141	0.2078	-	-
df1833	0	14.1	158	0.2259	151	0.2137	-	-
df489	0	20	159	0.2274	176	0.2252	-	-
df1235	0	14.4	160	0.2281	175	0.2250	-	-
df705	0	18.7	161	0.2286	178	0.2255	-	-
df805	0	16	162	0.2288	156	0.2176	-	-
df1132	0	10.4	163	0.2289	145	0.2093	-	-
df1441	0	18.1	164	0.2302	160	0.2184	-	-
df784	0	18	165	0.2304	167	0.2216	-	-
df2102	0	13.3	166	0.2308	159	0.2179	-	-
df1428	0	6	167	0.2312	163	0.2192	-	-
df666	0	15.3	168	0.2314	186	0.2300	-	-
df1243	0	23.8	169	0.2316	162	0.2188	19	0.5192
df1435	0	19.8	170	0.2318	165	0.2206	-	-
df102	0	11.8	171	0.2320	173	0.2238	-	-

continued...

structure	duplicates	ΔH	rank	VC-xPWDF (3h)	rank	VC-xPWDF (2m)	rank	autoFIDEL
df297	0	10.8	172	0.2322	172	0.2235	-	-
df403	0	14.1	173	0.2328	184	0.2296	-	-
df1540	0	2.6	174	0.2336	149	0.2114	-	-
df325	0	5.5	175	0.2338	180	0.2270	-	-
df888	0	20.2	176	0.2339	185	0.2297	-	-
df964	0	12.1	177	0.2346	183	0.2293	-	-
df2403	0	4.2	178	0.2354	164	0.2199	-	-
df472	0	7.2	179	0.2356	192	0.2329	-	-
df223	0	14.4	180	0.2360	187	0.2304	-	-
df1138	0	15	181	0.2364	166	0.2214	-	-
df1999	0	24.4	182	0.2370	181	0.2280	-	-
df1968	0	19.1	183	0.2376	188	0.2307	-	-
df1455	0	18.7	184	0.2385	174	0.2245	-	-
df1562	0	21.1	185	0.2388	161	0.2186	-	-
df381	0	11.1	186	0.2405	182	0.2285	-	-
df2404	2	10.9	187	0.2407	157	0.2178	-	-
df1340	0	11	188	0.2408	171	0.2231	-	-
df1332	0	20.9	189	0.2411	193	0.2332	-	-
df61	0	12	190	0.2414	191	0.2327	-	-
df2420	0	22.2	191	0.2429	219	0.2503	-	-
df1780	0	25.8	192	0.2432	194	0.2359	-	-
df177	0	9.5	193	0.2442	195	0.2376	-	-
df2409	0	11	194	0.2445	177	0.2255	-	-
df2680	0	21.4	195	0.2446	201	0.2396	-	-
df122	0	7	196	0.2476	189	0.2310	-	-
df1506	0	20.9	197	0.2481	200	0.2395	-	-
df117	0	11.5	198	0.2511	198	0.2387	-	-
df854	0	13.7	199	0.2515	206	0.2428	-	-
df668	0	18.4	200	0.2523	196	0.2377	-	-
UREAXX33	-	-	201	0.2528	203	0.2414	-	-
df2145	0	0.8	202	0.2530	197	0.2387	-	-
df1504	0	10	203	0.2539	190	0.2322	-	-
df2363	0	1.2	204	0.2552	208	0.2439	-	-
df1104	0	22.3	205	0.2552	225	0.2540	-	-
df1202	0	21.2	206	0.2560	199	0.2388	-	-
df1960	0	2.6	207	0.2568	202	0.2398	-	-
df946	0	16.8	208	0.2569	220	0.2506	-	-
df1894	0	24.5	209	0.2570	204	0.2416	-	-
df1160	0	12.9	210	0.2573	205	0.2419	-	-
df1328	0	10.1	211	0.2576	209	0.2439	-	-
df2190	0	20.2	212	0.2578	207	0.2433	-	-
df1346	0	20.2	213	0.2591	211	0.2449	-	-
df1361	0	21.3	214	0.2597	212	0.2453	-	-
df374	0	0	215	0.2609	214	0.2473	-	-

continued…

structure	duplicates	ΔH	rank	VC-xPWDF (3h)	rank	VC-xPWDF (2m)	rank	autoFIDEL
df1922	0	13.8	216	0.2615	222	0.2528	-	-
df1072	0	23.6	217	0.2619	215	0.2484	-	-
df2591	0	11.8	218	0.2622	210	0.2440	-	-
df245	0	11.2	219	0.2627	217	0.2499	-	-
df417	0	12.7	220	0.2630	229	0.2581	-	-
df2075	0	17.7	221	0.2650	218	0.2501	-	-
df567	0	16.6	222	0.2650	213	0.2466	-	-
df2235	0	14.8	223	0.2653	228	0.2579	-	-
df1070	0	19.1	224	0.2694	221	0.2518	-	-
df2325	0	9.6	225	0.2699	230	0.2585	-	-
df1220	0	19.9	226	0.2725	216	0.2485	-	-
df1768	0	23.4	227	0.2729	227	0.2574	-	-
df2295	0	21.8	228	0.2738	226	0.2545	-	-
df103	0	12.4	229	0.2742	238	0.2680	-	-
df200	0	16.7	230	0.2750	232	0.2611	-	-
df577	0	10.4	231	0.2763	223	0.2532	-	-
df821	0	16	232	0.2767	224	0.2539	-	-
df1873	0	5.7	233	0.2773	233	0.2621	-	-
df1936	0	22.7	234	0.2783	235	0.2655	-	-
df242	0	0	235	0.2783	231	0.2608	-	-
df2391	0	20	236	0.2798	236	0.2659	-	-
df1144	0	15.5	237	0.2799	241	0.2699	-	-
df1929	0	14.2	238	0.2805	243	0.2720	-	-
df1611	0	21	239	0.2839	237	0.2667	-	-
df550	0	17.4	240	0.2843	240	0.2693	-	-
df1189	0	10.6	241	0.2846	234	0.2639	-	-
df2222	0	12.2	242	0.2847	239	0.2688	-	-
df218	0	10.6	243	0.2888	244	0.2760	-	-
df1049	0	21.6	244	0.2900	245	0.2767	11	0.4062
df2489	0	25.3	245	0.2903	247	0.2798	-	-
df205	0	10.5	246	0.2909	242	0.2706	-	-
df2449	0	9.6	247	0.2913	250	0.2812	-	-
df518	0	12.9	248	0.2919	246	0.2794	-	-
df2069	0	19.9	249	0.2940	256	0.2867	-	-
df1554	0	18.4	250	0.2958	252	0.2842	-	-
df145	0	16	251	0.2968	248	0.2799	-	-
df925	0	9.6	252	0.2976	249	0.2806	-	-
df16	0	7.3	253	0.3011	251	0.2837	-	-
df1437	0	22.5	254	0.3019	257	0.2874	-	-
df609	0	9.1	255	0.3020	261	0.2933	-	-
df965	0	12.2	256	0.3022	255	0.2863	-	-
df492	0	18.1	257	0.3029	263	0.2940	-	-
df1245	0	15.9	258	0.3057	269	0.2990	-	-
df1573	0	5.9	259	0.3066	260	0.2923	-	-

continued...

structure	duplicates	ΔH	rank	VC-xPWDF (3h)	rank	VC-xPWDF (2m)	rank	autoFIDEL
df638	0	7.9	260	0.3072	258	0.2903	-	-
df1725	0	22.5	261	0.3076	254	0.2862	-	-
df690	0	9.6	262	0.3087	264	0.2945	-	-
df2123	0	27.7	263	0.3091	253	0.2849	-	-
df631	0	16.1	264	0.3091	262	0.2936	-	-
df2083	0	12.1	265	0.3098	265	0.2956	-	-
df1857	0	21.4	266	0.3105	274	0.3042	-	-
df98	0	6.6	267	0.3114	271	0.3002	-	-
df2173	0	20.1	268	0.3122	259	0.2923	-	-
df123	0	11.2	269	0.3122	270	0.2999	-	-
df1880	0	22.8	270	0.3135	266	0.2956	-	-
df436	0	14.9	271	0.3168	277	0.3068	-	-
df1556	0	15.5	272	0.3174	267	0.2961	-	-
df2620	0	21.4	273	0.3179	273	0.3018	-	-
df1981	0	23.5	274	0.3184	283	0.3108	-	-
df1593	0	19.5	275	0.3201	268	0.2976	-	-
df261	0	11.3	276	0.3202	281	0.3100	18	0.4955
df104	0	4.6	277	0.3215	279	0.3084	-	-
df174	0	14.7	278	0.3224	276	0.3066	-	-
df1861	0	21.7	279	0.3241	290	0.3156	-	-
df2423	0	19.4	280	0.3247	275	0.3064	-	-
df2425	0	23.9	281	0.3248	278	0.3082	-	-
df1325	0	5.3	282	0.3262	289	0.3152	-	-
df51	0	10.8	283	0.3262	272	0.3013	-	-
df885	0	20.8	284	0.3263	288	0.3150	-	-
df278	0	17.6	285	0.3289	286	0.3125	-	-
df670	0	13.3	286	0.3291	292	0.3169	-	-
df63	0	6.3	287	0.3291	282	0.3101	-	-
df1244	0	11	288	0.3302	296	0.3191	-	-
df994	0	14	289	0.3322	287	0.3126	16	0.4830
df2682	0	18.3	290	0.3327	306	0.3269	-	-
df837	0	9.5	291	0.3333	285	0.3123	-	-
df596	0	19.5	292	0.3338	280	0.3099	9	0.3839
df2015	0	1.8	293	0.3350	297	0.3201	-	-
df653	0	15.8	294	0.3353	291	0.3168	-	-
df568	0	11.3	295	0.3357	311	0.3290	-	-
df804	0	1.6	296	0.3360	302	0.3242	-	-
df718	0	14.6	297	0.3372	298	0.3203	-	-
df2384	0	26.6	298	0.3386	314	0.3315	-	-
df849	0	0.5	299	0.3391	295	0.3189	-	-
df844	0	18	300	0.3392	299	0.3219	-	-
df1746	0	15.3	301	0.3393	284	0.3121	-	-
df909	0	20	302	0.3410	293	0.3173	-	-
df1165	0	14.8	303	0.3417	301	0.3233	-	-

continued...

structure	duplicates	ΔH	rank	VC-xPWDF (3h)	rank	VC-xPWDF (2m)	rank	autoFIDEL
df1473	0	20.6	304	0.3421	294	0.3184	-	-
df1892	0	27.4	305	0.3429	300	0.3223	-	-
df2043	0	12.3	306	0.3431	303	0.3250	-	-
df1948	0	24.6	307	0.3434	315	0.3373	-	-
df221	0	8.3	308	0.3435	305	0.3264	-	-
df1796	0	17.3	309	0.3437	309	0.3282	-	-
df2379	0	27.6	310	0.3439	307	0.3275	-	-
df787	0	15.1	311	0.3441	304	0.3259	-	-
df1904	0	21.7	312	0.3459	312	0.3298	-	-
df2407	1	12	313	0.3459	310	0.3283	-	-
df482	0	18.3	314	0.3483	317	0.3433	-	-
df650	0	5	315	0.3538	313	0.3300	-	-
df147	0	6.9	316	0.3543	308	0.3276	-	-
df531	0	15.8	317	0.3646	319	0.3457	-	-
df708	0	18.6	318	0.3675	321	0.3493	-	-
df508	0	16.6	319	0.3675	318	0.3446	-	-
df290	0	11.8	320	0.3681	323	0.3521	-	-
df652	0	12	321	0.3687	325	0.3528	-	-
df717	0	19.5	322	0.3701	326	0.3532	-	-
df1536	0	17.3	323	0.3707	320	0.3491	-	-
df2590	0	11.8	324	0.3716	316	0.3429	-	-
df657	0	13.4	325	0.3722	329	0.3609	-	-
df495	0	16.4	326	0.3770	341	0.3718	-	-
df373	0	16.3	327	0.3779	322	0.3513	-	-
df2036	0	13.2	328	0.3789	328	0.3566	15	0.4815
df1618	0	22.9	329	0.3807	324	0.3524	-	-
df2319	0	18.8	330	0.3808	342	0.3729	-	-
df1527	0	5.3	331	0.3809	332	0.3648	-	-
df1249	0	18.8	332	0.3810	330	0.3629	-	-
df2405	0	5.9	333	0.3826	333	0.3651	-	-
df49	0	7.6	334	0.3830	331	0.3634	-	-
df284	0	13.3	335	0.3848	327	0.3549	-	-
df1568	0	10.3	336	0.3848	340	0.3706	-	-
df300	0	16.1	337	0.3864	337	0.3686	-	-
df1017	0	16	338	0.3864	338	0.3699	-	-
df273	0	2.8	339	0.3867	339	0.3700	-	-
df2454	0	29.5	340	0.3872	334	0.3652	-	-
df115	0	6.8	341	0.3874	335	0.3660	-	-
df165	0	2.5	342	0.3959	336	0.3662	-	-
df1831	0	7.7	343	0.3965	344	0.3765	-	-
df1319	0	24	344	0.3978	348	0.3791	-	-
df384	0	11.1	345	0.3987	347	0.3786	-	-
df2174	0	24	346	0.3995	349	0.3802	-	-
df2445	0	27.1	347	0.4005	359	0.3966	-	-

continued…

structure	duplicates	ΔH	rank	VC-xPWDF (3h)	rank	VC-xPWDF (2m)	rank	autoFIDEL
df871	0	9.2	348	0.4006	350	0.3818	-	-
df2491	0	18.9	349	0.4007	345	0.3766	-	-
df730	0	8.9	350	0.4020	361	0.3982	-	-
df2143	0	16.8	351	0.4024	343	0.3762	-	-
df1184	0	19.7	352	0.4052	358	0.3932	-	-
df2685	0	13.7	353	0.4056	346	0.3775	-	-
df742	0	14.8	354	0.4063	353	0.3874	-	-
df100	0	3.8	355	0.4076	354	0.3898	-	-
df2073	0	23.6	356	0.4086	351	0.3830	6	0.3437
df1042	0	21.1	357	0.4090	357	0.3919	-	-
df1844	0	13.8	358	0.4095	352	0.3860	-	-
df1950	0	25.6	359	0.4166	356	0.3914	-	-
df2148	0	26	360	0.4178	368	0.4053	-	-
df2260	0	28	361	0.4180	364	0.4012	-	-
df1246	0	21.9	362	0.4185	375	0.4254	-	-
df288	0	11.3	363	0.4200	360	0.3980	7	0.3577
df796	0	17.3	364	0.4204	363	0.4003	-	-
df2223	0	10.7	365	0.4206	367	0.4039	-	-
df2595	0	10.4	366	0.4210	355	0.3911	-	-
df976	0	8.8	367	0.4216	366	0.4019	-	-
df1871	0	13.6	368	0.4216	362	0.3990	-	-
df1884	0	15.4	369	0.4270	370	0.4146	-	-
df1233	0	15.2	370	0.4283	369	0.4078	-	-
df1425	0	12.1	371	0.4285	365	0.4019	-	-
df445	0	3.5	372	0.4293	374	0.4243	-	-
df645	0	3	373	0.4303	376	0.4269	-	-
df2678	0	25.2	374	0.4324	380	0.4305	-	-
df2438	0	27.6	375	0.4340	371	0.4195	-	-
df791	0	20.1	376	0.4390	384	0.4346	-	-
df2455	0	10.7	377	0.4413	378	0.4295	-	-
df2677	0	30.2	378	0.4432	381	0.4334	-	-
df546	0	15.9	379	0.4440	377	0.4273	-	-
df368	0	15.1	380	0.4447	372	0.4199	-	-
df275	0	4.9	381	0.4465	382	0.4338	-	-
df1035	0	21.5	382	0.4466	373	0.4216	-	-
df1983	0	22.4	383	0.4533	379	0.4302	-	-
df82	0	6.9	384	0.4598	383	0.4341	-	-
df1024	0	18.7	385	0.4602	386	0.4406	-	-
df1756	0	16.1	386	0.4620	393	0.4487	-	-
df1179	0	9.6	387	0.4634	391	0.4461	-	-
df2388	0	17.9	388	0.4637	394	0.4491	-	-
df933	0	10.4	389	0.4651	387	0.4423	-	-
df1947	0	17.3	390	0.4658	388	0.4425	-	-
df855	0	19.8	391	0.4665	385	0.4382	-	-

continued. . .

structure	duplicates	ΔH	rank	VC-xPWDF (3h)	rank	VC-xPWDF (2m)	rank	autoFIDEL
df857	0	19.3	392	0.4712	395	0.4503	-	-
df1192	0	19.1	393	0.4712	389	0.4449	-	-
df1430	0	20.7	394	0.4735	390	0.4456	-	-
df2492	0	29.1	395	0.4745	392	0.4485	-	-
df1146	0	24.2	396	0.4752	397	0.4587	-	-
df2006	0	13	397	0.4769	396	0.4574	-	-
df1732	0	22.5	398	0.4772	399	0.4602	-	-
df1351	0	21.5	399	0.4776	400	0.4608	-	-
df1259	0	22.9	400	0.4815	401	0.4610	-	-
df2297	0	13.9	401	0.4851	406	0.4658	-	-
df1535	0	24.5	402	0.4861	407	0.4665	-	-
df1881	0	18.2	403	0.4867	398	0.4589	-	-
df727	0	14.2	404	0.4869	403	0.4623	-	-
df2412	0	18.6	405	0.4874	404	0.4634	-	-
df321	0	10.3	406	0.4887	408	0.4675	-	-
df247	0	15.4	407	0.4888	405	0.4651	-	-
df1006	0	25.7	408	0.4908	410	0.4728	-	-
df899	0	2	409	0.4931	402	0.4618	-	-
df2117	0	21.4	410	0.4943	413	0.4747	-	-
df2441	0	25.6	411	0.4947	418	0.4819	-	-
df2077	0	24.8	412	0.4955	420	0.4828	-	-
df1046	0	21.4	413	0.4962	409	0.4697	-	-
df258	0	14.1	414	0.4973	429	0.4928	-	-
df1488	0	12.1	415	0.4976	415	0.4778	-	-
df1845	0	20.4	416	0.4998	411	0.4741	-	-
df206	0	9.8	417	0.5004	412	0.4744	-	-
df2381	0	26.5	418	0.5004	422	0.4843	-	-
df1570	0	21.2	419	0.5032	419	0.4823	-	-
df662	0	13	420	0.5036	425	0.4872	-	-
df2029	0	22.7	421	0.5067	417	0.4815	-	-
df529	0	17.1	422	0.5079	421	0.4828	-	-
df1018	0	21.1	423	0.5101	416	0.4803	-	-
df547	0	17.2	424	0.5103	426	0.4890	-	-
df2298	0	16.7	425	0.5112	414	0.4749	-	-
df836	0	2.5	426	0.5135	424	0.4870	-	-
df1876	1	15.3	427	0.5144	428	0.4900	-	-
df2257	0	20	428	0.5156	423	0.4867	-	-
df1124	0	13.6	429	0.5170	435	0.4953	-	-
df2599	0	16.2	430	0.5173	431	0.4935	-	-
df248	0	13.6	431	0.5175	446	0.5082	-	-
df1258	0	18	432	0.5179	434	0.4949	-	-
df1429	0	26.2	433	0.5186	432	0.4942	-	-
df2430	0	27.4	434	0.5189	433	0.4945	-	-
df1913	0	17.6	435	0.5197	430	0.4933	-	-

continued...

structure	duplicates	ΔH	rank	VC-xPWDF (3h)	rank	VC-xPWDF (2m)	rank	autoFIDEL
df823	0	16.6	436	0.5222	440	0.5018	-	-
df998	0	10.7	437	0.5223	436	0.4973	-	-
df2507	0	2.3	438	0.5225	447	0.5085	-	-
df1365	0	24.4	439	0.5228	438	0.5006	-	-
df1500	0	13.2	440	0.5242	439	0.5007	-	-
df2684	0	22.1	441	0.5243	437	0.4995	-	-
df347	0	11.2	442	0.5264	427	0.4899	-	-
df1955	0	7.3	443	0.5266	450	0.5130	-	-
df2444	0	8.2	444	0.5270	441	0.5058	-	-
df2198	0	21.4	445	0.5290	445	0.5080	-	-
df2617	0	13	446	0.5317	448	0.5095	-	-
df768	0	11.9	447	0.5321	449	0.5103	-	-
df1120	0	22.7	448	0.5339	443	0.5076	-	-
df2252	0	22.5	449	0.5358	455	0.5172	-	-
df1163	0	18.3	450	0.5365	444	0.5078	-	-
df1354	0	20.7	451	0.5367	454	0.5165	-	-
df1204	0	24	452	0.5371	452	0.5159	-	-
df914	0	9.4	453	0.5404	451	0.5134	-	-
df1427	0	24.9	454	0.5410	458	0.5215	-	-
df2166	0	19.6	455	0.5428	457	0.5184	-	-
df1991	0	17.8	456	0.5428	459	0.5259	-	-
df1457	0	22.9	457	0.5429	453	0.5165	-	-
df1775	0	15.1	458	0.5479	456	0.5173	-	-
df2587	0	22.3	459	0.5480	461	0.5283	-	-
df1518	0	25.7	460	0.5492	442	0.5073	-	-
df2101	0	24.8	461	0.5500	465	0.5337	-	-
df2500	0	14.1	462	0.5510	462	0.5286	-	-
df929	0	12.4	463	0.5512	463	0.5313	-	-
df814	0	15.3	464	0.5520	471	0.5402	-	-
df1298	0	22.7	465	0.5530	460	0.5277	-	-
df970	0	22	466	0.5539	467	0.5343	-	-
df2046	0	16.1	467	0.5539	469	0.5381	-	-
df603	0	7.9	468	0.5545	468	0.5347	-	-
df684	0	11.5	469	0.5553	477	0.5454	-	-
df215	0	10.7	470	0.5554	474	0.5435	-	-
df1174	0	20.2	471	0.5582	464	0.5322	-	-
df1883	0	22.6	472	0.5594	476	0.5447	-	-
df1098	0	18.1	473	0.5613	472	0.5418	-	-
df34	0	6.1	474	0.5625	473	0.5421	-	-
df1910	0	17	475	0.5628	466	0.5339	-	-
df2638	0	28.1	476	0.5654	482	0.5555	-	-
df1903	0	21.9	477	0.5666	470	0.5400	-	-
df2602	0	28.3	478	0.5732	480	0.5527	-	-
df388	0	10.1	479	0.5740	475	0.5439	-	-

continued...

structure	duplicates	ΔH	rank	VC-xPWDF (3h)	rank	VC-xPWDF (2m)	rank	autoFIDEL
df2428	0	23.5	480	0.5744	478	0.5490	-	-
df1044	0	19.9	481	0.5779	479	0.5513	-	-
df1203	0	18.8	482	0.5796	481	0.5533	-	-
df1735	0	26.7	483	0.5810	487	0.5603	-	-
df935	0	12.9	484	0.5814	483	0.5557	-	-
df1511	0	24.4	485	0.5834	488	0.5605	-	-
df562	0	10.3	486	0.5879	484	0.5583	-	-
df1134	0	14	487	0.5880	485	0.5585	-	-
df1869	0	21.6	488	0.5881	486	0.5603	-	-
df2254	0	27.8	489	0.5887	500	0.5718	-	-
df453	0	14.1	490	0.5897	495	0.5679	-	-
df2496	0	26	491	0.5899	501	0.5718	-	-
df2255	0	24.1	492	0.5904	498	0.5706	-	-
df1888	0	19.1	493	0.5904	497	0.5694	-	-
df565	0	16.7	494	0.5909	490	0.5636	10	0.3869
df1230	0	17.3	495	0.5910	491	0.5637	-	-
df1530	0	23.2	496	0.5922	494	0.5679	-	-
df2392	0	19.8	497	0.5928	489	0.5631	-	-
df1928	0	18.9	498	0.5933	512	0.5871	-	-
df629	0	19.5	499	0.5936	511	0.5859	-	-
df2251	0	14.3	500	0.5953	492	0.5658	-	-
df1745	0	21.4	501	0.5973	506	0.5808	-	-
df2019	0	22.7	502	0.5975	503	0.5765	-	-
df435	0	14	503	0.6012	505	0.5808	-	-
df1863	0	22.3	504	0.6026	499	0.5718	-	-
df2368	0	12	505	0.6026	496	0.5680	-	-
df1578	0	23.3	506	0.6037	508	0.5834	21	0.6875
df296	0	11.4	507	0.6073	493	0.5678	-	-
df1779	0	27.6	508	0.6081	507	0.5825	-	-
df1972	0	25.1	509	0.6085	513	0.5878	-	-
df1855	0	25.2	510	0.6087	504	0.5792	-	-
df1872	0	25.6	511	0.6101	510	0.5852	-	-
df408	0	8.2	512	0.6128	502	0.5729	-	-
df2533	0	26.2	513	0.6157	523	0.5977	-	-
df793	0	21	514	0.6185	521	0.5961	-	-
df2024	0	13	515	0.6187	514	0.5887	-	-
df1907	0	23.5	516	0.6187	520	0.5944	-	-
df915	0	15.7	517	0.6191	530	0.6033	-	-
df46	0	10.6	518	0.6193	526	0.5988	-	-
df1912	0	23.8	519	0.6199	522	0.5961	-	-
df1154	0	18.4	520	0.6214	519	0.5922	-	-
df1208	0	19.1	521	0.6215	515	0.5897	-	-
df1019	0	16.6	522	0.6218	517	0.5903	-	-
df1031	0	22.3	523	0.6226	509	0.5834	-	-

continued. . .

structure	duplicates	ΔH	rank	VC-xPWDF (3h)	rank	VC-xPWDF (2m)	rank	autoFIDEL
df1499	0	20.4	524	0.6244	525	0.5987	-	-
df2176	0	17.1	525	0.6251	516	0.5898	-	-
df1338	0	15.6	526	0.6260	518	0.5921	-	-
df1744	0	16.7	527	0.6268	528	0.6003	-	-
df2681	0	10.3	528	0.6281	529	0.6012	-	-
df2387	0	28.8	529	0.6290	538	0.6125	-	-
df1251	0	19.3	530	0.6295	527	0.5995	-	-
df1357	0	12.6	531	0.6304	531	0.6039	-	-
df848	0	17.3	532	0.6309	532	0.6049	-	-
df2243	0	19.2	533	0.6337	524	0.5980	-	-
df1521	0	15.4	534	0.6346	534	0.6068	-	-
df535	0	19.5	535	0.6349	537	0.6096	-	-
df2700	0	20.5	536	0.6351	533	0.6054	-	-
df1460	0	16.3	537	0.6365	547	0.6225	-	-
df1781	0	21	538	0.6368	535	0.6071	-	-
df751	0	15.4	539	0.6386	541	0.6145	-	-
df1327	0	14.6	540	0.6394	545	0.6196	-	-
df211	0	15.7	541	0.6394	543	0.6175	-	-
df1336	0	16.5	542	0.6412	546	0.6220	-	-
df726	0	17.8	543	0.6414	542	0.6146	-	-
df2119	0	24.5	544	0.6423	536	0.6093	-	-
df2011	0	22.2	545	0.6441	551	0.6266	-	-
df1170	0	15.9	546	0.6461	540	0.6144	-	-
df494	0	12.1	547	0.6470	544	0.6175	-	-
df2386	0	9.3	548	0.6497	550	0.6253	-	-
df1736	0	12.1	549	0.6498	539	0.6128	-	-
df2652	0	26.2	550	0.6501	556	0.6328	-	-
df387	0	12.9	551	0.6518	549	0.6251	-	-
df1206	0	14.4	552	0.6540	552	0.6294	-	-
df1547	0	17	553	0.6551	557	0.6342	-	-
df2488	0	24.3	554	0.6555	567	0.6404	-	-
df589	0	9.1	555	0.6558	561	0.6369	-	-
df643	0	14.1	556	0.6560	553	0.6299	-	-
df1801	0	11.2	557	0.6564	548	0.6238	-	-
df2067	0	25.6	558	0.6601	559	0.6347	-	-
df1963	0	23.6	559	0.6601	563	0.6392	8	0.3749
df1821	0	20.8	560	0.6615	560	0.6349	-	-
df209	0	15.7	561	0.6619	562	0.6383	-	-
df802	0	10.7	562	0.6621	565	0.6398	-	-
df1949	0	23.3	563	0.6623	555	0.6326	-	-
df1030	0	13.5	564	0.6629	566	0.6399	-	-
df1446	1	14	565	0.6631	570	0.6418	-	-
df1452	1	19.1	566	0.6640	571	0.6426	-	-
df1537	0	20.6	567	0.6660	558	0.6343	-	-

continued...

structure	duplicates	ΔH	rank	VC-xPWDF (3h)	rank	VC-xPWDF (2m)	rank	autoFIDEL
df1155	0	14	568	0.6671	569	0.6416	-	-
df1112	0	16.8	569	0.6680	568	0.6406	-	-
df1217	0	17.8	570	0.6685	564	0.6393	-	-
df1326	0	9.2	571	0.6691	589	0.6619	-	-
df345	0	15.3	572	0.6694	574	0.6445	-	-
df1234	0	12.5	573	0.6697	554	0.6304	-	-
df1758	0	23.1	574	0.6709	573	0.6438	-	-
df1610	0	21.9	575	0.6716	579	0.6502	-	-
df1905	0	19.7	576	0.6732	580	0.6507	-	-
df1045	0	17.6	577	0.6734	572	0.6437	-	-
df1770	0	24.9	578	0.6738	576	0.6464	-	-
df1545	0	21.4	579	0.6740	583	0.6562	-	-
df749	0	20.8	580	0.6745	575	0.6450	-	-
df2200	0	26.3	581	0.6749	582	0.6516	-	-
df780	0	17	582	0.6755	578	0.6489	-	-
df1295	0	24.2	583	0.6760	581	0.6515	-	-
df883	0	12.7	584	0.6781	577	0.6479	13	0.4320
df1613	0	20	585	0.6798	586	0.6576	-	-
df541	0	15.5	586	0.6810	585	0.6574	-	-
df265	0	16.1	587	0.6823	587	0.6578	-	-
df2506	0	27	588	0.6833	591	0.6633	-	-
df361	0	17.2	589	0.6844	584	0.6568	-	-
df1786	0	20.5	590	0.6870	590	0.6626	-	-
df2616	0	24.1	591	0.6872	593	0.6655	-	-
df1012	0	15.7	592	0.6876	592	0.6647	-	-
df30	0	11.1	593	0.6883	608	0.6752	-	-
df1986	0	20.1	594	0.6903	600	0.6707	-	-
df597	1	18.2	595	0.6904	596	0.6677	-	-
df1909	0	26.4	596	0.6907	598	0.6702	-	-
df1069	0	12.5	597	0.6908	595	0.6671	-	-
df1474	0	17.4	598	0.6911	597	0.6690	-	-
df282	0	16	599	0.6937	594	0.6660	-	-
df939	0	9.4	600	0.6941	588	0.6611	-	-
df316	0	14.3	601	0.6961	601	0.6718	-	-
df1156	0	18.4	602	0.6979	599	0.6706	-	-
df1253	0	14.8	603	0.6989	609	0.6754	-	-
df754	0	13.9	604	0.6996	606	0.6737	-	-
df1923	0	18	605	0.6997	602	0.6722	-	-
df1197	0	16.6	606	0.6998	603	0.6723	-	-
df2697	0	26.4	607	0.7010	610	0.6776	-	-
df986	0	14.7	608	0.7021	607	0.6749	-	-
df1964	0	26.2	609	0.7067	605	0.6736	-	-
df1815	0	22.2	610	0.7080	612	0.6820	-	-
df2167	0	28	611	0.7089	614	0.6829	-	-

continued...

structure	duplicates	ΔH	rank	VC-xPWDF (3h)	rank	VC-xPWDF (2m)	rank	autoFIDEL
df636	0	15.2	612	0.7098	611	0.6800	-	-
df865	0	12.4	613	0.7104	619	0.6877	-	-
df1369	0	17.3	614	0.7115	617	0.6847	-	-
df192	0	14.9	615	0.7116	630	0.6976	-	-
df1178	0	22.5	616	0.7117	615	0.6840	-	-
df429	0	15.2	617	0.7123	618	0.6850	-	-
df1952	0	23.9	618	0.7132	613	0.6825	-	-
df1171	0	14	619	0.7135	604	0.6728	-	-
df1557	0	23.8	620	0.7140	620	0.6878	-	-
df1738	0	21	621	0.7143	621	0.6901	-	-
df1439	0	12.2	622	0.7158	616	0.6844	-	-
df1148	0	21.1	623	0.7160	627	0.6956	-	-
df703	0	17	624	0.7183	623	0.6913	-	-
df816	0	13.7	625	0.7185	626	0.6953	-	-
df771	0	10.7	626	0.7194	628	0.6968	-	-
df307	0	13.6	627	0.7223	622	0.6904	-	-
df1822	0	18.9	628	0.7231	625	0.6939	-	-
df2603	0	20.6	629	0.7238	631	0.6992	-	-
df1895	0	23.9	630	0.7258	632	0.6994	-	-
df2007	0	24.1	631	0.7261	624	0.6927	-	-
df1240	0	22	632	0.7276	629	0.6969	-	-
df1491	0	14.2	633	0.7310	634	0.7039	-	-
df858	0	15.4	634	0.7314	636	0.7054	-	-
df1860	0	18.6	635	0.7317	638	0.7072	12	0.4144
df755	0	21.1	636	0.7320	642	0.7088	-	-
df466	0	16.5	637	0.7327	646	0.7136	-	-
df176	0	14.4	638	0.7330	655	0.7196	-	-
df593	0	16.3	639	0.7333	639	0.7079	-	-
df2414	1	24.4	640	0.7354	649	0.7157	-	-
df1102	0	19.5	641	0.7362	640	0.7081	-	-
df1010	0	17.3	642	0.7366	645	0.7118	-	-
df2030	0	16.6	643	0.7391	637	0.7071	-	-
df1899	0	21.1	644	0.7396	647	0.7136	-	-
df533	0	9.9	645	0.7397	648	0.7140	-	-
df1447	0	23.5	646	0.7400	644	0.7111	-	-
df2061	0	29.2	647	0.7404	661	0.7252	-	-
df651	0	18.2	648	0.7405	654	0.7179	-	-
df2268	0	16.7	649	0.7408	656	0.7209	-	-
df1672	0	26.7	650	0.7432	650	0.7163	-	-
df479	0	13.1	651	0.7440	635	0.7051	-	-
df728	0	20.9	652	0.7441	651	0.7168	-	-
df2129	0	17.7	653	0.7441	657	0.7210	-	-
df700	0	13.9	654	0.7449	643	0.7093	-	-
df1862	0	23.3	655	0.7468	659	0.7223	-	-

continued. . .

structure	duplicates	ΔH	rank	VC-xPWDF (3h)	rank	VC-xPWDF (2m)	rank	autoFIDEL
df1495	0	11.7	656	0.7470	663	0.7264	-	-
df1139	0	20.8	657	0.7471	633	0.7026	-	-
df1175	0	14.9	658	0.7481	653	0.7176	-	-
df637	0	12.5	659	0.7490	652	0.7174	-	-
df615	0	19.5	660	0.7491	660	0.7250	-	-
df2084	0	26.4	661	0.7500	665	0.7302	-	-
df551	0	12.9	662	0.7520	641	0.7088	-	-
df971	0	13	663	0.7526	666	0.7307	-	-
df2619	0	30.2	664	0.7526	658	0.7223	-	-
df1125	0	15.9	665	0.7550	662	0.7259	-	-
df959	0	20.4	666	0.7590	672	0.7405	-	-
df549	0	18.9	667	0.7607	667	0.7353	-	-
df2081	0	17	668	0.7616	671	0.7386	-	-
df499	0	9.3	669	0.7617	664	0.7289	-	-
df534	0	13.3	670	0.7633	673	0.7408	-	-
df956	0	21.9	671	0.7651	670	0.7380	-	-
df1431	0	22.9	672	0.7663	668	0.7378	-	-
df2426	0	24.3	673	0.7667	675	0.7447	-	-
df720	0	20.4	674	0.7682	674	0.7421	-	-
df1804	0	27.2	675	0.7703	676	0.7448	-	-
df1797	0	16.1	676	0.7739	678	0.7463	-	-
df2060	0	19	677	0.7746	669	0.7378	-	-
df1616	0	15.3	678	0.7756	679	0.7471	-	-
df822	0	15.7	679	0.7765	680	0.7484	-	-
df1524	0	15.2	680	0.7789	683	0.7548	-	-
df1214	0	20.1	681	0.7812	681	0.7504	-	-
df2686	0	24.6	682	0.7815	687	0.7591	-	-
df1140	0	10.6	683	0.7825	690	0.7619	-	-
df2457	0	15.9	684	0.7833	691	0.7630	-	-
df913	0	17.5	685	0.7834	677	0.7454	-	-
df1856	0	13.2	686	0.7848	682	0.7547	-	-
df146	0	10	687	0.7858	686	0.7590	-	-
df1115	0	19.4	688	0.7868	700	0.7692	-	-
df573	0	15.4	689	0.7914	698	0.7683	-	-
df583	0	14	690	0.7917	684	0.7549	-	-
df1730	0	22.3	691	0.7922	704	0.7712	-	-
df584	0	16	692	0.7922	697	0.7682	-	-
df797	0	12.9	693	0.7927	706	0.7733	-	-
df1238	0	19.3	694	0.7930	693	0.7646	-	-
df906	0	20.1	695	0.7930	702	0.7696	-	-
df1522	0	24.6	696	0.7940	688	0.7599	-	-
df1594	0	18.3	697	0.7944	699	0.7691	-	-
df1389	0	14.4	698	0.7952	696	0.7676	-	-
df44	0	11.2	699	0.7973	705	0.7725	-	-

continued...

structure	duplicates	ΔH	rank	VC-xPWDF (3h)	rank	VC-xPWDF (2m)	rank	autoFIDEL
df2698	0	26.2	700	0.7980	695	0.7676	-	-
df1615	0	17.5	701	0.7992	689	0.7602	-	-
df799	0	16.7	702	0.7998	701	0.7693	-	-
df478	0	12.1	703	0.8012	685	0.7577	-	-
df512	0	17.6	704	0.8033	709	0.7788	-	-
df1211	0	23.8	705	0.8037	703	0.7707	-	-
df1513	0	20.4	706	0.8050	710	0.7789	-	-
df1916	0	22	707	0.8053	708	0.7786	-	-
df882	0	20.4	708	0.8058	719	0.7849	-	-
df2440	0	24	709	0.8060	726	0.7924	-	-
df1740	0	26.4	710	0.8062	712	0.7796	-	-
df1037	0	22.3	711	0.8066	713	0.7799	-	-
df543	0	14.6	712	0.8073	718	0.7848	-	-
df2236	0	30.4	713	0.8096	714	0.7805	2	0.1313
df1229	0	19.9	714	0.8102	694	0.7663	-	-
df1461	0	9.7	715	0.8104	707	0.7784	-	-
df1915	0	23.6	716	0.8105	692	0.7645	-	-
df564	0	16.9	717	0.8108	717	0.7831	-	-
df1239	0	22.5	718	0.8109	723	0.7876	-	-
df1423	0	20.6	719	0.8114	715	0.7809	-	-
df869	0	20.8	720	0.8116	731	0.7961	-	-
df544	0	16.6	721	0.8124	722	0.7867	-	-
df2318	0	23.2	722	0.8134	725	0.7914	-	-
df1047	0	12.6	723	0.8137	724	0.7895	-	-
df2431	0	18.6	724	0.8139	716	0.7831	14	0.4771
df1223	0	19.2	725	0.8149	711	0.7792	-	-
df1748	0	16.1	726	0.8151	730	0.7945	-	-
df1318	0	26.4	727	0.8180	728	0.7940	-	-
df2162	0	30.5	728	0.8181	736	0.8043	-	-
df1889	0	25.4	729	0.8191	721	0.7853	-	-
df1975	0	29.4	730	0.8201	729	0.7944	-	-
df450	0	15.1	731	0.8210	720	0.7851	3	0.1762
df2604	0	25.3	732	0.8222	732	0.7986	-	-
df763	0	13.9	733	0.8245	727	0.7931	-	-
df2327	0	13.4	734	0.8251	735	0.8032	-	-
df285	0	10.1	735	0.8255	733	0.8000	-	-
df1316	0	23.9	736	0.8259	744	0.8123	-	-
df1129	0	18.4	737	0.8274	734	0.8017	17	0.4944
df740	0	20.1	738	0.8288	738	0.8061	-	-
df600	0	7.6	739	0.8293	737	0.8060	-	-
df2429	0	26.5	740	0.8306	745	0.8143	-	-
df1890	0	22.5	741	0.8310	740	0.8079	-	-
df1998	0	24.4	742	0.8339	743	0.8100	-	-
df840	0	20.1	743	0.8344	741	0.8092	-	-

continued…

structure	duplicates	ΔH	rank	VC-xPWDF (3h)	rank	VC-xPWDF (2m)	rank	autoFIDEL
df1477	0	12.8	744	0.8345	739	0.8075	-	-
df2453	0	21.7	745	0.8405	747	0.8147	-	-
df219	0	16	746	0.8408	742	0.8097	-	-
df1227	0	23	747	0.8424	746	0.8146	-	-
df2294	0	16.3	748	0.8430	750	0.8231	-	-
df2410	0	23.8	749	0.8458	753	0.8256	-	-
df563	0	15.7	750	0.8484	751	0.8235	-	-
df1739	0	10.4	751	0.8484	748	0.8174	-	-
df2375	0	25.2	752	0.8510	749	0.8216	-	-
df675	0	13.3	753	0.8536	752	0.8245	-	-
df1958	0	24	754	0.8562	754	0.8276	-	-
df960	0	24.4	755	0.8565	756	0.8386	-	-
df238	0	9	756	0.8590	755	0.8323	-	-
df1722	0	23.9	757	0.8652	758	0.8463	-	-
df2435	0	21.4	758	0.8659	759	0.8464	-	-
df1323	0	21.3	759	0.8667	757	0.8439	-	-
df2269	0	23	760	0.8694	762	0.8531	-	-
df721	0	8.3	761	0.8709	764	0.8535	-	-
df414	0	19	762	0.8720	760	0.8488	-	-
df555	0	11	763	0.8734	761	0.8505	-	-
df815	0	12	764	0.8759	768	0.8637	-	-
df2673	0	19.6	765	0.8784	763	0.8533	-	-
df137	0	12.4	766	0.8787	765	0.8557	-	-
df251	0	16.8	767	0.8817	769	0.8681	-	-
df462	0	11.9	768	0.8832	771	0.8703	-	-
df572	0	17.7	769	0.8835	766	0.8594	-	-
df2456	0	27.8	770	0.8843	770	0.8692	-	-
df1798	0	27.7	771	0.8864	767	0.8603	-	-
df1764	0	24.1	772	0.8924	772	0.8717	-	-
df1011	0	8	773	0.9058	773	0.8840	-	-
df2071	0	26.3	774	0.9059	776	0.8956	-	-
df328	0	17.2	775	0.9125	775	0.8905	-	-
df548	0	13.1	776	0.9148	774	0.8896	-	-
df1508	0	19.8	777	0.9176	779	0.9047	-	-
df2451	0	24.1	778	0.9254	781	0.9055	-	-
df1552	0	30.8	779	0.9257	778	0.9046	-	-
df2118	0	13.7	780	0.9261	777	0.9041	-	-
df1772	0	19.1	781	0.9271	782	0.9061	-	-
df2256	0	17.9	782	0.9273	780	0.9051	-	-
df1118	0	24.7	783	0.9392	783	0.9237	-	-

Table S8: Summary of data for 1,4-dicyanobenzene. Relative enthalpy is given in kJ/mol, ranks are according to the next-right column, autoFIDEL comparisons were made with the default program parameters and the experimental powder diffractograms collected over 3 hrs.

structure	duplicates	ΔH	rank	VC-xPWDF (3h)	rank	VC-xPWDF (2m)	rank	autoFIDEL
TEPNIT04	-	-	1	0.0326	1	0.0335	1	0.0934
TEPNIT14	-	-	2	0.0330	2	0.0339	-	-
opt_ca99	8	0	3	0.0366	3	0.0372	21	0.4462
opt_am133	0	1.4	4	0.1165	4	0.1184	-	-
opt_bf110	0	19.6	5	0.1261	5	0.1213	-	-
opt_aa88	0	0.8	6	0.1418	6	0.1441	3	0.2614
opt_ab127	0	0.8	7	0.1426	8	0.1449	2	0.1849
opt_ca121	0	0.6	8	0.1436	9	0.1459	11	0.3229
opt_da10	3	10.2	9	0.1465	7	0.1445	-	-
opt_db20	0	0.8	10	0.1750	10	0.1710	5	0.2783
opt_ak55	3	1.3	11	0.1920	12	0.1912	-	-
opt_db41	0	4.4	12	0.1935	11	0.1899	-	-
opt_av67	0	1.5	13	0.2000	14	0.1997	-	-
opt_am100	0	8.1	14	0.2011	13	0.1989	-	-
opt_da41	0	5.5	15	0.2525	15	0.2486	12	0.3384
opt_az131	0	13.8	16	0.2590	16	0.2517	-	-
opt_ce4	0	9	17	0.2697	17	0.2700	-	-
opt_da58	1	6.3	18	0.2800	19	0.2758	-	-
opt_bh86	0	19.6	19	0.2810	20	0.2783	-	-
opt_bf97	0	7	20	0.2846	18	0.2745	-	-
opt_ai59	1	2	21	0.2895	21	0.2841	4	0.2691
opt_da28	3	4.6	22	0.2896	24	0.2900	-	-
opt_da73	0	7.5	23	0.2911	23	0.2867	27	0.6377
opt_am152	3	8	24	0.2915	22	0.2846	-	-
opt_ak126	0	9.4	25	0.3133	25	0.3073	-	-
opt_aq110	0	10.6	26	0.3282	26	0.3174	-	-
opt_aq105	5	1.4	27	0.3307	27	0.3238	-	-
opt_bd88	0	6.8	28	0.3394	29	0.3381	-	-
opt_az102	0	4.4	29	0.3397	28	0.3362	-	-
opt_aq142	0	11.5	30	0.3516	30	0.3451	-	-
opt_av30	0	3.5	31	0.3562	31	0.3497	10	0.3098
opt_bd53	0	12.6	32	0.3601	32	0.3568	-	-
opt_am126	0	5.8	33	0.3681	33	0.3618	26	0.4913
opt_al48	0	2.9	34	0.3758	34	0.3699	7	0.2827
opt_af47	0	2.8	35	0.3800	35	0.3758	19	0.4254
opt_au50	0	6.2	36	0.3872	36	0.3798	-	-
opt_ai128	0	2.3	37	0.3896	37	0.3830	20	0.4435
opt_ad74	1	4.8	38	0.4063	38	0.3967	28	0.6572
opt_cc80	0	4.3	39	0.4066	39	0.3969	-	-
opt_db32	0	3.4	40	0.4145	41	0.4071	18	0.4253
opt_au118	1	4.1	41	0.4162	40	0.4066	13	0.3483

continued...

structure	duplicates	ΔH	rank	VC-xPWDF (3h)	rank	VC-xPWDF (2m)	rank	autoFIDEL
opt_ba61	0	2.2	42	0.4215	42	0.4147	-	-
TEPNIT06	-	-	43	0.4339	44	0.4256	-	-
opt_af30	0	2.7	44	0.4362	45	0.4300	-	-
opt_aq126	0	16.3	45	0.4364	43	0.4244	-	-
opt_au111	0	10.9	46	0.4405	46	0.4312	17	0.4132
opt_au19	0	2.1	47	0.4576	50	0.4545	-	-
opt_am97	0	9.3	48	0.4603	47	0.4504	-	-
opt_ak38	0	2.8	49	0.4612	48	0.4522	22	0.4597
opt_ay126	0	6.8	50	0.4616	49	0.4526	-	-
opt_ah34	2	8.6	51	0.4675	51	0.4606	-	-
opt_al95	0	3.2	52	0.4737	52	0.4651	-	-
opt_ay100	0	3.3	53	0.4832	53	0.4735	29	0.6767
opt_av96	4	1.4	54	0.4858	54	0.4810	-	-
opt_aq116	0	15.4	55	0.4972	55	0.4852	-	-
opt_dc32	0	2.4	56	0.4989	56	0.4891	6	0.2802
opt_ah31	1	8.7	57	0.5001	57	0.4932	-	-
opt_ay84	0	3.6	58	0.5079	58	0.4950	14	0.3506
opt_dc10	0	0.6	59	0.5138	60	0.5044	8	0.3060
opt_au40	2	1.6	60	0.5149	61	0.5121	-	-
opt_aq33	0	19.1	61	0.5172	59	0.5029	-	-
opt_az62	3	1.9	62	0.5470	62	0.5333	15	0.3584
opt_ay129	0	12.6	63	0.5483	63	0.5370	-	-
opt_ah53	0	6.8	64	0.5537	64	0.5432	-	-
opt_am15	0	7.3	65	0.5725	66	0.5592	-	-
opt_ay152	0	7.3	66	0.5731	65	0.5584	-	-
opt_cc83	0	19.4	67	0.5848	67	0.5731	-	-
opt_cd146	0	8.4	68	0.5909	69	0.5834	-	-
opt_ay35	1	8.6	69	0.5909	68	0.5822	-	-
opt_am39	0	18.8	70	0.5980	70	0.5834	-	-
opt_ad27	1	3.2	71	0.6153	71	0.6045	-	-
opt_aj87	0	8.1	72	0.6206	73	0.6177	-	-
opt_aj89	0	8.4	73	0.6210	74	0.6180	-	-
opt_ay108	0	13.1	74	0.6232	72	0.6139	-	-
opt_aj21	1	8	75	0.6276	76	0.6247	-	-
opt_ay10	4	8.9	76	0.6284	75	0.6222	-	-
opt_au10	5	9.1	77	0.6329	77	0.6273	-	-
opt_au88	0	12.9	78	0.6634	78	0.6501	-	-
opt_bd37	0	5.1	79	0.6771	81	0.6671	-	-
opt_cb111	0	15.2	80	0.6779	79	0.6652	25	0.4808
opt_aw19	0	3.1	81	0.6785	80	0.6671	23	0.4654
opt_ak146	0	14	82	0.6810	83	0.6752	-	-
opt_cc9	0	18.1	83	0.6855	82	0.6746	-	-
opt_cd106	3	7.7	84	0.6873	84	0.6757	-	-
opt_cb27	1	7.8	85	0.6882	85	0.6768	-	-

continued...

structure	duplicates	ΔH	rank	VC-xPWDF (3h)	rank	VC-xPWDF (2m)	rank	autoFIDEL
opt_cb72	0	6.9	86	0.6926	86	0.6819	-	-
opt_aw57	0	6.6	87	0.7028	87	0.6878	-	-
opt_bh63	0	4.9	88	0.7055	88	0.6958	-	-
opt_cc97	0	19.3	89	0.7240	89	0.7093	-	-
opt_af40	1	8	90	0.7302	90	0.7250	-	-
opt_am63	0	4	91	0.7510	91	0.7371	-	-
opt_cd60	0	10	92	0.7555	93	0.7487	24	0.4791
opt_cb130	0	9.9	93	0.7611	92	0.7445	-	-
opt_aw24	2	10.8	94	0.7649	94	0.7523	-	-
opt_ak127	0	9.4	95	0.7866	95	0.7755	9	0.3069
opt_ak129	0	6.3	96	0.7879	96	0.7769	16	0.3865
opt_cb123	1	8.9	97	0.7986	97	0.7813	-	-

Table S9: Summary of data for uracil. Relative enthalpy is given in kJ/mol, ranks are according to the next-right column, autoFIDEL comparisons were made with the default program parameters and the experimental powder diffractograms collected over 3 hrs.

structure	duplicates	ΔH	rank	VC-xPWDF (3h)	rank	VC-xPWDF (2m)	rank	autoFIDEL
URACIL	-	-	1	0.0290	1	0.0382	1	0.1392
opt_am7	0	1.2	2	0.0297	2	0.0391	-	-
opt_am82	0	1.7	3	0.0358	3	0.0435	-	-
opt_am72	0	3.2	4	0.0659	4	0.0739	-	-
opt_aq92	0	5.5	5	0.0869	5	0.0896	-	-
opt_ay85	0	8.8	6	0.0984	7	0.1011	-	-
opt_am75	1	3.4	7	0.1001	8	0.1013	-	-
opt_aw23	0	6.4	8	0.1018	9	0.1035	-	-
opt_am103	0	5.5	9	0.1052	6	0.1000	-	-
opt_aq5	0	3.7	10	0.1090	10	0.1088	-	-
opt_av96	0	6.9	11	0.1112	11	0.1132	-	-
opt_av62	0	4.6	12	0.1146	12	0.1138	-	-
opt_av23	1	4.6	13	0.1154	13	0.1150	-	-
opt_fa88	0	6.3	14	0.1177	15	0.1199	-	-
opt_am100	0	0.1	15	0.1215	21	0.1264	8	0.3053
opt_av85	0	7.1	16	0.1222	17	0.1204	-	-
opt_av8	0	2.8	17	0.1232	19	0.1244	-	-
opt_am36	0	0.5	18	0.1234	20	0.1246	-	-
opt_am67	0	9	19	0.1256	18	0.1215	-	-
opt_ap78	0	3.8	20	0.1256	16	0.1200	-	-
opt_am1	0	7.4	21	0.1256	14	0.1197	-	-
opt_ap38	0	2.1	22	0.1273	22	0.1272	-	-
opt_ab40	0	1.1	23	0.1304	25	0.1393	-	-
opt_dd16	0	1.2	24	0.1358	26	0.1404	-	-
opt_av43	0	7.5	25	0.1372	23	0.1375	-	-
opt_dc6	0	0.8	26	0.1421	29	0.1480	-	-
opt_av7	0	7.7	27	0.1422	24	0.1382	-	-
opt_af76	0	3	28	0.1448	31	0.1514	5	0.2977
opt_ab72	0	0.6	29	0.1449	33	0.1532	-	-
opt_ak27	0	7.8	30	0.1475	28	0.1467	-	-
opt_ak9	0	8	31	0.1509	27	0.1450	-	-
opt_al57	1	5.6	32	0.1524	30	0.1507	-	-
opt_ai43	0	6.3	33	0.1534	32	0.1525	44	0.5007
opt_av53	0	8.4	34	0.1539	34	0.1534	-	-
opt_ad41	0	2.3	35	0.1570	35	0.1591	14	0.3679
opt_aj1	0	4.7	36	0.1620	37	0.1621	-	-
opt_am65	0	1.7	37	0.1661	40	0.1673	-	-
opt_aq88	0	6.6	38	0.1664	38	0.1655	-	-
opt_ar11	0	4.1	39	0.1671	36	0.1612	-	-
opt_ai48	0	6.9	40	0.1708	41	0.1699	29	0.4091
opt_av32	0	6.2	41	0.1724	42	0.1723	27	0.4068

continued…

structure	duplicates	ΔH	rank	VC-xPWDF (3h)	rank	VC-xPWDF (2m)	rank	autoFIDEL
opt_av77	0	8.8	42	0.1736	39	0.1665	-	-
opt_bj20	0	5.1	43	0.1750	44	0.1779	-	-
opt_au19	0	2.5	44	0.1811	43	0.1729	47	0.5174
opt_af4	0	8.3	45	0.1909	47	0.1899	22	0.3957
opt_ai81	0	6.3	46	0.1916	46	0.1873	-	-
opt_av63	0	9.4	47	0.1922	45	0.1864	-	-
opt_db20	0	6.8	48	0.1933	53	0.1986	-	-
opt_aq3	0	6.5	49	0.1956	51	0.1930	-	-
opt_am28	0	3.3	50	0.1959	49	0.1901	-	-
opt_cb64	0	2.2	51	0.1969	48	0.1900	-	-
opt_au35	0	6.3	52	0.1972	50	0.1912	-	-
opt_af45	0	5	53	0.1996	52	0.1963	-	-
opt_af7	0	4.3	54	0.2028	54	0.2014	21	0.3956
opt_ai61	0	6.5	55	0.2084	56	0.2081	-	-
opt_db24	0	8.3	56	0.2114	55	0.2066	-	-
opt_ak73	0	6.2	57	0.2177	57	0.2124	-	-
opt_ai14	0	5.2	58	0.2194	58	0.2142	-	-
opt_de31	0	6.9	59	0.2282	61	0.2227	-	-
opt_db58	0	7	60	0.2301	62	0.2235	-	-
opt_fc5	0	4.8	61	0.2303	60	0.2217	-	-
opt_aq73	0	4.6	62	0.2326	59	0.2208	38	0.4509
opt_fc35	0	4.8	63	0.2346	63	0.2249	-	-
opt_bj4	0	5.6	64	0.2346	64	0.2264	-	-
opt_de20	0	3.8	65	0.2351	65	0.2266	30	0.4136
opt_bh51	0	6.1	66	0.2352	66	0.2288	-	-
opt_cc78	0	9.7	67	0.2358	68	0.2365	33	0.4215
opt_de67	0	5.1	68	0.2360	67	0.2301	39	0.4617
opt_db50	0	6.7	69	0.2459	69	0.2379	-	-
opt_dd4	0	5.3	70	0.2488	70	0.2393	-	-
opt_ai89	0	7.2	71	0.2492	71	0.2429	-	-
opt_aq9	0	5.4	72	0.2516	72	0.2437	-	-
opt_de66	0	7.9	73	0.2524	75	0.2475	-	-
opt_ad3	0	4.4	74	0.2527	73	0.2468	-	-
opt_ak75	0	3	75	0.2571	74	0.2472	-	-
opt_db56	0	8.4	76	0.2577	76	0.2487	-	-
opt_av17	0	1.8	77	0.2598	77	0.2499	-	-
opt_bd50	0	6.9	78	0.2623	79	0.2519	35	0.4316
opt_az72	0	5.5	79	0.2630	80	0.2546	45	0.5086
opt_ai41	0	3.2	80	0.2635	78	0.2512	-	-
opt_aw34	0	7.4	81	0.2650	81	0.2570	-	-
opt_fa50	0	2.6	82	0.2663	86	0.2701	18	0.3887
opt_ay13	0	2.9	83	0.2706	82	0.2636	-	-
opt_aj60	0	7.9	84	0.2724	84	0.2640	-	-
opt_bb55	0	4.6	85	0.2758	83	0.2639	24	0.4023

continued...

structure	duplicates	ΔH	rank	VC-xPWDF (3h)	rank	VC-xPWDF (2m)	rank	autoFIDEL
opt_ai94	0	8.5	86	0.2760	85	0.2663	-	-
opt_aj49	0	6.9	87	0.2853	87	0.2703	-	-
opt_cd81	0	3.7	88	0.2864	93	0.2834	-	-
opt_az32	0	7.5	89	0.2881	88	0.2761	-	-
opt_bd56	0	4.8	90	0.2886	91	0.2821	11	0.3554
opt_de71	0	7	91	0.2895	89	0.2777	48	0.5181
opt_de78	0	8.1	92	0.2915	92	0.2824	-	-
opt_aj37	0	6.9	93	0.2972	90	0.2813	-	-
opt_cd102	0	4	94	0.2973	99	0.2920	55	0.6004
opt_al19	0	7.8	95	0.3009	95	0.2885	-	-
opt_fc51	0	2.2	96	0.3011	98	0.2916	3	0.2858
opt_bj91	0	5.9	97	0.3021	97	0.2901	-	-
opt_al39	0	6.1	98	0.3022	96	0.2898	-	-
opt_de39	0	4	99	0.3047	94	0.2862	-	-
opt_fa83	0	6.2	100	0.3187	102	0.3117	-	-
opt_au27	0	9.6	101	0.3191	100	0.3079	-	-
opt_cc89	0	9	102	0.3200	103	0.3129	46	0.5117
opt_ar70	0	4.1	103	0.3232	105	0.3194	-	-
opt_cd66	0	6.8	104	0.3237	101	0.3088	-	-
opt_bb20	0	6	105	0.3272	106	0.3199	-	-
opt_au23	0	7.7	106	0.3283	107	0.3207	-	-
opt_bf65	0	2.7	107	0.3295	104	0.3192	-	-
opt_bb38	0	8.4	108	0.3297	109	0.3238	-	-
opt_cb66	0	0.9	109	0.3307	108	0.3233	-	-
opt_cc93	0	3.4	110	0.3415	111	0.3277	52	0.5483
opt_bh30	0	9.5	111	0.3415	112	0.3310	-	-
opt_fa15	0	1.9	112	0.3420	113	0.3319	40	0.4724
opt_ai77	0	7.9	113	0.3430	115	0.3335	23	0.3971
opt_cc34	2	1.7	114	0.3466	110	0.3263	-	-
opt_ai64	0	7.1	115	0.3466	116	0.3340	-	-
opt_ar25	0	7.5	116	0.3473	117	0.3402	51	0.5394
opt_de82	0	4.8	117	0.3491	114	0.3329	-	-
opt_cc12	0	6.5	118	0.3524	121	0.3444	34	0.4288
opt_aj68	0	8.2	119	0.3527	118	0.3422	-	-
opt_de2	0	5.9	120	0.3564	120	0.3428	-	-
opt_dc18	0	6.1	121	0.3573	119	0.3424	-	-
opt_aj17	0	6.8	122	0.3607	122	0.3515	-	-
opt_az5	0	6.2	123	0.3674	123	0.3584	37	0.4389
opt_al59	0	7.4	124	0.3760	125	0.3612	-	-
opt_aw66	0	7.4	125	0.3789	128	0.3710	-	-
opt_bj89	0	8.6	126	0.3803	127	0.3690	42	0.4790
opt_av40	0	4.7	127	0.3805	126	0.3668	54	0.5996
opt_dc27	0	1.8	128	0.3816	124	0.3596	-	-
opt_cd105	0	0	129	0.3830	134	0.3841	-	-

continued...

structure	duplicates	ΔH	rank	VC-xPWDF (3h)	rank	VC-xPWDF (2m)	rank	autoFIDEL
opt_ba38	0	7.7	130	0.3854	129	0.3717	-	-
opt_ay10	0	8.2	131	0.3915	132	0.3810	-	-
opt_fc42	0	3.9	132	0.3926	136	0.3863	-	-
opt_be13	0	2.9	133	0.3950	133	0.3812	25	0.4031
opt_ai73	0	8.4	134	0.3962	135	0.3859	-	-
opt_fc91	0	1.7	135	0.3968	130	0.3798	17	0.3863
opt_dc64	0	7.4	136	0.3980	131	0.3802	-	-
opt_fa29	0	1.2	137	0.4021	137	0.3879	-	-
opt_fc82	0	1.3	138	0.4082	138	0.3907	-	-
opt_au79	0	2.1	139	0.4088	139	0.3962	12	0.3567
opt_fa52	0	8.3	140	0.4129	140	0.3992	-	-
opt_ca59	0	0.6	141	0.4158	141	0.4034	-	-
opt_cb53	0	4.4	142	0.4212	142	0.4105	-	-
opt_aj36	0	6.7	143	0.4232	143	0.4131	-	-
opt_dc34	0	9	144	0.4304	145	0.4219	-	-
opt_ce67	0	8.9	145	0.4313	147	0.4227	31	0.4157
opt_fa87	0	4.4	146	0.4331	144	0.4176	-	-
opt_ak24	0	6.9	147	0.4379	146	0.4222	41	0.4769
opt_av1	0	5.5	148	0.4420	150	0.4320	26	0.4059
opt_ai56	0	8.4	149	0.4463	149	0.4293	53	0.5726
opt_fc47	0	2.3	150	0.4468	154	0.4376	-	-
opt_dc53	0	3.1	151	0.4483	148	0.4266	28	0.4084
opt_cb11	0	4.2	152	0.4488	152	0.4353	-	-
opt_aj22	0	9.3	153	0.4493	151	0.4345	-	-
opt_ca96	0	0.3	154	0.4543	153	0.4358	15	0.3712
opt_bh24	0	4.8	155	0.4571	156	0.4448	-	-
opt_cd67	0	9.7	156	0.4575	157	0.4533	49	0.5253
opt_bb19	0	4.6	157	0.4578	155	0.4431	-	-
opt_au62	0	8.6	158	0.4708	161	0.4613	-	-
opt_dc10	1	7.2	159	0.4714	158	0.4563	-	-
opt_al12	0	6.9	160	0.4739	159	0.4581	-	-
opt_fc13	0	7.7	161	0.4752	160	0.4601	-	-
opt_fa79	0	5.1	162	0.4793	162	0.4677	-	-
opt_am81	0	1.3	163	0.4793	163	0.4683	13	0.3613
opt_fa23	0	1	164	0.4854	165	0.4736	9	0.3083
opt_cc80	0	8.6	165	0.4860	164	0.4690	-	-
opt_cb96	0	8.2	166	0.5032	167	0.4894	-	-
opt_aj4	0	8.5	167	0.5056	168	0.4897	-	-
opt_ai35	0	9	168	0.5097	166	0.4881	-	-
opt_fc27	0	5.2	169	0.5104	169	0.4950	-	-
opt_ay96	0	1.1	170	0.5144	170	0.4973	-	-
opt_ca17	0	2.1	171	0.5155	171	0.5017	-	-
opt_da12	0	0	172	0.5169	172	0.5041	-	-
opt_cc19	0	7.2	173	0.5208	176	0.5146	-	-

continued. . .

structure	duplicates	ΔH	rank	VC-xPWDF (3h)	rank	VC-xPWDF (2m)	rank	autoFIDEL
opt_fc60	0	4.4	174	0.5233	173	0.5061	-	-
opt_ai58	0	6.9	175	0.5263	174	0.5079	-	-
opt_cb100	0	1.5	176	0.5265	175	0.5083	-	-
opt_cd11	0	5.5	177	0.5326	181	0.5244	-	-
opt_ce29	0	2.7	178	0.5338	178	0.5183	32	0.4160
opt_ce68	0	7.8	179	0.5357	177	0.5165	36	0.4363
opt_cc73	0	7.8	180	0.5419	180	0.5239	-	-
opt_cc2	0	3.3	181	0.5426	179	0.5224	-	-
opt_dd18	0	7.3	182	0.5430	182	0.5272	-	-
opt_fc90	0	4.6	183	0.5449	183	0.5276	7	0.3037
opt_ce45	0	4.7	184	0.5453	184	0.5299	56	0.6270
opt_ai87	0	9.4	185	0.5483	187	0.5408	-	-
opt_ay84	0	5.5	186	0.5500	186	0.5355	-	-
opt_da104	0	3.4	187	0.5538	185	0.5352	-	-
opt_fc79	0	4.8	188	0.5543	188	0.5441	10	0.3428
opt_cd1	0	2.1	189	0.5547	189	0.5482	50	0.5391
opt_ai97	0	5.5	190	0.5701	190	0.5541	-	-
opt_cd7	0	1.7	191	0.5843	192	0.5779	-	-
opt_dc88	0	9.5	192	0.5875	191	0.5739	-	-
opt_cd78	0	5.5	193	0.5950	194	0.5878	-	-
opt_cc37	0	9.2	194	0.6039	193	0.5842	-	-
opt_ab44	0	2.2	195	0.6221	195	0.6046	19	0.3917
opt_cb99	0	0.8	196	0.6249	196	0.6137	43	0.4822
opt_ar60	0	8.2	197	0.6297	197	0.6165	-	-
opt_am2	0	0.8	198	0.6373	198	0.6195	-	-
opt_cb1	0	6.3	199	0.6416	199	0.6336	-	-
opt_am10	0	0.7	200	0.6640	200	0.6473	-	-
opt_dd49	0	1.6	201	0.6963	201	0.6755	-	-
opt_dd87	0	3.7	202	0.7018	202	0.6818	-	-
opt_cd86	0	3.6	203	0.7497	203	0.7344	16	0.3839
opt_aj30	0	3.1	204	0.7868	204	0.7652	-	-
opt_ak38	0	0.8	205	0.8108	205	0.7938	-	-
opt_ak15	0	3.8	206	0.8238	206	0.8076	6	0.3011
opt_ak45	0	2.6	207	0.8384	207	0.8190	4	0.2970
opt_ak44	0	2.9	208	0.8450	208	0.8307	20	0.3922
opt_de95	0	1.1	209	0.8482	209	0.8336	-	-
opt_ak16	0	4.6	210	0.8595	212	0.8468	2	0.2356
opt_ai46	0	0	211	0.8603	210	0.8409	-	-
opt_aj39	0	0.7	212	0.8612	211	0.8414	-	-

Table S10: Summary of data for caffeine. Relative enthalpy is given in kJ/mol, ranks are according to the next-right column, autoFIDEL comparisons were made with the default program parameters and the experimental powder diffractograms collected over 3 hrs.

structure	duplicates	ΔH	rank	VC-xPWDF (3h)	rank	VC-xPWDF (2m)	rank	autoFIDEL
NIWFEE03	-	-	1	0.0114	1	0.0128	1	0.0601
dfA44	0	7.5	2	0.0220	2	0.0249	5	0.1255
dfA149	0	6.7	3	0.0241	3	0.0264	-	-
dfA73	0	0.5	4	0.0267	4	0.0293	-	-
dfA39	0	11.2	5	0.0347	6	0.0385	-	-
dfA12	0	8.6	6	0.0352	5	0.0377	3	0.0962
dfA142	3	3.6	7	0.0442	7	0.0457	-	-
dfA319	0	11.3	8	0.0754	8	0.0779	20	0.4986
dfA37	0	10.6	9	0.0810	9	0.0835	17	0.3728
dfA35	0	7.5	10	0.1029	10	0.1027	-	-
dfA41	1	8.2	11	0.1371	11	0.1361	-	-
dfA7	0	4.7	12	0.1459	12	0.1403	-	-
dfA336	0	8.4	13	0.1565	13	0.1531	-	-
dfA133	0	6.8	14	0.1726	16	0.1736	-	-
dfA70	0	7.9	15	0.1746	14	0.1715	-	-
dfA22	0	6.5	16	0.1776	15	0.1723	12	0.2373
dfA151	0	4	17	0.1796	17	0.1739	-	-
dfA3	0	0	18	0.1817	18	0.1758	-	-
dfA14	0	1.3	19	0.1934	19	0.1866	14	0.2625
dfA1266	0	9.7	20	0.1952	20	0.1916	-	-
dfA81	0	9	21	0.1979	21	0.1925	7	0.1485
dfA19	0	1.9	22	0.2114	22	0.2057	-	-
dfA92	0	11.2	23	0.2142	24	0.2111	-	-
dfA354	0	9.6	24	0.2172	23	0.2108	-	-
dfA295	0	11.3	25	0.2184	25	0.2126	-	-
dfA318	0	12.6	26	0.2349	27	0.2299	-	-
dfA177	0	7.7	27	0.2355	26	0.2290	6	0.1428
dfA88	0	10.1	28	0.2390	28	0.2325	-	-
dfA104	0	7.6	29	0.2398	29	0.2331	8	0.1593
dfA239	0	7.9	30	0.2454	30	0.2391	18	0.4050
dfA30	0	8.3	31	0.2509	31	0.2454	-	-
dfA275	0	7.3	32	0.2564	32	0.2492	15	0.3080
dfA137	0	10	33	0.2610	33	0.2541	-	-
dfA109	0	9.5	34	0.2678	34	0.2622	-	-
dfA205	0	0.2	35	0.2680	35	0.2625	19	0.4529
dfA1367	0	7.4	36	0.2706	36	0.2667	-	-
dfA144	0	7.6	37	0.2720	37	0.2670	-	-
dfA1261	0	6.4	38	0.2736	39	0.2690	-	-
dfA182	0	10.7	39	0.2766	40	0.2725	-	-
dfA1	1	4.3	40	0.2788	38	0.2686	11	0.2303
dfA57	0	11.5	41	0.2901	41	0.2795	-	-

continued. . .

structure	duplicates	ΔH	rank	VC-xPWDF (3h)	rank	VC-xPWDF (2m)	rank	autoFIDEL
dfA95	0	9.2	42	0.3003	42	0.2963	-	-
dfA67	0	5.9	43	0.3105	43	0.3025	-	-
dfA227	0	12.6	44	0.3109	44	0.3048	-	-
dfA651	0	13.2	45	0.3215	45	0.3159	-	-
dfA199	0	6.8	46	0.3548	46	0.3466	4	0.1049
dfA16	0	4.2	47	0.3698	47	0.3609	-	-
dfA584	0	6	48	0.3703	48	0.3622	-	-
dfA25	0	3.8	49	0.3719	49	0.3640	-	-
dfA129	0	6.9	50	0.3764	50	0.3687	-	-
dfA132	0	14	51	0.3795	51	0.3707	-	-
dfA155	0	9.9	52	0.4136	52	0.3964	-	-
dfA347	0	9.4	53	0.4144	53	0.4053	-	-
dfA54	0	7.3	54	0.4162	54	0.4083	-	-
dfA102	0	5.6	55	0.4167	55	0.4087	10	0.2201
dfA200	0	12.3	56	0.4314	56	0.4213	-	-
dfA80	0	4.3	57	0.4338	57	0.4252	2	0.0942
dfA139	0	7.2	58	0.4383	58	0.4299	-	-
dfA5	0	8	59	0.4406	59	0.4305	9	0.1780
dfA51	0	6.9	60	0.4579	61	0.4498	-	-
dfA218	0	11	61	0.4585	60	0.4486	-	-
dfA219	0	7.3	62	0.4639	62	0.4514	16	0.3668
dfA1759	0	6.7	63	0.4700	63	0.4615	-	-
dfA596	0	11.9	64	0.4758	64	0.4666	-	-
dfA43	0	11.2	65	0.4976	65	0.4855	-	-
dfA3039	0	8.3	66	0.5060	66	0.4936	-	-
dfA72	0	12.6	67	0.5068	67	0.4952	-	-
dfA247	0	12.5	68	0.5091	68	0.4996	21	0.6543
dfA119	0	12.9	69	0.5352	69	0.5237	-	-
dfA126	0	11	70	0.5390	70	0.5266	-	-
dfA98	0	11	71	0.5633	71	0.5507	-	-
dfA48	0	9.7	72	0.5824	72	0.5696	-	-
dfA112	0	11.4	73	0.5913	73	0.5788	-	-
dfA56	0	8.8	74	0.5949	74	0.5819	-	-
dfA213	0	11.1	75	0.6127	75	0.5940	-	-
dfA451	0	5.8	76	0.8406	76	0.8233	13	0.2551
dfA120	0	5.8	77	0.8713	77	0.8552	-	-
dfA172	0	6.4	78	0.8827	78	0.8693	-	-
dfA250	0	11.8	79	0.8843	79	0.8709	-	-
dfA361	0	9.6	80	0.9000	80	0.8863	-	-

Table S11: Summary of data for acetaminophen. Relative enthalpy is given in kJ/mol, ranks are according to the next-right column, autoFIDEL comparisons were made with the default program parameters and the experimental powder diffractograms collected over 3 hrs.

structure	duplicates	ΔH	rank	VC-xPWDF (3h)	rank	VC-xPWDF (2m)	rank	autoFIDEL
HXACAN35	-	-	1	0.0494	1	0.0538	-	-
HXACAN04	-	-	2	0.0602	2	0.0639	-	-
HXACAN15	-	-	3	0.0634	3	0.0666	-	-
HXACAN13	-	-	4	0.0670	4	0.0702	-	-
dfA79	1	0	5	0.0709	5	0.0717	22	0.4398
HXACAN09	-	-	6	0.0772	6	0.0772	-	-
dfA2962	0	11.2	10	0.1256	10	0.1208	-	-
dfA12804	0	9.7	11	0.1289	12	0.1265	-	-
dfA12134	0	8.1	12	0.1294	13	0.1270	16	0.4224
dfA19404	0	8.3	13	0.1346	11	0.1265	-	-
dfA2934	0	8.2	14	0.1362	14	0.1281	-	-
dfA14108	0	11.9	15	0.1454	18	0.1532	-	-
dfA12397	0	4.4	16	0.1529	15	0.1476	-	-
dfA14478	0	12.6	17	0.1531	16	0.1493	-	-
dfA6573	0	11.7	18	0.1575	17	0.1511	20	0.4324
dfA301	0	6.3	19	0.1604	20	0.1598	36	0.4963
dfA8230	0	11.8	20	0.1619	19	0.1547	-	-
dfA857	0	11.5	21	0.1623	21	0.1619	-	-
dfA7552	0	14.2	22	0.1795	24	0.1777	-	-
dfA11586	0	6.8	23	0.1797	22	0.1721	-	-
dfA12281	0	11.9	24	0.1823	31	0.1876	-	-
dfA16671	0	12.7	25	0.1833	25	0.1790	-	-
dfA26324	0	11.9	26	0.1833	32	0.1887	-	-
dfA3516	1	12.3	27	0.1854	23	0.1771	34	0.4885
dfA98	0	4.1	28	0.1862	29	0.1860	-	-
dfA9661	0	12.5	29	0.1872	27	0.1805	-	-
dfA23940	0	12.3	30	0.1875	26	0.1794	-	-
dfA19074	0	14.6	31	0.1902	30	0.1866	-	-
dfA2896	0	9.4	32	0.1903	34	0.1893	-	-
dfA15951	0	13.9	33	0.1920	36	0.1906	-	-
dfA21868	0	14.8	34	0.1942	51	0.2006	-	-
dfA4264	0	9.3	35	0.1943	33	0.1891	-	-
dfA6958	0	12.4	36	0.1952	43	0.1955	-	-
dfA392	0	6.2	37	0.1962	35	0.1895	-	-
dfA1437	0	9.8	38	0.1964	40	0.1947	-	-
dfA15830	0	14.8	39	0.1966	53	0.2024	-	-
dfA1158	0	10.7	40	0.1986	28	0.1852	65	0.6121
dfA13319	0	14.8	41	0.1988	37	0.1927	-	-
dfA16964	0	6.5	42	0.1998	41	0.1954	-	-
dfA17092	0	14.3	43	0.2002	44	0.1962	-	-
dfA3464	0	11.1	44	0.2004	39	0.1945	-	-

continued…

structure	duplicates	ΔH	rank	VC-xPWDF (3h)	rank	VC-xPWDF (2m)	rank	autoFIDEL
dfA4938	0	8.7	45	0.2013	48	0.2000	-	-
dfA3569	0	13.9	46	0.2014	50	0.2004	-	-
dfA11977	0	11.2	47	0.2014	46	0.1976	-	-
dfA14902	0	12.2	48	0.2033	38	0.1935	-	-
dfA390	0	5.8	49	0.2037	52	0.2012	25	0.4622
dfA3141	1	12.3	50	0.2042	42	0.1954	-	-
dfA9748	0	6.6	51	0.2046	45	0.1964	-	-
dfA12913	0	11	52	0.2061	59	0.2065	-	-
dfA4546	0	10.2	53	0.2066	58	0.2054	-	-
dfA13249	0	11	54	0.2079	64	0.2076	-	-
dfA21594	0	14.9	55	0.2082	57	0.2048	-	-
dfA851	0	7.2	56	0.2083	49	0.2000	-	-
dfA25032	0	12.5	57	0.2085	56	0.2042	-	-
dfA14868	0	14.4	58	0.2089	47	0.1995	-	-
dfA1563	0	7.5	59	0.2090	54	0.2026	-	-
dfA10679	0	11.6	60	0.2093	55	0.2040	6	0.3534
dfA19200	0	14.8	61	0.2095	61	0.2069	-	-
dfA6581	0	11.1	62	0.2095	62	0.2073	-	-
dfA24157	0	14.1	63	0.2139	74	0.2193	-	-
dfA27154	0	13	64	0.2139	69	0.2143	8	0.3668
dfA9399	0	5.1	65	0.2143	67	0.2131	-	-
dfA24215	0	8.6	66	0.2152	65	0.2089	-	-
dfA19099	0	13.8	67	0.2159	73	0.2187	-	-
dfA19438	0	11.6	68	0.2160	63	0.2073	-	-
dfA3833	0	9	69	0.2185	68	0.2132	-	-
dfA9414	0	4.5	70	0.2190	71	0.2168	-	-
dfA21088	0	10.1	71	0.2198	66	0.2114	-	-
dfA18942	0	13.6	72	0.2199	72	0.2176	-	-
dfA410	0	5.4	73	0.2199	60	0.2068	-	-
dfA1583	0	8.6	74	0.2222	70	0.2156	-	-
dfA17036	0	6.1	75	0.2236	79	0.2218	-	-
dfA445	0	11.4	76	0.2245	85	0.2252	-	-
dfA15161	0	7.7	77	0.2259	76	0.2213	-	-
dfA1933	0	9.2	78	0.2266	82	0.2237	-	-
dfA5138	0	12.5	79	0.2273	83	0.2241	-	-
dfA1807	0	8.7	80	0.2283	77	0.2215	-	-
dfA15043	0	14.7	81	0.2295	84	0.2243	-	-
dfA3397	0	5.4	82	0.2296	88	0.2282	-	-
dfA905	0	6.8	83	0.2296	91	0.2305	-	-
dfA2548	0	10.4	84	0.2301	80	0.2236	18	0.4296
dfA4314	0	9.5	85	0.2305	75	0.2208	-	-
dfA4304	0	11.4	86	0.2309	78	0.2216	-	-
dfA14749	0	13.6	87	0.2332	92	0.2308	-	-
dfA24221	0	14.9	88	0.2333	81	0.2237	-	-

continued…

structure	duplicates	ΔH	rank	VC-xPWDF (3h)	rank	VC-xPWDF (2m)	rank	autoFIDEL
dfA15679	0	7.7	89	0.2336	87	0.2280	-	-
dfA1229	0	7.6	90	0.2337	101	0.2353	-	-
dfA9036	0	12.4	91	0.2357	90	0.2299	-	-
dfA16478	0	14.5	92	0.2362	86	0.2279	-	-
dfA4451	0	10	93	0.2374	89	0.2295	-	-
dfA14382	0	6.2	94	0.2381	94	0.2321	-	-
dfA25186	0	12.9	95	0.2387	103	0.2361	-	-
dfA960	0	8.9	96	0.2398	97	0.2352	-	-
dfA1777	0	11	97	0.2408	104	0.2372	-	-
dfA3103	0	7.1	98	0.2410	96	0.2345	2	0.3240
dfA15171	0	14.1	99	0.2421	115	0.2407	-	-
dfA21070	0	8.7	100	0.2422	93	0.2312	-	-
dfA4978	0	6	101	0.2426	123	0.2465	-	-
dfA2240	0	9.3	102	0.2429	95	0.2345	-	-
dfA3966	0	11.8	103	0.2433	114	0.2406	-	-
dfA7239	0	7.7	104	0.2439	107	0.2386	-	-
dfA2705	0	8	105	0.2447	105	0.2379	1	0.3229
dfA21529	0	7.1	106	0.2454	108	0.2389	-	-
dfA2761	0	13.9	107	0.2455	121	0.2456	-	-
dfA7836	0	13.8	108	0.2457	99	0.2353	-	-
dfA9355	0	14.7	109	0.2457	116	0.2423	21	0.4366
dfA5411	0	9.3	110	0.2459	120	0.2452	7	0.3620
dfA3266	0	11.1	111	0.2461	98	0.2352	-	-
dfA14265	0	9.8	112	0.2463	102	0.2358	-	-
dfA5402	0	13.2	113	0.2471	111	0.2398	-	-
dfA12187	0	13.6	114	0.2483	106	0.2383	-	-
dfA4000	0	14.1	115	0.2486	100	0.2353	46	0.5364
dfA1365	0	10.2	116	0.2486	113	0.2404	-	-
dfA8259	0	11.5	117	0.2492	109	0.2394	-	-
dfA10708	0	12.5	118	0.2505	122	0.2459	-	-
dfA16648	0	12.9	119	0.2519	117	0.2434	-	-
dfA794	0	6.6	120	0.2530	137	0.2528	-	-
dfA5558	0	9	121	0.2533	112	0.2399	-	-
dfA1928	0	8.2	122	0.2539	126	0.2481	-	-
dfA12111	0	11.1	123	0.2541	119	0.2445	-	-
dfA405	0	6.2	124	0.2542	118	0.2445	-	-
dfA6986	0	13.4	125	0.2545	125	0.2470	-	-
dfA75	0	3.4	126	0.2551	124	0.2470	-	-
dfA10723	0	13.3	127	0.2553	141	0.2541	-	-
dfA3236	0	13.2	128	0.2554	110	0.2397	-	-
dfA20626	0	13.4	129	0.2556	142	0.2541	-	-
dfA14000	0	13.1	130	0.2557	128	0.2493	-	-
dfA3994	0	14.3	131	0.2560	146	0.2554	-	-
dfA13971	0	13.4	132	0.2582	136	0.2523	-	-

continued...

structure	duplicates	ΔH	rank	VC-xPWDF (3h)	rank	VC-xPWDF (2m)	rank	autoFIDEL
dfA6286	0	11	133	0.2584	140	0.2540	-	-
dfA11309	0	14	134	0.2585	129	0.2504	-	-
dfA3712	0	6.8	135	0.2592	147	0.2555	-	-
dfA898	0	7.5	136	0.2595	160	0.2615	58	0.5773
dfA2015	0	9.1	137	0.2596	127	0.2488	-	-
dfA1054	0	8.1	138	0.2599	163	0.2631	-	-
dfA3508	0	12.3	139	0.2599	131	0.2511	-	-
dfA14986	0	14.7	140	0.2601	130	0.2508	-	-
dfA22959	0	13.6	141	0.2602	148	0.2574	-	-
dfA3762	0	10.9	142	0.2603	145	0.2548	-	-
dfA3724	0	14.7	143	0.2605	155	0.2605	-	-
dfA10885	0	11	144	0.2606	161	0.2618	-	-
dfA21798	0	12.9	145	0.2609	139	0.2533	-	-
dfA1629	0	8.1	146	0.2610	134	0.2519	-	-
dfA6073	0	12	147	0.2612	135	0.2523	-	-
dfA2805	0	11.4	148	0.2618	132	0.2512	-	-
dfA809	0	7.1	149	0.2619	143	0.2546	-	-
dfA423	0	6.3	150	0.2627	133	0.2518	-	-
dfA3858	0	13.5	151	0.2631	158	0.2612	13	0.3995
dfA2363	0	8.1	152	0.2633	144	0.2548	-	-
dfA2825	0	10.8	153	0.2645	173	0.2651	-	-
dfA18809	0	13.3	154	0.2649	184	0.2706	-	-
dfA16168	0	12.6	155	0.2649	149	0.2580	-	-
dfA15237	0	2.5	156	0.2650	164	0.2638	47	0.5386
dfA13930	0	8.3	157	0.2656	168	0.2642	-	-
dfA2907	0	13.8	158	0.2661	174	0.2660	-	-
dfA26353	0	11.6	159	0.2663	138	0.2530	-	-
dfA4549	0	12.8	160	0.2665	180	0.2676	-	-
dfA11209	0	12.6	161	0.2674	167	0.2641	-	-
dfA7682	0	11.2	162	0.2677	150	0.2581	-	-
dfA2162	0	8.5	163	0.2678	178	0.2667	-	-
dfA6880	0	9.6	164	0.2679	151	0.2585	-	-
dfA13115	0	14.4	165	0.2679	159	0.2614	-	-
dfA4675	0	11.6	166	0.2685	170	0.2648	-	-
dfA13264	0	13	167	0.2686	165	0.2639	-	-
dfA8802	0	14.2	168	0.2691	169	0.2646	-	-
dfA3775	0	10.1	169	0.2691	157	0.2611	-	-
dfA12925	0	14.4	170	0.2693	172	0.2651	67	0.6192
dfA22485	0	13.7	171	0.2698	156	0.2608	-	-
dfA10027	0	10.4	172	0.2698	162	0.2622	-	-
dfA6828	0	14.8	173	0.2705	152	0.2589	-	-
dfA19544	0	11.6	174	0.2707	197	0.2775	-	-
dfA426	0	7.9	175	0.2711	171	0.2651	-	-
dfA5087	0	9.4	176	0.2713	181	0.2687	35	0.4891

continued...

structure	duplicates	ΔH	rank	VC-xPWDF (3h)	rank	VC-xPWDF (2m)	rank	autoFIDEL
dfA15003	0	14.6	177	0.2720	185	0.2706	-	-
dfA19884	0	12.6	178	0.2727	179	0.2676	-	-
dfA21535	0	10.2	179	0.2728	176	0.2664	-	-
dfA5269	0	9.7	180	0.2734	153	0.2589	50	0.5509
dfA3084	0	8.9	181	0.2745	175	0.2661	-	-
dfA16280	1	5.5	182	0.2745	166	0.2641	32	0.4829
dfA3623	0	10.9	183	0.2772	177	0.2665	-	-
dfA7211	0	7.2	184	0.2779	204	0.2811	-	-
dfA2922	0	6.3	185	0.2779	188	0.2719	-	-
dfA371	0	6.3	186	0.2781	154	0.2599	-	-
dfA8901	0	15	187	0.2789	200	0.2781	27	0.4732
dfA15311	0	9.7	188	0.2798	190	0.2733	-	-
dfA9959	0	14	189	0.2801	192	0.2742	-	-
dfA24848	0	12.8	190	0.2812	191	0.2742	-	-
dfA46	1	0.3	191	0.2817	183	0.2695	-	-
dfA10615	0	11.8	192	0.2826	198	0.2776	-	-
dfA3934	0	11.1	193	0.2827	194	0.2755	-	-
dfA15359	0	13.6	194	0.2828	189	0.2729	54	0.5640
dfA8480	0	13.5	195	0.2830	205	0.2815	-	-
dfA2488	0	14.3	196	0.2832	186	0.2713	-	-
dfA8201	0	12.2	197	0.2835	193	0.2747	-	-
dfA9538	0	13.8	198	0.2838	211	0.2850	-	-
dfA8136	0	10.7	199	0.2842	195	0.2768	-	-
dfA1187	1	8.9	200	0.2854	196	0.2769	-	-
dfA23731	1	12	201	0.2857	216	0.2874	31	0.4792
dfA2815	0	0.7	202	0.2871	202	0.2795	-	-
dfA13922	0	11.6	203	0.2874	201	0.2788	-	-
dfA16157	0	7.4	204	0.2877	224	0.2902	-	-
dfA13861	0	13.1	205	0.2878	182	0.2691	-	-
dfA1870	0	8.3	206	0.2902	187	0.2719	-	-
dfA829	0	7.3	207	0.2907	203	0.2809	-	-
dfA1851	0	11.1	208	0.2911	220	0.2886	-	-
dfA13329	0	11.2	209	0.2917	199	0.2777	-	-
dfA15445	0	9.4	210	0.2921	210	0.2849	-	-
dfA5290	0	7.2	211	0.2921	208	0.2841	-	-
dfA15368	0	14.6	212	0.2936	207	0.2829	-	-
dfA3455	0	11.1	213	0.2941	218	0.2883	-	-
dfA74	0	3.5	214	0.2943	212	0.2854	-	-
dfA7656	0	14.3	215	0.2949	217	0.2876	-	-
dfA739	0	6.4	216	0.2955	223	0.2899	-	-
dfA2936	0	9	217	0.2956	209	0.2847	-	-
dfA20156	0	13.5	218	0.2969	206	0.2821	-	-
dfA19469	0	7.7	219	0.2970	215	0.2865	-	-
dfA714	0	6.3	220	0.2973	226	0.2911	5	0.3494

continued...

structure	duplicates	ΔH	rank	VC-xPWDF (3h)	rank	VC-xPWDF (2m)	rank	autoFIDEL
dfA3899	0	5.3	221	0.2980	238	0.3007	-	-
dfA13542	0	14.2	222	0.2980	214	0.2860	-	-
dfA26472	0	14.1	223	0.2986	221	0.2893	-	-
dfA21196	0	8.8	224	0.2987	227	0.2911	-	-
dfA5141	0	14.5	225	0.2989	232	0.2986	-	-
dfA18835	0	14.7	226	0.2995	233	0.2990	-	-
dfA612	0	7.9	227	0.2997	229	0.2942	-	-
dfA26414	0	11	228	0.3007	222	0.2893	-	-
dfA16249	0	14.7	229	0.3019	225	0.2904	-	-
dfA4403	0	12.2	230	0.3020	228	0.2937	-	-
dfA4168	0	1.8	231	0.3029	219	0.2884	-	-
dfA1284	0	8.1	232	0.3034	244	0.3033	-	-
dfA16410	0	14.9	233	0.3041	234	0.2995	-	-
dfA5906	0	7.7	234	0.3049	231	0.2983	-	-
dfA25497	0	14.7	235	0.3051	252	0.3066	-	-
dfA13572	0	13.2	236	0.3067	213	0.2857	-	-
dfA3157	0	11.6	237	0.3070	243	0.3029	-	-
dfA4030	0	11.9	238	0.3079	237	0.3006	-	-
dfA634	0	11.8	239	0.3084	230	0.2965	-	-
dfA5448	0	12.2	240	0.3092	242	0.3025	-	-
dfA26287	0	14.1	241	0.3099	236	0.3005	-	-
dfA9927	1	7.3	242	0.3102	273	0.3148	-	-
dfA1024	1	6.2	243	0.3103	276	0.3158	-	-
dfA16403	0	10.6	244	0.3105	246	0.3039	-	-
dfA8019	0	12.6	245	0.3108	248	0.3048	-	-
dfA1289	0	7.2	246	0.3114	279	0.3167	-	-
dfA6657	0	14.4	247	0.3115	249	0.3049	-	-
dfA822	0	5.7	248	0.3118	247	0.3043	-	-
dfA11851	0	12.8	249	0.3119	262	0.3111	-	-
dfA146	0	1.7	250	0.3119	235	0.2999	14	0.4010
dfA8258	0	10.4	251	0.3135	239	0.3012	-	-
dfA142	0	5	252	0.3135	240	0.3018	15	0.4222
dfA305	0	9.2	253	0.3144	251	0.3061	-	-
dfA3595	0	8.5	254	0.3145	278	0.3166	-	-
dfA1471	1	10.1	255	0.3152	253	0.3076	-	-
dfA15590	0	9.6	256	0.3154	255	0.3085	-	-
dfA216	0	6.1	257	0.3155	256	0.3089	-	-
dfA9705	0	12.3	258	0.3171	283	0.3181	-	-
dfA20503	0	12.4	259	0.3173	245	0.3039	-	-
dfA2187	0	7	260	0.3176	258	0.3098	44	0.5346
dfA3982	0	13.1	261	0.3178	267	0.3135	-	-
dfA1035	0	8.4	262	0.3186	241	0.3024	-	-
dfA14637	0	14.7	263	0.3191	264	0.3122	-	-
dfA1628	0	7.7	264	0.3192	265	0.3128	-	-

continued…

structure	duplicates	ΔH	rank	VC-xPWDF (3h)	rank	VC-xPWDF (2m)	rank	autoFIDEL
dfA6227	0	14.8	265	0.3193	272	0.3145	-	-
dfA2708	0	5	266	0.3201	289	0.3194	-	-
dfA9781	0	14.8	267	0.3207	259	0.3109	-	-
dfA3948	0	10.4	268	0.3207	261	0.3109	-	-
dfA1648	0	12.7	269	0.3215	274	0.3151	-	-
dfA2001	0	14.5	270	0.3223	286	0.3191	-	-
dfA5414	0	12	271	0.3223	271	0.3141	-	-
dfA147	0	1.7	272	0.3223	260	0.3109	11	0.3940
dfA10751	0	11.1	273	0.3226	250	0.3060	-	-
dfA652	0	5.9	274	0.3237	257	0.3091	-	-
dfA1157	0	8.8	275	0.3239	280	0.3175	-	-
dfA13491	0	13.6	276	0.3239	254	0.3081	-	-
dfA15752	0	11	277	0.3240	269	0.3141	-	-
dfA1770	0	10.1	278	0.3250	314	0.3309	-	-
dfA13085	0	14.2	279	0.3252	277	0.3164	-	-
dfA14535	0	11.9	280	0.3253	294	0.3226	-	-
dfA26909	0	11.6	281	0.3260	293	0.3217	-	-
dfA2989	0	11.2	282	0.3274	284	0.3182	-	-
dfA7050	0	11.5	283	0.3274	281	0.3178	-	-
dfA3831	1	11	284	0.3278	290	0.3199	-	-
dfA3861	0	8.2	285	0.3282	275	0.3158	-	-
dfA4569	0	1.8	286	0.3283	266	0.3128	-	-
dfA10874	0	13.7	287	0.3285	282	0.3180	-	-
dfA11548	0	13.8	288	0.3291	291	0.3209	-	-
dfA2991	0	7.6	289	0.3292	285	0.3183	-	-
dfA3282	0	7.9	290	0.3301	263	0.3116	-	-
dfA6831	0	2.5	291	0.3304	270	0.3141	-	-
dfA26137	0	10.8	292	0.3311	297	0.3245	-	-
dfA12195	0	10.9	293	0.3314	268	0.3140	-	-
dfA384	0	6.6	294	0.3316	295	0.3229	52	0.5604
dfA2766	0	6.8	295	0.3321	317	0.3321	-	-
dfA6875	0	14.6	296	0.3322	299	0.3247	-	-
dfA14583	0	13.8	297	0.3332	316	0.3316	-	-
dfA7467	0	7.7	298	0.3336	288	0.3193	-	-
dfA16960	0	12.7	299	0.3340	287	0.3191	-	-
dfA6964	0	13.5	300	0.3343	292	0.3216	-	-
dfA1198	0	8.1	301	0.3346	298	0.3247	-	-
dfA2173	0	9.1	302	0.3360	302	0.3271	-	-
dfA1092	0	8.9	303	0.3365	312	0.3295	12	0.3948
dfA13648	0	7.3	304	0.3372	311	0.3289	-	-
dfA7814	0	12.2	305	0.3375	310	0.3289	-	-
dfA3329	0	13.7	306	0.3376	322	0.3337	-	-
dfA12120	0	11.9	307	0.3377	296	0.3240	-	-
dfA7982	0	11.4	308	0.3379	305	0.3278	-	-

continued. . .

structure	duplicates	ΔH	rank	VC-xPWDF (3h)	rank	VC-xPWDF (2m)	rank	autoFIDEL
dfA2406	0	8.6	309	0.3379	306	0.3278	60	0.5868
dfA2402	0	8.7	310	0.3385	303	0.3276	-	-
dfA9303	0	13.3	311	0.3391	313	0.3300	-	-
dfA1741	0	11	312	0.3395	304	0.3277	-	-
dfA10623	0	12.2	313	0.3400	343	0.3455	-	-
dfA3791	0	2.4	314	0.3406	308	0.3282	-	-
dfA10012	0	13.3	315	0.3409	307	0.3279	-	-
dfA4001	0	12.4	316	0.3418	320	0.3332	-	-
dfA10331	0	11.2	317	0.3423	301	0.3257	-	-
dfA15482	0	10.7	318	0.3424	327	0.3386	-	-
dfA12386	0	11.4	319	0.3426	309	0.3287	29	0.4773
dfA14804	0	2.4	320	0.3433	323	0.3342	55	0.5729
dfA14310	0	14.8	321	0.3434	300	0.3256	-	-
dfA1663	0	7.5	322	0.3438	330	0.3393	-	-
dfA1528	0	8.3	323	0.3450	329	0.3390	-	-
dfA13623	0	8.9	324	0.3456	319	0.3331	-	-
dfA1269	0	7.2	325	0.3472	335	0.3421	-	-
dfA589	0	6.5	326	0.3473	336	0.3431	-	-
dfA7375	0	14.6	327	0.3482	315	0.3313	-	-
dfA3743	0	7.4	328	0.3482	318	0.3328	-	-
dfA736	0	4.3	329	0.3484	321	0.3334	-	-
dfA18197	0	1.6	330	0.3495	328	0.3386	-	-
dfA529	0	5.8	331	0.3496	331	0.3395	24	0.4584
dfA8076	0	13	332	0.3496	325	0.3368	-	-
dfA24195	0	14.4	333	0.3503	346	0.3464	-	-
dfA724	0	4.9	334	0.3505	353	0.3493	-	-
dfA13947	0	13.9	335	0.3517	326	0.3371	-	-
dfA415	0	6.7	336	0.3518	340	0.3453	-	-
dfA3116	0	14.7	337	0.3523	324	0.3356	-	-
dfA3870	0	12	338	0.3527	333	0.3415	-	-
dfA8713	0	13.8	339	0.3532	352	0.3487	-	-
dfA9116	0	10.7	340	0.3545	338	0.3443	-	-
dfA1309	0	4.9	341	0.3547	350	0.3478	26	0.4670
dfA5565	0	13.8	342	0.3548	347	0.3464	-	-
dfA13351	0	13.2	343	0.3550	339	0.3443	-	-
dfA12435	0	13.2	344	0.3558	345	0.3460	-	-
dfA3472	0	14.4	345	0.3559	358	0.3511	-	-
dfA4233	0	11.5	346	0.3567	348	0.3466	-	-
dfA3692	0	12.3	347	0.3573	351	0.3483	-	-
dfA5361	0	9.8	348	0.3573	344	0.3458	-	-
dfA2081	0	10.8	349	0.3574	354	0.3504	-	-
dfA94	0	1.5	350	0.3575	332	0.3397	40	0.5131
dfA8286	0	7.3	351	0.3576	334	0.3420	-	-
dfA14490	0	13.6	352	0.3584	337	0.3436	-	-

continued...

structure	duplicates	ΔH	rank	VC-xPWDF (3h)	rank	VC-xPWDF (2m)	rank	autoFIDEL
dfA8660	0	12.6	353	0.3585	361	0.3539	-	-
dfA7389	0	11.1	354	0.3587	341	0.3453	-	-
dfA14390	0	14.3	355	0.3597	357	0.3510	30	0.4780
dfA1749	2	8.9	356	0.3605	356	0.3506	-	-
dfA4952	0	12.3	357	0.3608	349	0.3476	-	-
dfA23421	0	13.6	358	0.3611	342	0.3455	-	-
dfA22478	0	12.9	359	0.3621	359	0.3513	-	-
dfA7502	0	9.5	360	0.3627	363	0.3552	-	-
dfA1572	0	11.2	361	0.3630	355	0.3505	-	-
dfA19581	0	12.4	362	0.3663	360	0.3532	-	-
dfA1771	0	8	363	0.3674	366	0.3583	-	-
dfA6533	0	14.3	364	0.3676	373	0.3625	-	-
dfE42	1	11.3	365	0.3678	362	0.3544	-	-
dfA11629	0	14.7	366	0.3685	367	0.3588	63	0.6051
dfA9495	0	13.2	367	0.3702	372	0.3619	-	-
dfA7171	0	14.4	368	0.3742	368	0.3603	-	-
dfA4086	0	7.6	369	0.3750	374	0.3631	-	-
dfA27003	0	10.4	370	0.3750	375	0.3636	-	-
dfA8232	0	10.7	371	0.3751	377	0.3647	4	0.3473
dfA12722	0	11.5	372	0.3757	393	0.3770	-	-
dfA8766	0	14.1	373	0.3758	376	0.3643	-	-
dfA37	0	2.6	374	0.3762	371	0.3614	-	-
dfA17660	0	13.5	375	0.3767	379	0.3671	-	-
dfA13554	0	14.3	376	0.3768	370	0.3610	-	-
dfA8933	0	12.3	377	0.3770	383	0.3688	45	0.5363
dfA12886	0	14	378	0.3771	397	0.3798	-	-
dfA2772	0	5.1	379	0.3771	378	0.3668	-	-
dfA11650	0	11.5	380	0.3774	381	0.3679	-	-
dfA6251	0	14.6	381	0.3775	365	0.3562	-	-
dfA16734	0	10.7	382	0.3776	364	0.3561	-	-
dfA2657	1	12	383	0.3781	388	0.3714	-	-
dfA15919	0	12.4	384	0.3785	369	0.3607	64	0.6070
dfA5493	0	2.7	385	0.3789	392	0.3767	38	0.4973
dfA25009	0	6.3	386	0.3796	382	0.3682	-	-
dfA3678	0	11.9	387	0.3800	384	0.3692	-	-
dfA23640	0	5.2	388	0.3814	390	0.3732	-	-
dfA16184	0	8.8	389	0.3815	385	0.3692	56	0.5748
dfA26566	0	10.9	390	0.3819	395	0.3786	-	-
dfA550	0	5.3	391	0.3830	394	0.3785	-	-
dfA3431	0	8.5	392	0.3834	380	0.3679	-	-
dfA13285	0	11.8	393	0.3844	389	0.3732	-	-
dfA3640	0	13.3	394	0.3852	391	0.3762	-	-
dfA802	0	7.5	395	0.3892	387	0.3712	-	-
dfA7313	0	14.8	396	0.3904	398	0.3806	-	-

continued…

structure	duplicates	ΔH	rank	VC-xPWDF (3h)	rank	VC-xPWDF (2m)	rank	autoFIDEL
dfA2726	0	9.9	397	0.3911	386	0.3698	-	-
dfA14215	0	10.3	398	0.3926	406	0.3887	-	-
dfA3074	0	12	399	0.3936	405	0.3885	-	-
dfA9313	0	9.6	400	0.3945	410	0.3901	-	-
dfA14443	0	14	401	0.3952	404	0.3884	-	-
dfA9946	0	12.1	402	0.3953	401	0.3837	-	-
dfA14781	0	7.3	403	0.3956	396	0.3791	-	-
dfA1979	0	7.7	404	0.3964	407	0.3888	-	-
dfA729	0	7.8	405	0.3968	415	0.3932	-	-
dfA8421	0	12.3	406	0.3971	402	0.3861	-	-
dfA20663	0	8.6	407	0.3974	411	0.3913	-	-
dfA3535	0	10.6	408	0.3993	403	0.3880	-	-
dfA17246	0	11.8	409	0.4001	399	0.3814	-	-
dfA10106	0	14.2	410	0.4002	400	0.3815	-	-
dfA12550	0	13.5	411	0.4009	427	0.3999	-	-
dfA3686	0	11	412	0.4010	412	0.3917	-	-
dfA1765	1	4.1	413	0.4013	408	0.3890	-	-
dfA1495	1	8.8	414	0.4015	418	0.3940	-	-
dfA19043	0	13	415	0.4051	417	0.3940	-	-
dfA3556	0	10.3	416	0.4057	446	0.4137	-	-
dfA25387	0	9.9	417	0.4057	419	0.3942	-	-
dfA1192	0	8.1	418	0.4074	422	0.3978	-	-
dfA16559	0	14.6	419	0.4076	414	0.3923	-	-
dfA1896	0	8.7	420	0.4084	416	0.3938	-	-
dfA18606	0	14.1	421	0.4095	433	0.4036	-	-
dfA14981	0	15	422	0.4096	409	0.3891	-	-
HXACAN47	-	-	423	0.4099	423	0.3988	-	-
dfA15652	0	13.1	424	0.4106	413	0.3918	-	-
dfA651	0	7.2	425	0.4107	425	0.3991	-	-
dfA5772	0	6.8	426	0.4107	430	0.4024	33	0.4865
dfA5920	0	10.4	427	0.4124	432	0.4033	-	-
dfA11215	0	13.9	428	0.4126	420	0.3973	-	-
dfA8385	0	10.6	429	0.4136	426	0.3992	-	-
dfA15005	0	14.4	430	0.4141	435	0.4072	-	-
dfA4881	0	11.7	431	0.4142	436	0.4080	-	-
dfA5279	0	11.7	432	0.4145	429	0.4011	-	-
dfA12338	0	10.7	433	0.4146	421	0.3974	3	0.3322
dfA5592	0	4.9	434	0.4164	424	0.3990	-	-
dfA18430	0	5.6	435	0.4166	431	0.4029	9	0.3704
dfA1409	0	8.1	436	0.4168	428	0.4006	-	-
dfA11846	0	13.6	437	0.4175	443	0.4130	-	-
dfA3242	0	7.6	438	0.4203	454	0.4167	-	-
dfA17125	0	8.5	439	0.4209	438	0.4095	-	-
dfA26797	0	8.3	440	0.4209	449	0.4145	-	-

continued. . .

structure	duplicates	ΔH	rank	VC-xPWDF (3h)	rank	VC-xPWDF (2m)	rank	autoFIDEL
dfA25463	0	12.5	441	0.4210	448	0.4142	-	-
dfA19942	0	6.2	442	0.4213	441	0.4125	-	-
dfA2047	0	10.1	443	0.4232	444	0.4131	-	-
dfA12490	0	12.9	444	0.4244	445	0.4134	-	-
dfA6550	0	14.9	445	0.4248	434	0.4065	-	-
dfA1577	0	11.3	446	0.4248	437	0.4087	-	-
dfA18844	0	11.5	447	0.4251	456	0.4189	-	-
dfA2608	0	13.4	448	0.4259	450	0.4146	-	-
dfA4696	0	14.3	449	0.4262	451	0.4155	-	-
dfA5291	0	9.2	450	0.4284	442	0.4129	-	-
dfA2029	0	10	451	0.4285	440	0.4120	-	-
dfA3040	0	7.4	452	0.4288	447	0.4137	-	-
dfA9421	0	10.6	453	0.4298	488	0.4380	-	-
dfA3577	0	12.8	454	0.4299	452	0.4159	-	-
dfA5887	0	14.4	455	0.4300	439	0.4104	-	-
dfA4976	0	9.5	456	0.4309	453	0.4162	69	0.6483
dfA5351	0	10.5	457	0.4313	459	0.4211	-	-
dfA5866	0	14.9	458	0.4313	457	0.4189	-	-
dfA8262	0	13.5	459	0.4322	475	0.4329	-	-
dfA19020	0	13.8	460	0.4324	469	0.4294	66	0.6151
dfA7051	0	7.1	461	0.4326	464	0.4253	61	0.5869
dfA11014	0	15	462	0.4340	458	0.4195	-	-
dfA4615	0	8.8	463	0.4341	468	0.4293	-	-
dfA3996	0	6.6	464	0.4352	463	0.4241	-	-
dfA11866	0	9.9	465	0.4357	467	0.4286	17	0.4237
dfA7923	0	15	466	0.4360	465	0.4256	-	-
dfA20819	0	9.7	467	0.4362	455	0.4177	-	-
dfA7011	0	13.4	468	0.4374	470	0.4295	-	-
dfA27149	0	5.7	469	0.4379	461	0.4235	-	-
dfA3990	0	11.7	470	0.4383	460	0.4217	-	-
dfA12796	0	10.7	471	0.4386	477	0.4336	-	-
dfA1936	0	11.7	472	0.4398	466	0.4256	-	-
dfA17220	0	14.5	473	0.4422	471	0.4304	-	-
dfA6496	0	10.6	474	0.4426	478	0.4340	-	-
dfA3528	0	10.7	475	0.4431	462	0.4239	-	-
dfA6121	0	9.4	476	0.4432	490	0.4391	-	-
dfA4712	0	9.5	477	0.4436	489	0.4390	-	-
dfA13752	0	10.8	478	0.4438	474	0.4323	-	-
dfA20958	0	14.5	479	0.4442	482	0.4356	-	-
dfA2393	0	6.3	480	0.4444	483	0.4362	-	-
dfA13845	0	9.5	481	0.4444	473	0.4323	-	-
dfA1916	0	8.6	482	0.4451	472	0.4312	-	-
dfA733	0	7.4	483	0.4453	480	0.4351	-	-
dfA11068	0	13.6	484	0.4472	481	0.4352	-	-

continued. . .

structure	duplicates	ΔH	rank	VC-xPWDF (3h)	rank	VC-xPWDF (2m)	rank	autoFIDEL
dfA17292	0	6	485	0.4477	476	0.4333	37	0.4967
dfA5359	0	9.3	486	0.4479	494	0.4440	-	-
dfA3952	0	13.2	487	0.4481	486	0.4368	-	-
dfA4709	0	10.2	488	0.4491	509	0.4531	-	-
dfA3252	0	8.3	489	0.4499	496	0.4447	-	-
dfA4093	0	11.4	490	0.4511	484	0.4365	28	0.4761
dfA5135	0	7.3	491	0.4513	491	0.4410	-	-
dfA8783	0	11.8	492	0.4530	497	0.4448	-	-
dfA3249	0	14.1	493	0.4534	479	0.4350	-	-
dfA9137	0	13.4	494	0.4536	498	0.4449	-	-
dfA3849	0	12.1	495	0.4540	499	0.4460	39	0.4994
dfA8556	0	10.7	496	0.4547	506	0.4504	-	-
dfA2510	0	8.7	497	0.4549	487	0.4374	-	-
dfA9113	0	8.7	498	0.4557	493	0.4423	10	0.3782
dfA4830	0	11	499	0.4571	492	0.4414	41	0.5205
dfA1976	0	9	500	0.4572	485	0.4367	-	-
dfA3309	0	6.8	501	0.4589	501	0.4472	-	-
dfA3361	0	9.3	502	0.4591	504	0.4491	-	-
dfA10870	0	14.6	503	0.4616	505	0.4503	-	-
dfA4017	0	10.4	504	0.4622	507	0.4507	-	-
dfA6338	0	14.7	505	0.4622	500	0.4462	57	0.5763
dfA5728	0	12.6	506	0.4623	503	0.4483	-	-
dfA2482	0	13.9	507	0.4632	511	0.4545	-	-
dfA10717	0	13.2	508	0.4637	495	0.4445	-	-
dfA2356	0	10.7	509	0.4639	510	0.4534	-	-
dfA18311	0	13.7	510	0.4641	524	0.4690	-	-
dfA2255	0	10.1	511	0.4655	516	0.4614	-	-
dfA17430	0	11.8	512	0.4681	512	0.4564	-	-
dfA10530	0	10.2	513	0.4684	508	0.4510	-	-
dfA2776	0	9.7	514	0.4685	521	0.4662	-	-
dfA8479	0	14.7	515	0.4687	502	0.4479	68	0.6212
dfA12415	0	10.1	516	0.4692	513	0.4579	-	-
dfA2988	0	11	517	0.4712	517	0.4617	-	-
dfA14180	0	14.3	518	0.4749	514	0.4584	-	-
dfA870	0	8.5	519	0.4751	518	0.4621	-	-
dfA17318	0	9.6	520	0.4753	529	0.4726	-	-
dfA6186	0	8.9	521	0.4760	515	0.4597	-	-
dfA14953	0	8.2	522	0.4779	519	0.4640	-	-
dfA10943	0	4.4	523	0.4806	522	0.4688	-	-
dfA8629	0	13.3	524	0.4810	520	0.4658	-	-
dfA2314	0	8.1	525	0.4825	527	0.4699	49	0.5484
dfA847	0	7.8	526	0.4830	526	0.4696	42	0.5236
dfA3487	0	14	527	0.4843	535	0.4774	-	-
dfA416	0	7.1	528	0.4843	525	0.4691	48	0.5480

continued. . .

structure	duplicates	ΔH	rank	VC-xPWDF (3h)	rank	VC-xPWDF (2m)	rank	autoFIDEL
dfA14701	0	14.2	529	0.4850	531	0.4762	-	-
dfA10318	0	12.8	530	0.4850	523	0.4688	-	-
dfA3548	0	11.6	531	0.4863	528	0.4712	-	-
dfA8297	0	13	532	0.4878	530	0.4742	-	-
dfA1120	0	9.1	533	0.4884	536	0.4784	-	-
dfA11097	0	14.1	534	0.4909	534	0.4772	-	-
dfA4697	0	9.3	535	0.4914	532	0.4763	-	-
dfA13132	0	13.8	536	0.4918	537	0.4806	-	-
dfA2861	0	12.7	537	0.4921	539	0.4814	-	-
dfA2092	1	7.5	538	0.4926	533	0.4769	-	-
dfA10841	0	8.4	539	0.4928	540	0.4836	51	0.5572
dfA23620	0	14.6	540	0.4931	538	0.4811	-	-
dfA12362	0	13.3	541	0.4932	541	0.4840	-	-
dfA25928	0	12	542	0.4947	545	0.4918	43	0.5247
dfA2869	0	11.6	543	0.4962	543	0.4858	-	-
dfA8844	0	12	544	0.4974	542	0.4841	-	-
dfA7152	0	10.1	545	0.5043	546	0.4928	-	-
dfA20001	0	8.7	546	0.5044	544	0.4883	-	-
dfA7651	0	15	547	0.5084	549	0.4992	-	-
dfA3270	0	7.9	548	0.5112	550	0.5002	-	-
dfA11582	0	14.7	549	0.5117	556	0.5154	-	-
dfA26696	0	12.8	550	0.5136	547	0.4965	-	-
dfA826	0	8.5	551	0.5149	548	0.4972	-	-
dfA1200	0	8.1	552	0.5151	552	0.5069	-	-
dfA4489	0	8.6	553	0.5163	551	0.5038	-	-
dfA9109	0	10.9	554	0.5213	553	0.5078	-	-
dfA1099	0	9.1	555	0.5223	555	0.5145	-	-
dfA7915	0	8.4	556	0.5248	554	0.5131	-	-
dfA12754	0	13.3	557	0.5276	565	0.5263	-	-
dfA11788	0	11.8	558	0.5279	558	0.5163	62	0.5919
dfA11055	0	12.9	559	0.5313	573	0.5347	-	-
dfA21086	0	11.7	560	0.5316	560	0.5207	-	-
dfA1342	0	13.8	561	0.5318	564	0.5259	-	-
dfA23256	0	12.2	562	0.5319	567	0.5281	-	-
dfA8508	0	14.5	563	0.5325	557	0.5154	-	-
dfA20949	0	13	564	0.5335	559	0.5205	-	-
dfA14349	0	14.4	565	0.5339	575	0.5369	-	-
dfA4951	0	9	566	0.5375	571	0.5308	-	-
dfA12229	0	13.9	567	0.5381	568	0.5286	-	-
dfA5462	0	12.8	568	0.5382	562	0.5227	59	0.5826
dfA4564	0	10.1	569	0.5382	563	0.5248	-	-
dfA13173	0	9.1	570	0.5388	570	0.5306	-	-
dfA3589	0	10	571	0.5400	561	0.5224	-	-
dfA11471	0	12.4	572	0.5411	566	0.5275	-	-

continued. . .

structure	duplicates	ΔH	rank	VC-xPWDF (3h)	rank	VC-xPWDF (2m)	rank	autoFIDEL
dfA9578	0	12.5	573	0.5420	576	0.5376	-	-
dfA9967	0	12.9	574	0.5441	569	0.5296	-	-
dfA7902	0	12.7	575	0.5442	577	0.5377	-	-
dfA4545	0	9.6	576	0.5444	572	0.5308	-	-
dfA530	0	7.9	577	0.5456	578	0.5401	19	0.4304
dfA13160	0	8.3	578	0.5468	580	0.5409	-	-
dfA5868	0	13.6	579	0.5472	574	0.5349	-	-
dfA14674	0	9.6	580	0.5498	583	0.5443	-	-
dfA12505	0	8.5	581	0.5508	579	0.5403	-	-
dfA1412	0	8.8	582	0.5536	581	0.5410	53	0.5638
dfA3375	0	8.6	583	0.5545	585	0.5486	-	-
dfA25959	0	12.9	584	0.5630	586	0.5508	-	-
dfA2467	0	8.2	585	0.5631	584	0.5473	-	-
dfA2159	0	10	586	0.5634	589	0.5521	-	-
dfA6455	0	13.9	587	0.5641	591	0.5568	-	-
dfA4860	0	8.5	588	0.5651	582	0.5439	-	-
dfA8206	0	11.7	589	0.5683	587	0.5516	-	-
dfA11819	0	14.8	590	0.5683	590	0.5556	-	-
dfA25337	0	10.8	591	0.5706	598	0.5662	-	-
dfA4733	0	11.5	592	0.5716	588	0.5519	-	-
dfE3	1	2.6	593	0.5732	592	0.5574	-	-
dfA2842	0	10.6	594	0.5753	599	0.5676	-	-
dfA2661	0	9.9	595	0.5758	593	0.5585	-	-
dfA8552	0	7.4	596	0.5759	597	0.5662	-	-
HXACAN40	-	-	597	0.5764	596	0.5618	-	-
dfA7472	0	14.6	598	0.5766	595	0.5600	-	-
dfA8143	0	14.1	599	0.5766	600	0.5693	-	-
dfA2860	0	7.2	600	0.5795	594	0.5597	23	0.4559
dfA15668	0	14.8	601	0.5805	603	0.5775	-	-
dfA8301	0	8.4	602	0.5841	602	0.5757	-	-
dfA6433	1	7.7	603	0.5878	601	0.5716	-	-
dfA9499	0	8.8	604	0.5895	605	0.5811	-	-
dfA6443	0	11.1	605	0.5897	606	0.5824	-	-
dfA13568	0	14.8	606	0.5906	607	0.5832	-	-
dfA5244	0	11.3	607	0.5928	609	0.5880	-	-
dfA21905	0	13.3	608	0.5975	604	0.5809	70	0.6504
dfA1914	0	8.6	609	0.5979	608	0.5863	-	-
dfA22709	0	14.1	610	0.6016	610	0.5943	-	-
dfA668	0	7.3	611	0.6022	611	0.5973	-	-
dfA1036	0	7.7	612	0.6064	614	0.6024	-	-
dfA3746	0	10.6	613	0.6088	612	0.5984	-	-
dfA7007	0	11.5	614	0.6118	613	0.6015	-	-
dfA5216	0	11	615	0.6147	615	0.6025	-	-
dfA24973	1	11	616	0.6175	616	0.6053	-	-

continued...

structure	duplicates	ΔH	rank	VC-xPWDF (3h)	rank	VC-xPWDF (2m)	rank	autoFIDEL
dfA5503	0	13.8	617	0.6253	617	0.6081	-	-
dfA12946	0	7.6	618	0.6271	618	0.6103	-	-
dfA9901	0	13.5	619	0.6278	621	0.6324	-	-
dfA2600	0	10.4	620	0.6292	620	0.6244	-	-
dfA11704	0	11.1	621	0.6297	619	0.6177	-	-
dfA1560	0	12	622	0.6554	623	0.6370	-	-
dfA11850	0	14.7	623	0.6558	622	0.6330	-	-
dfA241	1	3.6	624	0.6620	624	0.6464	-	-
dfA9908	0	6	625	0.6709	625	0.6522	-	-
dfA2750	0	12	626	0.7216	626	0.7024	-	-
dfA671	0	8.1	627	0.7260	627	0.7055	-	-
HXACAN33	-	-	628	0.7293	628	0.7139	-	-
dfA2286	0	12.2	629	0.7434	629	0.7247	-	-

Table S12: Summary of data for D-mannitol. Relative enthalpy is given in kJ/mol, ranks are according to the next-right column, autoFIDEL comparisons were made with the default program parameters and the experimental powder diffractograms collected over 3 hrs.

structure	duplicates	ΔH	rank	VC-xPWDF (3h)	rank	VC-xPWDF (2m)	rank	autoFIDEL
DMANTL15	-	-	1	0.0962	1	0.0914	-	-
DMANTL07	-	-	2	0.0992	2	0.0946	1	0.1136
d9_af92	0	80.3	3	0.1573	3	0.1574	6	0.3718
d10_au101	0	101.9	4	0.1756	4	0.1734	-	-
dl_au92	0	98.3	5	0.1771	5	0.1748	-	-
d10_aa58	0	91.3	6	0.1797	6	0.1798	-	-
d9_db59	0	69.8	7	0.1832	7	0.1808	-	-
d9_ah99	0	80.4	8	0.1856	8	0.1827	-	-
dl_aa111	1	86.4	9	0.1897	9	0.1894	-	-
d9_aq27	0	79	10	0.2032	10	0.1969	-	-
d9_ah33	0	81.9	11	0.2126	11	0.2087	-	-
dl_aa79	1	87.7	12	0.2184	12	0.2141	27	0.5903
d10_aa91	1	92.4	13	0.2186	13	0.2147	-	-
d9_ap33	0	80.5	14	0.2292	14	0.2217	-	-
dl_af95	0	90.6	15	0.2310	15	0.2249	-	-
d9_ah129	1	64.4	16	0.2319	17	0.2287	-	-
d9_db92	0	80.1	17	0.2329	16	0.2261	-	-
d10_af10	0	95.9	18	0.2358	18	0.2301	-	-
dl_af12	0	87.8	19	0.2387	19	0.2345	-	-
d9_ap24	0	75	20	0.2430	20	0.2365	-	-
d8_af44	1	29.4	21	0.2502	21	0.2455	-	-
d9_ah19	0	75.7	22	0.2530	22	0.2505	-	-
d10_af49	1	90.4	23	0.2556	24	0.2512	-	-
d9_aq42	0	76.4	24	0.2557	23	0.2508	-	-
dl_ah27	0	95	25	0.2762	25	0.2708	-	-
d9_av23	0	74.2	26	0.2771	27	0.2737	3	0.3058
dl_au68	0	99.7	27	0.2794	26	0.2724	-	-
d9_aa21	0	80.1	28	0.2818	29	0.2754	-	-
d8_au58	0	25.1	29	0.2819	28	0.2742	18	0.5152
d10_af41	0	101.3	30	0.2839	31	0.2792	-	-
dl_ah48	0	94.1	31	0.2851	30	0.2764	-	-
d9_aq55	1	68.8	32	0.2865	32	0.2811	-	-
d9_ay20	0	82.8	33	0.2892	33	0.2826	-	-
d9_cb92	0	77.1	34	0.2902	34	0.2833	-	-
d8_aa5	2	27	35	0.2904	36	0.2846	-	-
d10_ay33	0	94.9	36	0.2916	35	0.2843	-	-
dl_ay44	1	91.1	37	0.2927	37	0.2854	-	-
d10_cb142	0	100.1	38	0.2938	39	0.2900	-	-
d10_aa19	1	82.9	39	0.2971	38	0.2877	-	-
d9_cb138	0	80.3	40	0.2978	40	0.2901	-	-
d10_ah7	0	100.2	41	0.3070	41	0.2977	-	-

continued...

structure	duplicates	ΔH	rank	VC-xPWDF (3h)	rank	VC-xPWDF (2m)	rank	autoFIDEL
d8_as87	0	27.9	42	0.3111	42	0.3043	-	-
d8_av23	0	21.7	43	0.3139	44	0.3082	-	-
DMANTL08	-	-	44	0.3145	43	0.3060	-	-
d9_aq136	0	77.2	45	0.3230	45	0.3127	-	-
dl_ah89	0	90.5	46	0.3242	48	0.3197	-	-
d8_au79	0	23	47	0.3242	46	0.3140	-	-
dl_as76	0	94.8	48	0.3253	47	0.3168	10	0.4402
dl_af33	1	98.4	49	0.3276	49	0.3215	-	-
dl_ah97	0	84.3	50	0.3292	51	0.3225	-	-
dl_au56	1	93.3	51	0.3294	50	0.3221	-	-
dl_ap55	0	104.9	52	0.3311	55	0.3286	-	-
dl_db31	2	96	53	0.3321	54	0.3285	-	-
d8_aq89	1	10.5	54	0.3330	52	0.3232	-	-
d10_ba65	0	88.5	55	0.3358	53	0.3284	-	-
d9_ay85	0	79.3	56	0.3373	58	0.3333	-	-
d9_ah76	0	78.6	57	0.3379	56	0.3310	-	-
d9_ba75	0	75.1	58	0.3389	59	0.3345	-	-
dl_ap52	0	86.1	59	0.3400	57	0.3323	-	-
d10_ah90	0	89	60	0.3400	61	0.3351	-	-
d10_af65	0	101.4	61	0.3420	62	0.3359	-	-
d9_aq73	0	81.5	62	0.3422	60	0.3345	-	-
d10_ah89	0	93.4	63	0.3437	63	0.3367	-	-
d9_cd70	0	77.8	64	0.3449	64	0.3385	-	-
d10_au64	0	99.6	65	0.3476	65	0.3409	-	-
d9_af68	0	71.4	66	0.3479	66	0.3423	-	-
d9_bd96	0	73.2	67	0.3489	68	0.3451	-	-
d9_bh89	0	75.3	68	0.3522	67	0.3433	-	-
d9_ay98	0	82.8	69	0.3545	74	0.3484	-	-
d8_aq83	0	27.7	70	0.3559	72	0.3477	-	-
dl_ay104	0	90.1	71	0.3560	71	0.3459	-	-
dl_db92	0	101.2	72	0.3560	76	0.3499	-	-
d8_ap93	0	17.9	73	0.3561	69	0.3452	-	-
d9_cb118	0	81.4	74	0.3563	82	0.3561	-	-
d9_aa60	0	77.8	75	0.3564	77	0.3521	-	-
d10_ah86	0	95.5	76	0.3568	73	0.3480	-	-
d8_az50	0	25	77	0.3572	70	0.3457	19	0.5326
d10_az101	0	87.7	78	0.3587	75	0.3485	-	-
dl_aa99	0	91.7	79	0.3590	81	0.3558	-	-
d10_db23	0	103.2	80	0.3609	78	0.3541	-	-
d9_ay59	0	71.4	81	0.3634	85	0.3580	-	-
dl_aq19	0	91.3	82	0.3642	80	0.3549	-	-
d9_aq60	0	81.3	83	0.3642	84	0.3572	-	-
d10_ay116	0	100.5	84	0.3644	79	0.3548	9	0.4099
d9_af15	0	81.3	85	0.3656	86	0.3590	-	-

continued...

structure	duplicates	ΔH	rank	VC-xPWDF (3h)	rank	VC-xPWDF (2m)	rank	autoFIDEL
d9_aa26	0	82.6	86	0.3668	95	0.3664	-	-
d9_az62	0	82.7	87	0.3669	83	0.3566	-	-
dl_av59	1	93	88	0.3670	90	0.3620	-	-
d9_af39	0	71.6	89	0.3680	89	0.3615	-	-
d9_av107	0	76.7	90	0.3695	91	0.3621	-	-
d9_cb65	0	77.6	91	0.3700	96	0.3674	-	-
d10_av11	1	100.4	92	0.3707	94	0.3652	-	-
d9_bb68	0	81.3	93	0.3717	87	0.3602	-	-
d8_au41	0	26.2	94	0.3718	88	0.3603	-	-
d10_aq81	0	93	95	0.3733	92	0.3628	-	-
d9_av100	0	78.9	96	0.3734	98	0.3677	-	-
dl_db35	1	89.4	97	0.3746	97	0.3675	-	-
dl_aa46	2	83.9	98	0.3749	93	0.3641	-	-
dl_af74	0	104.3	99	0.3765	100	0.3687	8	0.4036
dl_as86	0	93.3	100	0.3770	101	0.3696	-	-
d9_ba42	0	82.6	101	0.3777	102	0.3696	-	-
d8_aq31	0	28.6	102	0.3780	103	0.3696	-	-
d10_af78	0	100	103	0.3780	104	0.3707	-	-
dl_aq131	0	90.7	104	0.3783	99	0.3681	-	-
dl_as117	0	102.7	105	0.3789	107	0.3740	-	-
dl_ay95	0	97.2	106	0.3809	110	0.3764	-	-
d9_af10	0	72.2	107	0.3817	106	0.3734	-	-
d9_ba81	0	82.1	108	0.3839	109	0.3744	-	-
d10_bd34	1	101.5	109	0.3845	112	0.3783	-	-
d9_az101	0	81.1	110	0.3853	108	0.3744	-	-
d9_cd88	0	80.8	111	0.3857	119	0.3826	-	-
d9_ae82	0	76	112	0.3858	111	0.3775	-	-
dl_ae58	0	102.5	113	0.3859	105	0.3728	-	-
dl_au89	0	96.4	114	0.3871	113	0.3808	-	-
d9_ay2	0	69.6	115	0.3885	114	0.3812	-	-
d9_av77	0	81.9	116	0.3892	124	0.3847	-	-
dl_as108	0	86.8	117	0.3893	116	0.3823	-	-
d10_as78	0	89.2	118	0.3898	118	0.3824	-	-
dl_db48	0	100.6	119	0.3906	117	0.3823	-	-
d10_ah15	0	103.2	120	0.3906	123	0.3845	-	-
dl_ay80	0	98	121	0.3917	120	0.3836	14	0.4635
dl_az85	0	85.7	122	0.3921	115	0.3818	-	-
d9_aq87	0	75.6	123	0.3939	128	0.3880	-	-
d9_az55	0	72.7	124	0.3939	121	0.3836	-	-
dl_cb142	0	104.8	125	0.3940	129	0.3885	-	-
d10_cb82	0	95.5	126	0.3946	130	0.3886	22	0.5664
d8_ah80	0	28.1	127	0.3951	127	0.3876	-	-
d9_bh54	0	77.9	128	0.3952	125	0.3858	-	-
dl_bh101	1	101.9	129	0.3953	122	0.3841	-	-

continued...

structure	duplicates	ΔH	rank	VC-xPWDF (3h)	rank	VC-xPWDF (2m)	rank	autoFIDEL
d10_ah27	0	84.9	130	0.3964	133	0.3913	-	-
d10_cb137	0	96.1	131	0.3973	138	0.3935	-	-
d9_db107	0	81.4	132	0.3977	126	0.3860	-	-
d9_db8	0	77.7	133	0.3982	132	0.3911	-	-
d9_as34	0	69	134	0.3986	131	0.3907	-	-
d10_af59	0	87.3	135	0.3989	136	0.3915	-	-
d9_as42	0	82.5	136	0.3989	146	0.3975	-	-
d10_as58	0	92.8	137	0.3992	134	0.3914	-	-
dl_ah88	0	81	138	0.4011	144	0.3958	28	0.5916
dl_au38	1	98.3	139	0.4016	135	0.3915	-	-
dl_ay6	0	93.5	140	0.4018	139	0.3936	-	-
dl_aq66	0	86	141	0.4025	141	0.3948	-	-
d10_az14	1	94.3	142	0.4036	147	0.3977	-	-
d10_aq97	0	96.4	143	0.4041	140	0.3948	-	-
d9_af95	0	77.5	144	0.4043	143	0.3956	-	-
d9_ah36	0	80.4	145	0.4045	152	0.4007	-	-
dl_cb152	0	101.2	146	0.4049	151	0.4002	-	-
dl_bd53	1	95.5	147	0.4056	142	0.3954	-	-
d9_aa106	1	80.9	148	0.4057	145	0.3962	-	-
d9_bd48	1	80.2	149	0.4058	137	0.3926	-	-
dl_db29	0	80.3	150	0.4060	148	0.3978	-	-
dl_aq107	1	83.6	151	0.4081	150	0.3994	-	-
d10_aq83	0	81.7	152	0.4081	149	0.3989	-	-
dl_af108	0	97.7	153	0.4088	154	0.4027	-	-
d10_aq89	0	88.2	154	0.4098	153	0.4020	-	-
dl_cb76	0	93.8	155	0.4107	165	0.4070	-	-
d9_cc103	0	66.7	156	0.4110	161	0.4064	-	-
d10_bf56	1	99.7	157	0.4112	155	0.4036	-	-
d9_ah86	0	76.3	158	0.4112	156	0.4044	12	0.4459
dl_av75	0	98.3	159	0.4113	157	0.4047	-	-
d9_db6	1	77.1	160	0.4129	158	0.4053	-	-
dl_cb116	0	94.5	161	0.4144	166	0.4075	-	-
dl_av110	0	102.1	162	0.4145	167	0.4081	-	-
dl_cb46	0	101.2	163	0.4147	163	0.4066	-	-
dl_cb19	0	97.9	164	0.4153	164	0.4069	-	-
dl_cb105	0	88.2	165	0.4157	170	0.4096	-	-
d9_av49	0	81	166	0.4158	178	0.4114	-	-
dl_af60	0	81.6	167	0.4158	169	0.4084	-	-
dl_aq60	0	103.2	168	0.4158	160	0.4063	-	-
dl_bf50	0	81.6	169	0.4161	162	0.4064	-	-
d9_ay29	0	81.2	170	0.4174	159	0.4053	-	-
d10_ecod10	1	82.4	171	0.4175	168	0.4084	-	-
d9_as115	0	81.3	172	0.4177	182	0.4140	-	-
d10_cb84	0	102.2	173	0.4179	172	0.4099	-	-

continued. . .

structure	duplicates	ΔH	rank	VC-xPWDF (3h)	rank	VC-xPWDF (2m)	rank	autoFIDEL
d9_ba16	1	74.5	174	0.4182	179	0.4117	-	-
dl_ay67	1	96.1	175	0.4193	171	0.4098	-	-
d9_ap106	0	82.3	176	0.4194	177	0.4110	-	-
d10_cb121	0	98.3	177	0.4201	181	0.4129	-	-
dl_as101	0	94.2	178	0.4202	180	0.4120	-	-
dl_db25	0	85.2	179	0.4203	176	0.4109	-	-
d9_ay47	0	82.5	180	0.4208	173	0.4101	-	-
dl_av112	0	102.6	181	0.4214	185	0.4149	-	-
d9_ah65	0	77.3	182	0.4216	184	0.4143	17	0.5115
dl_cb97	0	93.6	183	0.4218	187	0.4153	-	-
d9_af72	1	69.4	184	0.4223	175	0.4108	-	-
dl_av21	0	99.9	185	0.4235	186	0.4152	-	-
d10_af16	1	97.9	186	0.4238	188	0.4157	-	-
d9_cc39	0	72.1	187	0.4239	196	0.4199	-	-
d9_db39	0	78.1	188	0.4240	174	0.4102	-	-
dl_av122	0	98.6	189	0.4242	194	0.4184	26	0.5869
d9_bh85	1	75.5	190	0.4248	192	0.4183	-	-
d9_cd49	0	82	191	0.4253	183	0.4140	-	-
d9_ay56	0	76.4	192	0.4260	190	0.4172	-	-
d9_af100	0	81.4	193	0.4261	189	0.4158	-	-
d9_ay93	0	76.9	194	0.4261	193	0.4183	-	-
dl_af97	1	94.5	195	0.4264	203	0.4221	-	-
d10_ba64	0	96.7	196	0.4268	191	0.4174	-	-
d9_ay104	0	75	197	0.4270	202	0.4220	-	-
d10_ay5	0	97.4	198	0.4276	195	0.4191	-	-
d8_cc9	0	29.8	199	0.4281	204	0.4222	-	-
d9_ah15	0	79.5	200	0.4282	199	0.4207	-	-
dl_cc102	0	103	201	0.4288	211	0.4272	-	-
d9_ah95	0	63	202	0.4294	206	0.4238	-	-
dl_bf56	1	91.5	203	0.4297	201	0.4220	-	-
dl_ba95	0	100	204	0.4301	197	0.4201	-	-
d9_bc65	0	78.9	205	0.4310	198	0.4207	-	-
dl_av135	0	103.2	206	0.4315	200	0.4218	-	-
dl_aq50	0	95.8	207	0.4322	205	0.4227	-	-
d9_ay90	0	79.2	208	0.4329	207	0.4239	-	-
dl_ah102	0	79.6	209	0.4336	209	0.4263	-	-
d10_bd3	1	99.9	210	0.4347	214	0.4296	-	-
DMANTL14	-	-	211	0.4352	208	0.4252	-	-
dl_bb47	1	88.4	212	0.4368	210	0.4270	-	-
dl_as105	0	96.5	213	0.4369	220	0.4332	-	-
d9_au97	0	77.8	214	0.4373	212	0.4273	-	-
d10_ap52	0	99.4	215	0.4389	216	0.4297	-	-
d9_af69	0	73.1	216	0.4394	215	0.4297	-	-
d9_az35	0	80.4	217	0.4408	225	0.4352	-	-

continued. . .

structure	duplicates	ΔH	rank	VC-xPWDF (3h)	rank	VC-xPWDF (2m)	rank	autoFIDEL
d10_cb38	0	96	218	0.4409	222	0.4342	-	-
d10_ba35	0	103.1	219	0.4412	217	0.4309	-	-
d9_cc58	0	71.1	220	0.4423	230	0.4384	-	-
dl_az34	3	100.4	221	0.4424	213	0.4295	-	-
d10_au55	0	93.4	222	0.4432	219	0.4320	-	-
d10_aq43	0	98.2	223	0.4434	221	0.4337	-	-
dl_bf28	0	98.6	224	0.4448	227	0.4359	7	0.3942
d9_ah101	0	74.3	225	0.4452	226	0.4357	-	-
d9_au22	0	74.2	226	0.4461	218	0.4320	-	-
d10_ay61	0	99.6	227	0.4465	224	0.4351	-	-
dl_ap21	1	104.8	228	0.4475	223	0.4346	4	0.3510
d8_ah79	0	29.1	229	0.4480	233	0.4393	-	-
d10_aq66	0	97.7	230	0.4482	239	0.4419	-	-
dl_ay77	1	100.6	231	0.4483	234	0.4407	-	-
dl_bc36	0	97.2	232	0.4488	232	0.4388	-	-
dl_az83	0	97.6	233	0.4495	242	0.4436	-	-
d9_aq101	0	72.3	234	0.4498	235	0.4412	-	-
dl_ah82	0	101.7	235	0.4498	231	0.4387	-	-
dl_ah45	0	96.1	236	0.4499	228	0.4382	-	-
dl_au9	0	88.5	237	0.4500	229	0.4383	-	-
d9_ah37	0	70.1	238	0.4502	238	0.4417	-	-
dl_ap65	0	93.8	239	0.4510	236	0.4413	-	-
dl_ah9	1	98.3	240	0.4512	245	0.4443	-	-
d10_bd58	0	88.5	241	0.4529	240	0.4421	-	-
dl_av97	0	94	242	0.4532	241	0.4428	16	0.5011
d10_ay109	0	103.2	243	0.4535	248	0.4495	-	-
dl_bd77	0	82.7	244	0.4546	243	0.4438	-	-
d9_bf5	0	75.2	245	0.4551	237	0.4414	-	-
dl_bh84	0	97.8	246	0.4552	251	0.4502	-	-
dl_ay110	0	95.1	247	0.4562	259	0.4526	-	-
dl_ay75	0	95.5	248	0.4562	244	0.4442	-	-
dl_ay40	0	102.9	249	0.4574	253	0.4506	-	-
d10_bh56	1	99.8	250	0.4578	249	0.4496	-	-
d10_ah72	0	101.2	251	0.4578	252	0.4503	-	-
d9_bc16	0	81.2	252	0.4581	247	0.4488	-	-
dl_av105	0	101.6	253	0.4590	257	0.4523	-	-
d9_ba88	0	72.2	254	0.4590	250	0.4498	-	-
d9_cd139	0	73.4	255	0.4599	264	0.4569	-	-
dl_aq85	0	92.8	256	0.4608	255	0.4517	-	-
d9_as124	0	81.1	257	0.4620	254	0.4510	-	-
d10_ah63	0	103.3	258	0.4628	246	0.4485	-	-
dl_bc52	0	104	259	0.4632	258	0.4524	-	-
dl_au75	0	100.5	260	0.4643	262	0.4568	-	-
dl_ay79	0	98.6	261	0.4644	256	0.4523	-	-

continued...

structure	duplicates	ΔH	rank	VC-xPWDF (3h)	rank	VC-xPWDF (2m)	rank	autoFIDEL
d8_ae54	0	24.9	262	0.4650	260	0.4544	15	0.4863
dl_ah68	0	97.8	263	0.4651	265	0.4581	-	-
d9_ap116	0	78.2	264	0.4651	267	0.4593	-	-
d9_af97	0	77.2	265	0.4652	263	0.4569	-	-
d9_ah109	0	80.7	266	0.4679	261	0.4556	-	-
d9_as35	0	77.6	267	0.4680	268	0.4593	-	-
d9_az89	0	74.5	268	0.4710	266	0.4583	-	-
dl_az27	0	96.2	269	0.4711	273	0.4651	-	-
d10_ap7	0	96.9	270	0.4729	270	0.4630	-	-
d9_av82	0	79.5	271	0.4735	272	0.4643	-	-
d8_ah86	0	26.3	272	0.4744	274	0.4652	-	-
d9_aq67	0	76.8	273	0.4756	271	0.4637	-	-
dl_ay46	0	102.5	274	0.4758	276	0.4654	-	-
d9_ay81	1	55.7	275	0.4761	277	0.4655	-	-
d10_aq76	0	85.1	276	0.4762	278	0.4658	-	-
dl_ay30	0	100.9	277	0.4763	282	0.4677	-	-
d9_ap102	0	79.4	278	0.4766	275	0.4654	-	-
d10_bh7	0	89.8	279	0.4771	280	0.4665	-	-
dl_ae33	0	99.6	280	0.4780	269	0.4623	-	-
d10_au81	1	102.2	281	0.4782	287	0.4704	-	-
d9_cb98	0	78.9	282	0.4786	285	0.4697	-	-
d9_ae86	0	79.5	283	0.4787	289	0.4714	-	-
d8_af56	0	27.8	284	0.4793	284	0.4689	-	-
dl_cb25	0	102.8	285	0.4795	290	0.4722	-	-
d9_bh4	0	82	286	0.4796	291	0.4727	-	-
dl_az76	0	80.6	287	0.4802	286	0.4697	-	-
d9_ap34	0	73	288	0.4812	283	0.4686	-	-
dl_cb11	0	100.2	289	0.4816	292	0.4728	-	-
dl_cc74	0	96.6	290	0.4822	279	0.4662	-	-
d9_aa88	1	80	291	0.4822	293	0.4738	-	-
d9_az37	0	74.5	292	0.4823	294	0.4741	-	-
d10_cc23	0	101.2	293	0.4828	281	0.4676	13	0.4485
d9_cc69	0	81.3	294	0.4830	297	0.4760	-	-
d9_au90	0	78.1	295	0.4834	295	0.4742	-	-
d9_au15	0	75.5	296	0.4854	296	0.4744	-	-
dl_cb96	0	104.8	297	0.4861	288	0.4713	-	-
dl_bd50	0	98	298	0.4870	301	0.4796	-	-
d9_db13	1	80.4	299	0.4872	299	0.4785	-	-
d9_bf59	0	81.8	300	0.4875	303	0.4807	-	-
dl_bb20	0	97	301	0.4875	300	0.4790	-	-
d10_bf47	0	100.1	302	0.4882	304	0.4811	-	-
dl_au14	0	87.3	303	0.4889	298	0.4767	-	-
dl_ay112	0	101.5	304	0.4896	305	0.4826	-	-
d9_aq85	0	79.5	305	0.4930	307	0.4828	-	-

continued...

structure	duplicates	ΔH	rank	VC-xPWDF (3h)	rank	VC-xPWDF (2m)	rank	autoFIDEL
d9_db57	0	76.7	306	0.4941	306	0.4826	-	-
dl_bb59	0	105	307	0.4942	313	0.4884	-	-
d9_az95	0	71.7	308	0.4945	315	0.4895	2	0.2865
d10_ap24	1	98.5	309	0.4945	302	0.4805	-	-
dl_ah60	0	96.9	310	0.4954	314	0.4890	-	-
d9_cc6	0	78	311	0.4964	318	0.4910	-	-
dl_ap119	1	96.8	312	0.4965	309	0.4841	-	-
dl_ce77	0	101.7	313	0.4968	310	0.4845	-	-
d10_au106	1	89.7	314	0.4973	311	0.4849	-	-
d10_cd107	1	95.8	315	0.4979	312	0.4876	-	-
d9_av72	0	80.8	316	0.4986	317	0.4906	-	-
d10_cb125	0	101.3	317	0.4990	308	0.4835	-	-
d9_ay46	0	73.5	318	0.5006	319	0.4910	-	-
d9_av99	0	69.1	319	0.5014	316	0.4896	-	-
dl_bf69	0	95.4	320	0.5017	326	0.4946	-	-
d9_ay103	0	80.2	321	0.5029	333	0.4978	-	-
d10_cc63	0	98.1	322	0.5039	325	0.4946	-	-
d9_az108	1	65.1	323	0.5040	320	0.4934	-	-
d9_bh58	0	78.9	324	0.5041	321	0.4935	-	-
dl_ay55	0	93.5	325	0.5047	328	0.4960	-	-
dl_ae63	0	92.9	326	0.5056	327	0.4951	-	-
d10_cb83	0	93.7	327	0.5056	322	0.4935	-	-
d9_bf45	0	71.1	328	0.5056	323	0.4937	-	-
d10_bb28	0	99.5	329	0.5062	337	0.4999	-	-
d9_aq35	0	71.4	330	0.5064	324	0.4944	-	-
d10_ay21	0	96.1	331	0.5064	331	0.4968	-	-
dl_av69	0	104.1	332	0.5064	330	0.4964	-	-
d9_cb122	0	77.1	333	0.5064	329	0.4964	20	0.5477
dl_cb121	0	103.8	334	0.5071	334	0.4995	-	-
d10_au92	2	73.3	335	0.5073	332	0.4970	-	-
d9_au77	0	82.3	336	0.5077	335	0.4997	-	-
dl_ba90	0	97	337	0.5080	339	0.5017	-	-
d10_ay107	0	99	338	0.5092	338	0.5005	-	-
d9_cd142	0	77.4	339	0.5119	344	0.5039	-	-
d9_cb81	0	55.7	340	0.5121	341	0.5024	-	-
d10_bh86	0	97.5	341	0.5129	345	0.5047	-	-
d10_ae57	0	93.7	342	0.5141	343	0.5029	-	-
d9_ay65	0	82.6	343	0.5150	351	0.5076	-	-
dl_ay121	0	91.8	344	0.5150	346	0.5051	-	-
d10_af69	0	101.6	345	0.5153	336	0.4999	-	-
d9_aq15	0	67.9	346	0.5153	340	0.5020	24	0.5760
d9_cc79	0	81.8	347	0.5163	353	0.5080	-	-
d10_ae32	0	95.5	348	0.5164	356	0.5095	-	-
d9_ay69	0	80.9	349	0.5165	349	0.5063	-	-

continued...

structure	duplicates	ΔH	rank	VC-xPWDF (3h)	rank	VC-xPWDF (2m)	rank	autoFIDEL
dl_bc84	0	100.6	350	0.5173	348	0.5054	-	-
d9_cc49	0	78.9	351	0.5174	354	0.5087	-	-
dl_au49	0	98.3	352	0.5176	359	0.5107	-	-
dl_cb82	0	93.7	353	0.5178	347	0.5054	-	-
dl_bh78	0	92.5	354	0.5180	355	0.5095	-	-
d10_af89	0	101.4	355	0.5183	342	0.5029	-	-
d9_ah66	0	79.5	356	0.5187	350	0.5072	-	-
dl_au108	1	87.5	357	0.5198	352	0.5077	-	-
d9_ap113	0	77.4	358	0.5199	361	0.5114	-	-
d9_aq57	0	79.2	359	0.5210	362	0.5118	-	-
d9_cc7	0	64.9	360	0.5232	366	0.5167	-	-
d9_av43	0	77	361	0.5236	357	0.5103	-	-
d9_ah127	1	77.5	362	0.5242	360	0.5114	-	-
dl_bh4	0	92.7	363	0.5244	365	0.5160	-	-
dl_af80	0	103.7	364	0.5260	358	0.5106	-	-
d9_av39	0	76	365	0.5263	370	0.5216	-	-
d10_cd56	0	88.6	366	0.5270	363	0.5139	-	-
d9_bh42	0	77.9	367	0.5283	364	0.5141	-	-
dl_bh32	0	99.6	368	0.5289	368	0.5197	-	-
d9_ay49	0	80.5	369	0.5308	371	0.5225	-	-
d9_db20	0	78.5	370	0.5314	369	0.5213	-	-
d9_cb97	0	73.5	371	0.5320	367	0.5195	21	0.5591
d9_ba50	0	76.7	372	0.5344	372	0.5227	-	-
dl_ay68	0	103.5	373	0.5348	376	0.5258	-	-
d9_ah117	0	78.5	374	0.5354	374	0.5252	-	-
dl_aq118	0	96.6	375	0.5360	373	0.5235	-	-
d9_av44	0	74	376	0.5370	377	0.5262	29	0.6171
dl_az81	0	99.6	377	0.5372	381	0.5303	-	-
d9_bh86	0	82.7	378	0.5372	380	0.5287	-	-
dl_ce72	0	100.7	379	0.5375	375	0.5257	-	-
d9_aq54	0	78.3	380	0.5401	379	0.5275	-	-
d10_ah46	0	100.1	381	0.5407	378	0.5273	-	-
d9_cc70	1	74.6	382	0.5416	382	0.5303	-	-
d9_as16	0	72.8	383	0.5416	383	0.5312	-	-
d9_bb95	0	82	384	0.5420	384	0.5341	-	-
d10_as94	0	90.8	385	0.5438	386	0.5359	-	-
dl_cd62	0	101.6	386	0.5502	385	0.5354	-	-
d9_cb70	0	61.8	387	0.5504	387	0.5371	-	-
d10_bf87	0	90.7	388	0.5504	396	0.5455	-	-
d9_cb128	0	79.2	389	0.5508	394	0.5451	-	-
dl_as115	0	87	390	0.5530	395	0.5451	-	-
d9_ce58	0	71.4	391	0.5530	388	0.5411	-	-
d10_ay108	1	80.5	392	0.5542	391	0.5434	-	-
dl_bf98	0	103.2	393	0.5543	398	0.5457	-	-

continued. . .

structure	duplicates	ΔH	rank	VC-xPWDF (3h)	rank	VC-xPWDF (2m)	rank	autoFIDEL
dl_ay49	0	99.2	394	0.5553	399	0.5486	-	-
dl_ah99	0	103.9	395	0.5562	389	0.5413	23	0.5668
d10_cb150	0	96.8	396	0.5580	392	0.5449	-	-
d9_cb111	0	77.2	397	0.5581	397	0.5456	-	-
d9_aq98	0	81.7	398	0.5583	390	0.5431	-	-
d9_cb95	0	80.3	399	0.5594	393	0.5449	-	-
d10_ay96	0	99.2	400	0.5637	403	0.5568	-	-
d9_as82	1	71.6	401	0.5638	400	0.5500	-	-
d9_ay35	0	71	402	0.5646	401	0.5555	-	-
d9_ay40	0	76.6	403	0.5648	402	0.5564	-	-
d9_cd17	0	72.3	404	0.5699	404	0.5573	-	-
d10_cc41	0	93.9	405	0.5709	414	0.5685	-	-
d9_ce56	0	72.8	406	0.5720	413	0.5664	-	-
dl_cc36	0	90.9	407	0.5733	415	0.5707	-	-
d9_bc72	0	74.6	408	0.5735	406	0.5620	-	-
dl_bh8	0	102.3	409	0.5736	412	0.5659	-	-
d9_cc54	0	80.5	410	0.5745	411	0.5658	-	-
dl_aq44	0	102.7	411	0.5745	409	0.5650	-	-
dl_bf37	0	103.5	412	0.5755	405	0.5608	-	-
d9_af42	0	73.8	413	0.5758	407	0.5630	-	-
d9_au101	0	81.8	414	0.5770	408	0.5648	-	-
dl_cc49	0	97.4	415	0.5774	419	0.5739	-	-
d10_cd89	0	101.5	416	0.5787	410	0.5652	-	-
d9_ce51	0	80.7	417	0.5811	418	0.5733	-	-
d10_az65	0	86.5	418	0.5814	416	0.5714	-	-
dl_cc47	0	103.4	419	0.5843	417	0.5726	-	-
d9_cd92	1	80.7	420	0.5860	426	0.5806	-	-
d10_cc114	0	102.9	421	0.5861	420	0.5748	-	-
d10_bh50	0	96	422	0.5867	423	0.5789	-	-
d9_cc84	0	81.8	423	0.5887	422	0.5788	25	0.5829
dl_bh64	0	92.8	424	0.5895	428	0.5817	-	-
d9_ap23	0	75.3	425	0.5904	425	0.5797	-	-
dl_cc37	0	104.8	426	0.5910	421	0.5769	-	-
dl_ay5	0	100.9	427	0.5924	432	0.5828	-	-
dl_cc39	0	99.6	428	0.5929	430	0.5826	-	-
d10_ay22	0	96.8	429	0.5930	429	0.5819	-	-
d10_cc50	0	91.1	430	0.5933	434	0.5853	-	-
d9_ay119	0	76.8	431	0.5935	431	0.5826	-	-
dl_cd132	0	104.7	432	0.5939	424	0.5793	-	-
d9_ap107	0	66.5	433	0.5948	433	0.5836	-	-
dl_az90	0	83.3	434	0.5958	435	0.5860	-	-
d9_cb127	0	78.4	435	0.5965	427	0.5813	-	-
d9_ce34	0	69.6	436	0.5982	436	0.5895	-	-
d8_cc105	0	27.1	437	0.5988	440	0.5917	-	-

continued. . .

structure	duplicates	ΔH	rank	VC-xPWDF (3h)	rank	VC-xPWDF (2m)	rank	autoFIDEL
d9_ce112	0	79.7	438	0.5992	442	0.5922	-	-
d9_cd27	0	76.2	439	0.5999	438	0.5903	-	-
d9_cc86	0	75.6	440	0.5999	445	0.5930	-	-
d10_av96	1	95.8	441	0.6006	437	0.5897	-	-
dl_cc69	0	85.9	442	0.6016	446	0.5930	-	-
d10_cc69	0	101.9	443	0.6019	444	0.5924	-	-
d9_av53	0	82.3	444	0.6034	443	0.5922	-	-
d9_aq129	0	79	445	0.6043	441	0.5922	-	-
dl_cc86	0	93.3	446	0.6050	450	0.5967	-	-
dl_cb35	0	92.1	447	0.6053	439	0.5916	-	-
dl_cd54	0	101.2	448	0.6053	452	0.5983	-	-
d9_cb11	0	75.4	449	0.6058	447	0.5955	-	-
dl_bb51	0	99.2	450	0.6072	451	0.5982	-	-
dl_ap128	1	100.6	451	0.6089	454	0.6012	-	-
d10_cb108	0	96.2	452	0.6094	448	0.5959	-	-
dl_av95	0	102.5	453	0.6105	449	0.5961	-	-
d9_cb49	0	52.9	454	0.6110	453	0.6008	-	-
dl_cb156	0	77.6	455	0.6148	455	0.6037	-	-
dl_ap33	1	102.2	456	0.6154	457	0.6059	-	-
d9_ba8	0	79.2	457	0.6174	456	0.6042	-	-
dl_cc4	0	103.9	458	0.6202	458	0.6069	-	-
d9_cc43	0	77.8	459	0.6215	459	0.6087	-	-
d9_av42	0	74.4	460	0.6222	461	0.6115	-	-
d10_cb52	0	73.4	461	0.6232	462	0.6121	-	-
d9_aq138	1	82.2	462	0.6236	464	0.6136	-	-
dl_cd138	0	83.4	463	0.6240	460	0.6104	-	-
d10_cc10	0	94.9	464	0.6254	471	0.6217	-	-
d10_cb132	0	87	465	0.6257	463	0.6124	-	-
d10_ay98	0	93.6	466	0.6269	467	0.6183	-	-
dl_av121	0	103.5	467	0.6274	465	0.6148	-	-
dl_cb33	0	96.8	468	0.6282	466	0.6179	-	-
dl_bc74	0	97.3	469	0.6289	468	0.6203	-	-
d9_cc52	0	64.8	470	0.6300	475	0.6255	-	-
dl_ay19	0	101.8	471	0.6314	469	0.6206	-	-
d9_cc26	0	72	472	0.6321	473	0.6252	-	-
dl_ay85	0	87.6	473	0.6339	474	0.6254	-	-
d9_ce26	0	82.5	474	0.6360	485	0.6308	-	-
dl_cc10	0	90.5	475	0.6361	491	0.6323	-	-
dl_cd71	0	103.3	476	0.6366	472	0.6249	-	-
dl_cc27	0	102.6	477	0.6373	484	0.6307	-	-
dl_cb40	0	85.9	478	0.6374	476	0.6267	-	-
d10_cd76	0	101.2	479	0.6378	477	0.6277	-	-
d9_cd59	0	74.5	480	0.6384	481	0.6301	-	-
dl_cc28	0	98.8	481	0.6386	470	0.6216	-	-

continued…

structure	duplicates	ΔH	rank	VC-xPWDF (3h)	rank	VC-xPWDF (2m)	rank	autoFIDEL
dl_aq92	0	87.1	482	0.6387	480	0.6300	-	-
dl_cc114	0	96.6	483	0.6389	492	0.6335	-	-
dl_au90	0	100.1	484	0.6391	479	0.6297	-	-
d8_cc27	0	27.9	485	0.6395	482	0.6304	-	-
dl_cd113	0	91.3	486	0.6401	483	0.6304	-	-
d10_aq105	0	90.3	487	0.6402	489	0.6318	-	-
d9_aq104	0	76.1	488	0.6405	478	0.6285	-	-
dl_ce14	0	100.4	489	0.6414	495	0.6343	-	-
d10_cb23	0	82.6	490	0.6416	486	0.6311	-	-
d9_cb89	0	79.8	491	0.6421	488	0.6318	-	-
d10_cc20	1	92.8	492	0.6424	487	0.6312	-	-
dl_as113	0	104.3	493	0.6443	494	0.6339	-	-
d9_cc27	0	73.7	494	0.6447	493	0.6337	-	-
d9_cd76	0	80.7	495	0.6451	501	0.6411	-	-
dl_aq111	0	96.3	496	0.6452	497	0.6365	-	-
d9_aq111	0	75.5	497	0.6456	490	0.6320	-	-
d10_cc19	0	100.4	498	0.6466	499	0.6397	-	-
dl_cb73	0	84.2	499	0.6498	496	0.6348	-	-
dl_bf54	0	86	500	0.6499	500	0.6401	-	-
d10_cb91	0	90.7	501	0.6514	498	0.6368	-	-
d9_cc16	0	71.7	502	0.6518	504	0.6456	-	-
dl_cc53	0	97.1	503	0.6519	503	0.6452	-	-
d10_av61	0	93.1	504	0.6528	502	0.6426	-	-
dl_cc21	0	93.8	505	0.6539	507	0.6476	-	-
d9_cc106	0	78.8	506	0.6555	508	0.6478	-	-
d10_ce94	0	95.3	507	0.6555	505	0.6460	-	-
d9_aq143	0	65.5	508	0.6584	506	0.6465	-	-
dl_cb84	0	100.6	509	0.6586	510	0.6503	-	-
dl_cb21	0	100.9	510	0.6594	509	0.6492	-	-
d9_aq38	0	82.2	511	0.6603	511	0.6504	-	-
dl_cb119	0	103	512	0.6607	512	0.6507	-	-
dl_cc50	0	101.7	513	0.6648	513	0.6534	5	0.3556
dl_av62	0	94.1	514	0.6656	514	0.6548	-	-
dl_ae7	1	90.1	515	0.6673	517	0.6591	-	-
d10_bf72	0	91.8	516	0.6690	515	0.6589	-	-
d10_aq16	0	100.6	517	0.6707	518	0.6627	-	-
dl_db76	0	87.7	518	0.6712	516	0.6591	-	-
dl_aq77	0	98.6	519	0.6725	519	0.6640	-	-
dl_ce84	0	101.2	520	0.6745	521	0.6650	-	-
dl_ce27	0	102.5	521	0.6759	524	0.6683	-	-
d10_aq48	1	103.3	522	0.6768	527	0.6705	-	-
d10_cc76	0	97.4	523	0.6782	520	0.6643	-	-
d9_ap119	0	76.1	524	0.6789	523	0.6677	-	-
d8_ce1	0	29.8	525	0.6791	525	0.6687	-	-

continued. . .

structure	duplicates	ΔH	rank	VC-xPWDF (3h)	rank	VC-xPWDF (2m)	rank	autoFIDEL
dl_cc40	0	92.8	526	0.6799	522	0.6657	-	-
dl_cd109	0	103	527	0.6806	529	0.6720	-	-
d9_au81	0	79.7	528	0.6809	528	0.6715	-	-
dl_cc71	0	103.7	529	0.6809	531	0.6750	-	-
d9_ap66	0	69.2	530	0.6824	533	0.6758	-	-
d9_cb77	0	72.9	531	0.6827	526	0.6695	-	-
dl_cd102	0	96.5	532	0.6838	530	0.6730	-	-
d9_cd6	0	76.2	533	0.6866	532	0.6755	-	-
d9_cc89	0	80.2	534	0.6920	535	0.6854	-	-
d9_as61	0	70.8	535	0.6931	534	0.6847	-	-
d9_cc53	0	82.5	536	0.7007	537	0.6963	-	-
d9_cc65	0	75.2	537	0.7062	536	0.6950	-	-
dl_cd80	0	86.4	538	0.7062	539	0.6989	-	-
d10_cb152	0	101.7	539	0.7075	540	0.6996	-	-
d10_cb89	1	87.3	540	0.7085	541	0.7012	-	-
d8_bc81	0	0	541	0.7100	538	0.6984	11	0.4429
dl_av93	0	96.3	542	0.7147	542	0.7034	-	-
d10_cc31	0	101.7	543	0.7183	545	0.7125	-	-
d10_ba53	0	96.5	544	0.7193	543	0.7092	-	-
dl_cc109	0	97.3	545	0.7212	546	0.7155	-	-
d10_as38	0	100.5	546	0.7217	544	0.7105	-	-
d10_cd59	0	101	547	0.7489	547	0.7398	-	-
d9_ba49	0	79.2	548	0.7519	548	0.7420	-	-
d9_az105	0	73.8	549	0.7687	549	0.7606	-	-
d10_ay105	0	93.1	550	0.8927	550	0.8869	-	-

Table S13: Summary of data for progesterone. Relative enthalpy is given in kJ/mol, ranks are according to the next-right column, autoFIDEL comparisons were made with the default program parameters and the experimental powder diffractograms collected over 3 hrs.

structure	duplicates	ΔH	rank	VC-xPWDF (3h)	rank	VC-xPWDF (2m)	rank	autoFIDEL
PROGST12	-	-	1	0.0416	1	0.0447	-	-
PROGST10	-	-	2	0.0428	2	0.0463	1	0.0895
m65_aq46	0	9.8	3	0.0490	3	0.0528	2	0.0918
m65_ak98	0	13.7	4	0.1116	4	0.1128	-	-
opt_ca33	0	7	5	0.1122	5	0.1143	13	0.3751
opt_dd55	0	9.8	6	0.1174	6	0.1151	-	-
m110_ca2	0	9.8	7	0.1183	8	0.1209	-	-
opt_ca84	0	9.6	8	0.1184	7	0.1194	-	-
opt_am6	0	10.8	9	0.1243	9	0.1230	-	-
m190_ca81	0	12.6	10	0.1272	10	0.1284	-	-
opt_ab96	0	14.2	11	0.1273	11	0.1312	-	-
m190_ak10	0	12.7	12	0.1373	12	0.1362	-	-
opt_av5	0	13.4	13	0.1391	13	0.1375	-	-
opt_ab50	0	13.4	14	0.1430	15	0.1455	-	-
m80_aq13	0	13	15	0.1436	14	0.1428	16	0.4232
m80_ca6	0	11.8	16	0.1470	17	0.1473	-	-
opt_ab97	0	8.4	17	0.1490	16	0.1464	-	-
opt_ab36	0	7.7	18	0.1491	18	0.1508	-	-
opt_ak50	0	13.5	19	0.1518	19	0.1517	11	0.3521
opt_ab114	0	9.4	20	0.1541	20	0.1521	-	-
m80_ab115	0	14.1	21	0.1566	23	0.1593	-	-
m190_ca6	0	8.2	22	0.1571	21	0.1575	-	-
opt_ca9	0	7.6	23	0.1580	22	0.1589	-	-
m190_ab122	0	13.5	24	0.1626	24	0.1654	-	-
m190_ab89	0	12.8	25	0.1689	25	0.1660	24	0.5042
opt_ab111	0	12.1	26	0.1723	27	0.1767	-	-
opt_ab108	0	11.2	27	0.1745	26	0.1761	27	0.6123
m190_ca55	0	13.7	28	0.1775	29	0.1784	-	-
m65_am44	0	11.6	29	0.1786	28	0.1769	6	0.2677
opt_am2	0	13.9	30	0.1787	31	0.1822	-	-
m65_ca10	0	9.2	31	0.1805	30	0.1815	5	0.2544
opt_fa44	0	14.7	32	0.1850	32	0.1842	20	0.4484
m190_ak12	0	9.6	33	0.1876	34	0.1897	-	-
m65_ak70	0	10.1	34	0.1878	33	0.1879	-	-
opt_cc95	0	14.3	35	0.1905	39	0.1940	-	-
opt_ab94	0	9.9	36	0.1907	38	0.1938	-	-
opt_ca123	0	13	37	0.1913	36	0.1924	-	-
opt_de16	0	14	38	0.1929	40	0.1942	-	-
opt_ab60	0	14.2	39	0.1935	37	0.1930	-	-
m190_dd92	0	14.5	40	0.1941	35	0.1910	-	-
opt_ab123	0	13.3	41	0.1997	41	0.2033	-	-

continued. . .

structure	duplicates	ΔH	rank	VC-xPWDF (3h)	rank	VC-xPWDF (2m)	rank	autoFIDEL
opt_am58	0	14.9	42	0.2053	43	0.2057	-	-
m65_am2	0	4.6	43	0.2071	42	0.2045	-	-
m80_ca35	0	13.1	44	0.2081	44	0.2106	-	-
opt_ca120	0	10.8	45	0.2083	45	0.2111	-	-
m65_ai3	0	10.9	46	0.2148	46	0.2128	25	0.5861
m190_ca71	0	6.4	47	0.2164	47	0.2140	-	-
m190_am8	0	13.9	48	0.2218	48	0.2185	-	-
opt_ca76	0	7.3	49	0.2228	50	0.2233	-	-
opt_am4	0	13.3	50	0.2258	53	0.2254	-	-
opt_am69	0	13	51	0.2263	52	0.2250	-	-
m190_aq60	0	13.8	52	0.2265	49	0.2233	14	0.3788
opt_dd2	0	7.9	53	0.2299	54	0.2257	-	-
opt_ab56	0	14.5	54	0.2302	57	0.2298	-	-
m80_ak24	0	10.6	55	0.2302	55	0.2284	23	0.4620
opt_ai41	0	13.6	56	0.2317	51	0.2241	-	-
opt_fc41	0	13.6	57	0.2322	56	0.2295	-	-
opt_aq29	0	14.4	58	0.2346	58	0.2362	-	-
opt_dd32	0	11.4	59	0.2417	59	0.2366	8	0.3143
m65_dc11	0	9.3	60	0.2460	60	0.2444	-	-
m80_ca97	0	14.2	61	0.2467	61	0.2516	-	-
opt_am65	0	13.3	62	0.2525	62	0.2525	-	-
opt_ca50	0	6.5	63	0.2542	63	0.2536	-	-
opt_ca125	0	7	64	0.2574	64	0.2582	-	-
m80_ab54	0	11.6	65	0.2595	68	0.2626	-	-
opt_am5	0	10.2	66	0.2623	65	0.2589	-	-
m190_ca61	0	13.2	67	0.2629	66	0.2604	-	-
opt_av1	0	7	68	0.2638	67	0.2615	-	-
opt_aq38	0	12.5	69	0.2665	69	0.2629	-	-
opt_ai5	0	14.3	70	0.2680	71	0.2678	-	-
opt_ca25	0	10.2	71	0.2703	72	0.2694	-	-
m80_az3	0	11.3	72	0.2703	70	0.2661	-	-
opt_ak14	0	13.5	73	0.2706	73	0.2705	-	-
opt_ab78	0	11.5	74	0.2715	74	0.2749	-	-
opt_ca95	0	8.2	75	0.2734	75	0.2751	-	-
m65_fa73	0	11.2	76	0.2754	76	0.2755	-	-
opt_de56	0	3	77	0.2794	77	0.2788	-	-
opt_aq104	0	14.6	78	0.2907	78	0.2881	-	-
opt_cb92	0	11.9	79	0.2939	84	0.2958	-	-
opt_fa1	0	9.5	80	0.2951	81	0.2929	-	-
m190_fc67	0	10.3	81	0.2957	80	0.2891	-	-
m80_fa44	0	11.9	82	0.2965	82	0.2941	10	0.3432
m190_de6	0	11	83	0.2971	79	0.2889	-	-
opt_ca21	0	12.3	84	0.2975	86	0.3020	-	-
opt_al1	0	13	85	0.3015	85	0.2962	-	-

continued...

structure	duplicates	ΔH	rank	VC-xPWDF (3h)	rank	VC-xPWDF (2m)	rank	autoFIDEL
m80_ai109	0	12.2	86	0.3017	83	0.2943	7	0.2993
m110_fc18	0	13.9	87	0.3044	87	0.3028	-	-
m65_ca14	0	13.7	88	0.3132	88	0.3129	-	-
opt_ak42	0	14.9	89	0.3142	89	0.3155	22	0.4608
m65_am26	0	13	90	0.3241	91	0.3227	-	-
m80_fc44	0	14.4	91	0.3244	92	0.3242	17	0.4254
opt_fc9	0	15	92	0.3245	90	0.3225	-	-
opt_aq94	0	8.4	93	0.3272	93	0.3281	-	-
m65_fc90	0	7.6	94	0.3295	94	0.3282	-	-
opt_ca42	0	13.3	95	0.3389	95	0.3333	-	-
opt_ab112	0	14.3	96	0.3390	96	0.3337	4	0.2480
opt_bd1	0	12	97	0.3394	100	0.3389	-	-
opt_al80	0	13.6	98	0.3396	98	0.3361	-	-
opt_ab35	0	11.3	99	0.3397	97	0.3354	-	-
opt_de93	0	13.2	100	0.3422	99	0.3378	15	0.3864
PROGST13	-	-	101	0.3426	101	0.3434	-	-
m190_ak5	0	14.1	102	0.3468	102	0.3459	-	-
m80_ak4	0	14.6	103	0.3484	103	0.3480	-	-
opt_ab104	0	12.9	104	0.3568	105	0.3529	-	-
opt_fa87	0	9.8	105	0.3584	104	0.3519	-	-
m65_ab49	0	11.4	106	0.3593	107	0.3602	-	-
m110_fc11	0	12.8	107	0.3626	106	0.3579	-	-
m190_am96	0	14.6	108	0.3771	108	0.3721	-	-
opt_ak101	0	12.9	109	0.3816	109	0.3783	3	0.2440
m65_ak49	0	12.8	110	0.3831	110	0.3802	-	-
opt_ai42	0	13.5	111	0.3871	111	0.3848	18	0.4360
opt_ak4	0	14.3	112	0.3949	112	0.3892	-	-
m65_fc88	0	13.9	113	0.3954	113	0.3899	21	0.4494
opt_fc29	0	11.9	114	0.3969	115	0.3972	-	-
opt_ai6	0	12	115	0.3972	114	0.3936	-	-
m190_ak1	0	12.5	116	0.3988	117	0.3994	-	-
m80_ak23	0	14.7	117	0.3998	116	0.3991	-	-
m80_ak121	0	14.1	118	0.4029	118	0.4010	-	-
m65_ak42	0	10.1	119	0.4093	119	0.4090	-	-
opt_cb47	0	10.8	120	0.4140	120	0.4094	-	-
opt_ak9	0	12	121	0.4151	121	0.4127	-	-
opt_fa39	0	9.5	122	0.4157	122	0.4129	-	-
opt_ak1	0	1.5	123	0.4264	123	0.4238	-	-
m110_fc29	0	13.7	124	0.4265	125	0.4257	-	-
opt_ab15	0	9.5	125	0.4293	124	0.4242	9	0.3147
opt_ab101	0	14	126	0.4401	126	0.4352	-	-
m110_ak100	0	12.5	127	0.4522	127	0.4507	-	-
m80_ak119	0	11.1	128	0.4575	128	0.4557	-	-
opt_cc42	0	14	129	0.4584	129	0.4595	-	-

continued. . .

structure	duplicates	ΔH	rank	VC-xPWDF (3h)	rank	VC-xPWDF (2m)	rank	autoFIDEL
opt_ak16	0	0	130	0.4650	131	0.4630	-	-
opt_fa3	0	11.8	131	0.4656	130	0.4610	19	0.4472
opt_ak113	0	5.1	132	0.4662	132	0.4638	-	-
opt_ab79	0	13.1	133	0.4678	133	0.4650	-	-
opt_fc3	0	12.8	134	0.4793	134	0.4716	-	-
m80_ak13	0	12.6	135	0.4885	135	0.4865	-	-
m110_fc47	0	14	136	0.4971	136	0.4950	-	-
opt_fc52	0	14.3	137	0.5085	137	0.5090	-	-
opt_ab116	0	12.3	138	0.5160	138	0.5111	-	-
opt_fc31	0	14.7	139	0.5180	139	0.5152	26	0.5884
opt_au2	0	13.5	140	0.5222	140	0.5171	-	-
m65_ca120	0	7.8	141	0.5247	141	0.5216	12	0.3741
opt_fc8	0	14.2	142	0.5343	142	0.5304	-	-
opt_fa64	0	10.8	143	0.5412	143	0.5367	-	-
m110_cb6	0	13.8	144	0.5461	144	0.5486	-	-
opt_cc121	0	11.7	145	0.5465	145	0.5492	-	-
m110_ak36	0	14.8	146	0.5804	147	0.5776	-	-
opt_dc51	0	14.5	147	0.5821	146	0.5760	-	-
opt_cc11	0	14.1	148	0.5891	148	0.5814	-	-
opt_cb48	0	10.4	149	0.5951	149	0.5910	-	-
opt_cb25	0	9.6	150	0.6031	150	0.6001	-	-
opt_ai62	0	7.2	151	0.6545	151	0.6502	-	-
m65_ak3	0	11	152	0.6580	152	0.6537	-	-

References

(1) X'pert Data Collector Software, version 5.3; PANalytical B.V.: Almelo, The Netherlands, 2014.

(2) Lancaster, R. W.; Karamertzanis, P. G.; Hulme, A. T.; Tocher, D. A.; Covey, D. F.; Price, S. L. *Chem. Commun.* **2006**, 4921–4923.

(3) Price, S. L.; Wibley, K. S. *J. Phys. Chem. A* **1997**, *101*, 2198–2206.

(4) Issa, N.; Barnett, S. A.; Mohamed, S.; Braun, D. E.; Copley, R. C.; Tocher, D. A.; Price, S. L. *CrystEngComm* **2012**, *14*, 2454–2464.

(5) Habgood, M. *Cryst. Growth Des.* **2011**, *11*, 3600–3608.

(6) Frisch, M. J. et al., Gaussian Inc. Wallingford CT 2010.

(7) Stone, A.; Alderton, M. *Mol. Phys.* **1985**, *56*, 1047–1064.

(8) Holden, J. R.; Du, Z.; Ammon, H. L. *J. Comput. Chem.* **1993**, *14*, 422–437.

(9) Karamertzanis, P.; Pantelides, C. *Mol. Phys.* **2007**, *105*, 273–291.

(10) Williams, D. E. *J. Comput. Chem.* **2001**, *22*, 1154–1166.

(11) Coombes, D. S.; Price, S. L.; Willock, D. J.; Leslie, M. *J. Phys. Chem.* **1996**, *100*, 7352–7360.

(12) Willock, D. J.; Price, S. L.; Leslie, M.; Catlow, C. R. A. *J. Comput. Chem.* **1995**, *16*, 628–647.

(13) Price, S. L.; Leslie, M.; Welch, G. W. A.; Habgood, M.; Price, L. S.; Karamertzanis, P. G.; Day, G. M. *Phys. Chem. Chem. Phys.* **2010**, *12*, 8478–8490.

(14) Kazantsev, A.; Karamertzanis, P.; Pantelides, C. Molecular System Engineering, edited by CS Adjiman & A. Galindo, Vol. 6, 2010.

(15) Mayo, R. A.; Otero-de-la-Roza, A.; Johnson, E. R. *CrystEngComm* **2022**, *24*, 8326–8338.

(16) Otero-de-la-Roza, A.; Johnson, E. R.; Luaña, V. *Comput. Phys. Commun.* **2014**, *185*, 1007–1018.

(17) De Gelder, R.; Wehrens, R.; Hageman, J. A. *J. Comput. Chem.* **2001**, *22*, 273–289.

(18) Habermehl, S.; Mörschel, P.; Eisenbrandt, P.; Hammer, S. M.; Schmidt, M. U. *Acta Crystallogr.* **2014**, ***B****70*, 347–359.

(19) Motherwell, S.; Chisholm, J. A. *J. Appl. Cryst.* **2005**, *38*, 228–231.

(20) Macrae, C. F.; Sovago, I.; Cottrell, S. J.; Galek, P. T. A.; McCabe, P.; Pidcock, E.; Platings, M.; Shields, G. P.; Stevens, J. S.; Towler, M.; Wood, P. A. *J. Appl. Cryst.* **2020**, *53*, 226–235.
